# Are drug targets with genetic support twice as likely to be approved? Revised estimates of the impact of genetic support for drug mechanisms on the probability of drug approval

**DOI:** 10.1371/journal.pgen.1008489

**Published:** 2019-12-12

**Authors:** Emily A. King, J. Wade Davis, Jacob F. Degner

**Affiliations:** Department of Computational Genomics, AbbVie, North Chicago, Illinois, United States of America; Regeneron Genetics Center, UNITED STATES

## Abstract

Despite strong vetting for disease activity, only 10% of candidate new molecular entities in early stage clinical trials are eventually approved. Analyzing historical pipeline data, Nelson et al. 2015 (Nat. Genet.) concluded pipeline drug targets with human genetic evidence of disease association are twice as likely to lead to approved drugs. Taking advantage of recent clinical development advances and rapid growth in GWAS datasets, we extend the original work using updated data, test whether genetic evidence predicts future successes and introduce statistical models adjusting for target and indication-level properties. Our work confirms drugs with genetically supported targets were more likely to be successful in Phases II and III. When causal genes are clear (Mendelian traits and GWAS associations linked to coding variants), we find the use of human genetic evidence increases approval by greater than two-fold, and, for Mendelian associations, the positive association holds prospectively. Our findings suggest investments into genomics and genetics are likely to be beneficial to companies deploying this strategy.

## Author summary

The growth of human genetics resources has the potential to help us develop better drugs. By looking at whether and how historical drug approvals could have been predicted from our current knowledge of human genetics, we can validate this approach and assess which types of genetic evidence are most likely to be useful in guiding drug discovery. Validation is important because we are often uncertain about the biological mechanisms behind genetic variants linked to disease. Most associated variants do not occur within protein-coding regions of the genome, and it is difficult to tell which of many nearby genes is contributing to disease risk. In this paper, we confirm previous correlations between genetic evidence and historical drug approvals. We find genetic evidence from severe genetic disorders and from genetic variants that alter protein sequence is more strongly associated with historical approvals. We offer statistical approaches for prioritizing new drug candidates based on whether their mechanisms are supported by human genetic evidence.

## Introduction

The cost of developing new molecular entities (NMEs) into approved therapies continues to increase with cost per launched NME ranging from $3 billion to more than $10 billion across major research based pharmaceutical companies [1]. Despite strong vetting for disease activity, only 5-10% of candidate NMEs in early stage clinical trials are eventually approved and this probability of approval has a direct relationship to total cost per approved drug [1, 2]. Thus, to maintain a sustainable drug development process, there is a critical need to increase the number of successful NMEs, while reducing the number of failures.

Analyzing historic data of the progress of drug compounds through the drug development pipeline, Nelson et al. 2015 [3] concluded pipeline drug targets with human genetic evidence of disease association are twice as likely to lead to approved drugs. The specific claim of doubled approval probability, if true, could lead to fewer failed clinical programs thereby lowering drug development costs. Indeed, using the estimated impact of genetics from Nelson et al. [3], increasing the fraction of NMEs in development with genetic support from the current value of 15% to 50% is predicted to decrease the direct R&D cost per launched drug by 22 ± 13% [4].

Several recent successes have corroborated the power of leveraging genetic data to predict the success of a new drug targets [5]. For example, the gain of function mutations in *PCSK9* [6–9], which cause familial hypercholesterolemia and coronary artery disease led to to the launch of evolocumab (Amgen) and alirocumab (Regeneron). How widely the pharmaceutical industry can expect genetics and genomics to yield increased success rates beyond these more narrowly defined examples that have unambiguous causal genes and multiple verified Mendelian mutations remains to be determined. If the association between human genetic evidence and approved drugs is genuine and continues to hold for present-day drug development, we expect better variant to gene mapping methods and more sophisticated predictive approaches will further improve our ability to prioritize drug targets. Because of the foundational nature of the Nelson et al. work [3], it is important to determine whether the reported association holds prospectively, and whether it replicates on independent data subsets not used in the original model construction.

Three years have passed since the publication by Nelson et al. and five years have passed since the data freeze used for analysis occurred [3]. The results may now be validated using drug progression events to which Nelson et al. were completely blinded at the time. Similarly, ongoing efforts in discovering disease-associated variants in increasingly large patient samples have rapidly grown the number of potential gene trait links. For example, a public central repository of genetic association studies (GWAS Catalog [10], https://www.ebi.ac.uk/gwas/) has grown by four-fold [11, 12]. Additionally, the quantity and quality of links between noncoding SNPs and genes has expanded with the development of GTEx [13]. Here we report revised estimates of the impact of genetic evidence on drug target success and extend Nelson’s observations into a model that can be deployed by other companies and academics to predict the likelihood of success of targets of interest to them.

## Results

### Identifying validation sets

**Fig 1:**
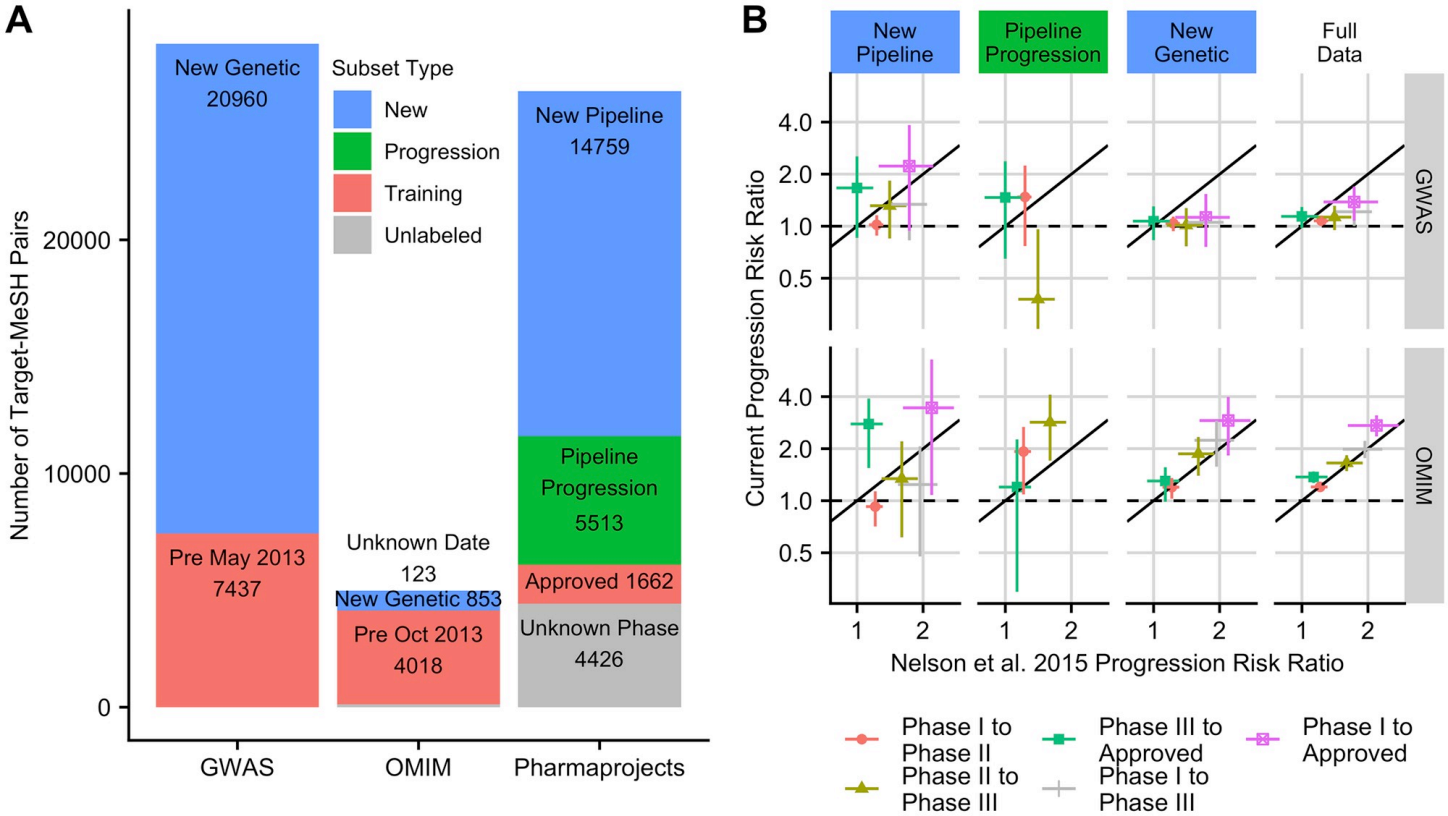
Estimated effect of evidence from human genetic studies on the probability of advancing in clinical development. A: Partitioning Pharmaprojects, OMIM, and GWAS Catalog into training data available to Nelson et al. 2015 and validation sets. We use validation set Pipeline Progression, consisting of target-indication pairs assigned a clinical phase in 2013, to determine whether gene target-indication pairs with genetic evidence were more likely to advance to the next pipeline phase from 2013-2018. Pharmaprojects target-indication pairs absent from or assigned an unknown clinical phase in the Nelson et al. dataset form the New Pipeline replication set. Pharmaprojects target-indication pairs approved prior to 2013 or with unknown phase in our dataset are not part of any replication set. B: Our estimates of the effect of genetic evidence on gene target-indication pair progression compared to values reported by Nelson et al. 2015 [3] in validation sets New Pipeline (drugs and indications > 2013, 2013 inactive drugs) New Genetic (only new genetic information > 2013) Pipeline Progression, and in the full updated dataset (Full Data). Estimates falling close to the identity line (shown in black) are consistent between the two analyses.

Nelson et al. [3] estimated a twofold increase in approval probability for Phase I drug targets with genetic evidence using drug pipeline data from Informa Pharmaprojects along with genetic data from a variety of sources, all obtained in 2013. This estimate comes from historical rather than experimental data so a direct replication is not possible. However, we can obtain updated sources of pipeline and genetic data and use the data subsets not used in the Nelson et al. study study to validate its claims. Fig 1A shows how updated pipeline (Informa Pharmaprojects [14]) and genetic association (GWAS Catalog, OMIM [15]) datasets may be split into discrete subsets, several of which were not used in the original analysis. We call these sets validation sets. In addition to genetic associations and pipeline progression events added after 2013 (New Genetic and Pipeline Progression sets), we identified a large subset of pipeline data that was available to Nelson et al., but that was excluded from analysis because Pharmaprojects reported an inactive status, most commonly “No Development Reported”. Instead of directly using Pharmaprojects development status, we use other fields in the database to label drugs with a latest historical development phase (see Methods, S2 Text), enabling us to use 83% of this data in our analysis.

Following Nelson, we aggregate data at the level of gene target-indication pair, the unit on which genetic evidence is computed. In total, we mapped 21934 gene target-indication pairs to a highest pipeline phase, in contrast to 8853 pairs labelled with a known phase in the Nelson et al. analysis. 5513 pairs could be tested for progression to a more advanced clinical phase since 2013, and 14759 pairs either absent or inactive in the 2013 data set could now be assigned a highest historical pipeline phase. Two validation sets (New Pipeline, and new GWAS associations) are larger than the original datasets used in Nelson et al. giving us sufficient power to test predictions.

Our replication analysis occurred in three steps. In the first step, we took labels of genetic evidence directly from Nelson et al. 2015 and tested how these labels predict pipeline outcomes in the New Pipeline and Pipeline Progression validation sets. Second, we repeated the analysis using both updated pipeline data and updated genetic association datasets and determined whether genetic evidence labels constructed from associations reported after 2013 are positively associated with historical progression. This analysis uses the New Genetic validation set, defined as GWAS data added after May 2013 and OMIM data added after October 2013. Third, we determine whether genetic labels constructed from the full set of updated GWAS and OMIM genetic associations are linked to improved pipeline outcomes over the entire updated Pharmaprojects dataset (See Methods for more details). We refer to this analysis as Full Data.

### Estimated effect of genetic evidence on validation sets

Of the many results from the original Nelson et al. publication, we focus on determining whether the probability of progressing along the development pipeline is greater for gene target-indication pairs with genetic evidence as this most directly impacts business decision-making (S8, S9, S11 and S12 Figs show replication of other results). A gene target-indication pair is said to have *genetic evidence* if there is human genetic evidence of association between the gene target and a trait sufficiently similar to the indication, as measured by semantic similarity in the MeSH vocabulary (see Methods and S4 Text). Fig 1B shows estimates and 95% confidence intervals for the ratio of the probability of progression for gene target-indication pairs with and without genetic evidence computed on the three validation sets and the full set of new data each plotted against values computed from Nelson et al. supplementary tables.

Across all three validation sets (Pipeline Progression, New Genetic, and New Pipeline), we consistently see a marked difference between the effect of genetic evidence derived from the OMIM database and genetic evidence derived from the GWAS Catalog. Estimated effects of OMIM genetic evidence are comparable to or greater than previously reported values [3], except for progressions from Phase I to Phase II, which are lower using new data. Notably, we see a positive and significant effect of OMIM genetic evidence on the probability of progression from Phase II to Phase III since 2013 (Pipeline Progression validation set). With the exception of progressing from Phase III to Approval, estimated effects from GWAS Catalog-derived genetic evidence are consistently lower than the originally reported values. Our estimated effects of GWAS genetic evidence in the New Genetic validation set are often significantly lower than the originally reported values. In validation sets, all estimates of the effect of GWAS evidence overlap one (no effect), except in the Pipeline Progression validation set, where we estimate a negative effect of GWAS evidence on Phase II to III progression (Fig 1B).

In both GWAS and OMIM datasets, our estimates of the effect of genetic evidence on Phase I to II progression probabilities are lower than originally reported, and confidence intervals sometimes exclude original estimates. With some exceptions (e.g. oncology studies), Phase I trials assess safety in healthy volunteers, not efficacy, so their success may be less closely linked to human genetic evidence for target involvement in disease. Validation sets may also differ systematically from the 2013 training data. For example, it is possible that there are systematic differences in the types of associations discovered before and after 2013 (New Genetic validation set). Later associations may be biased towards those with smaller effect sizes or rarer variants only detectable in larger cohorts, and could also be less predictive of drug efficacy. Using the complete updated dataset (Full Data), including all Pharmaprojects drugs and pre and post 2013 genetic associations, we find the estimated effect of GWAS genetic evidence on Phase I to Approval is still significantly positive, and the effect of OMIM genetic evidence is greater than originally reported.

### Statistical modeling of genetic effect on drug approval

The effect of GWAS genetic evidence on approval was considerably reduced and lacked statistical significance in the New Genetic dataset. In reanalyzing the original data, we found the estimated effect of GWAS genetic evidence was highly sensitive to the choice of trait-indication similarity cutoff used to determine whether or not a drug target had a genetic association (S3 Fig). Learning from this analysis, we sought to build a model relating genetic evidence to the probability of drug approval in the full dataset.

We fit multivariate logistic regression models predicting target-indication pair approval using several independent variables. The first was a measure of (continuous) genetic evidence, defined as the maximum semantic similarity to the indication across all traits linked to the drug target through human genetic evidence. The remaining independent variables are target and indication-level properties that could confound the relationship between genetic evidence and approval. Previous work has shown that approved drug targets tend to be more conserved than genes linked to GWAS associations [16], so we included residual variant intolerance score (RVIS) [17], measuring the amount of common functional variation in each gene relative to the amount of neutral variation, as a predictor. We also included the amount of time each target is known to have been under development as a predictor, with the rationale that if accumulating genetic evidence informs drug development, targets supported by genetic evidence might be newer on average. Finally, we included gene ontology (GO) terms and high level MeSH terms for each indication as predictors to control for known differences [18, 19] in approval probability among indication and target classes.

**Fig 2:**
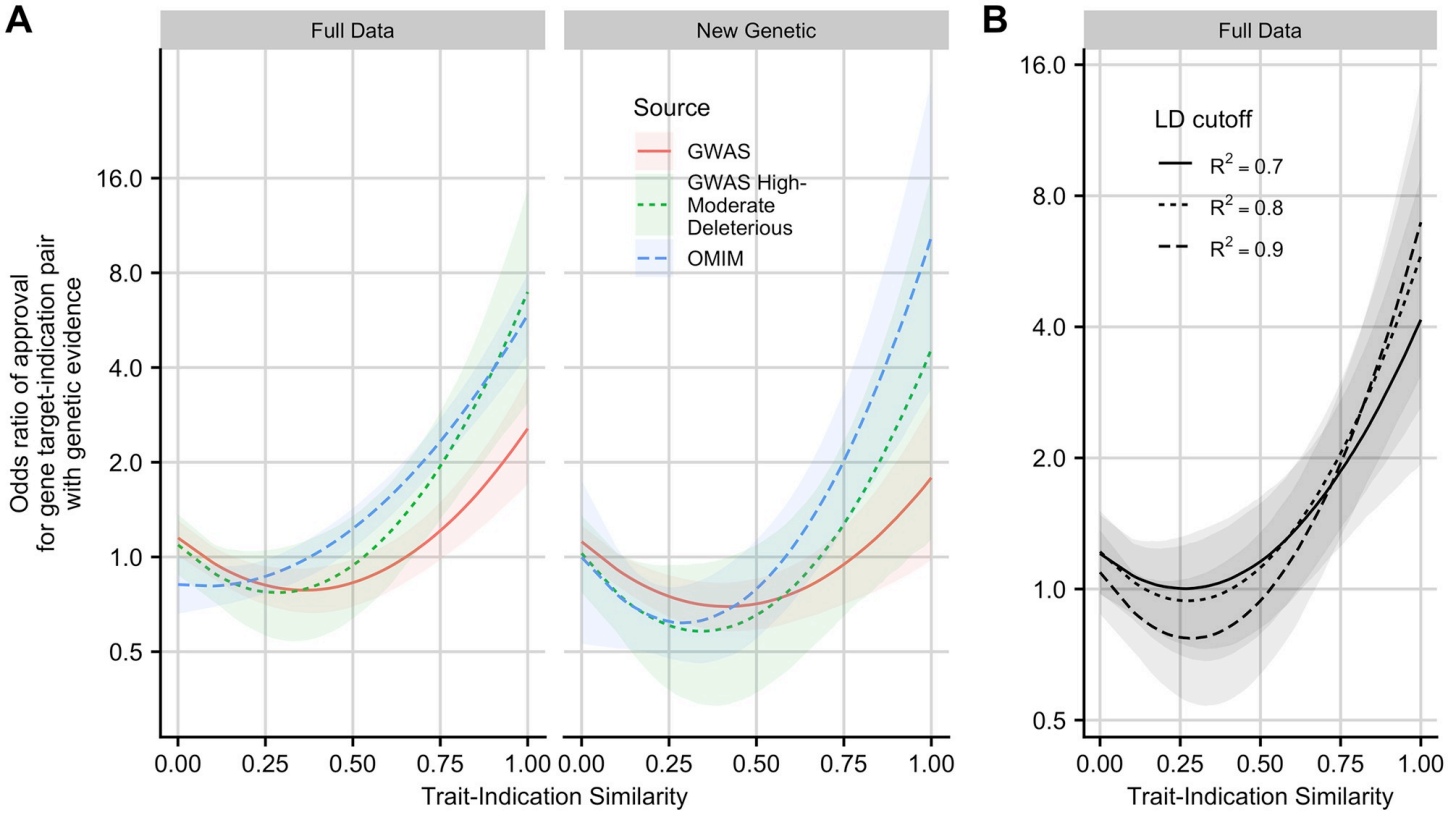
Estimated odds ratio of gene target-indication pair attaining approval, as a function of similarity between drug indication and the most similar trait associated with the target. A: Left: All genetic associations. Right: Only genetic associations reported after 2013 download. B: Effect of LD expansion threshold *R*^2^ on the estimated approval odds ratio of a drug gene target-indication pair supported by a GWAS high-moderate deleterious variant. Posterior median and pointwise 95% credible interval from Bayesian logistic regression.

Under this model, approval is positively associated with trait similarity for supporting GWAS and OMIM associations, with 95% credible intervals excluding zero (Fig 2A). When associated traits are sufficiently similar (for GWAS, roughly the similarity between Stomach Neoplasms and Colorectal Neoplasms), gene target-indication pairs with GWAS or OMIM associations are more likely to be approved. Evaluation of the data also revealed when there is a genetic association for a dissimilar disease, they are less likely to be approved than gene target-indication pairs with no known genetic association. This negative association is a novel finding.

GWAS genetic evidence has a smaller positive effect on approval than does OMIM genetic evidence, and we only find a small beneficial effect of GWAS genetic evidence in the New Genetic validation set. One possible explanation is that most GWAS associations are to noncoding variants, and determining function from these associations will require more advanced methodology [20]. Indeed, when we only consider GWAS Catalog SNPs in high LD (*R*^2^ ≥ 0.9) to a missense variant or other variant predicted to be moderately or highly deleterious [21], the estimated effect of GWAS genetic evidence on drug target approval approaches that of OMIM. Moreover, for missense variants, we see a larger estimated effect of genetic evidence when using a more stringent LD cutoff to the lead SNP (Fig 2B).

## Discussion

Pharmaceutical companies are investing in the creation and analysis of genomics data in the hope of improving target selection and decreasing failures due to lack of efficacy [22] or adverse effects [23]. Previous work by Nelson et al. 2015 [3] supported this investment, showing gene target-indication pairs with genetic evidence are approximately twice as likely to progress from Phase I to approval. This quantitative estimate is the product of many decisions, for example how to identify similar traits in genomics and pipeline databases, that, although reasonable, could have been made differently. Additionally, the results were based on a large historical set of approved drugs and might not hold for present-day target selection. This motivated us to replicate the analysis using 5 years of data that has accumulated since their data freeze in 2013.

In the replication study, we recovered a robust association between OMIM genetic evidence and drug approval of a similar or greater magnitude to that originally reported [3] across several independent test sets. GWAS genetic evidence also is generally positively associated with progressing in clinical development, but the magnitude of the association is smaller and not clearly different from zero in any independent replication set. One possible reason is that recently reported GWAS variants have smaller reported effect sizes. We find evidence for this claim, but do not detect an effect of GWAS evidence effect size on approval (S13 Fig, S22 Table). There appears to be some confounding due to GWAS genes having different properties than approved drug targets. When this is controlled for using logistic regression, GWAS-supported target-indication pairs are more likely to be approved than those without a GWAS-linked gene target. This highlights the need for predictive models including target properties, work that is beginning to emerge [24].

The OMIM database provides expert-curated gene-trait links, bypassing the need to assign noncoding SNPs to genes, a major source of uncertainty for present GWAS methods. Better methods for linking GWAS SNPs to causal genes may improve performance, supported by the fact that we found strong and statistically significant positive associations between GWAS genetic evidence and drug success when considering only the highest confidence SNP-gene links, characterized as having a leading SNP with *R*^2^ ≥ 0.9 to a variant predicted to be highly or moderately deleterious. However, OMIM’s focus on Mendelian phenotypes also means genetic variants will be higher effect size than those for quantitative traits or conditions prominent in the GWAS Catalog, which is unlikely to be addressed by improved computational methods.

Because OMIM is a manually curated database, it is possible that known drug mechanisms influence OMIM entries, creating a positive association between OMIM genetic evidence and approval. However, we observe a positive effect of OMIM genetic associations reported by Nelson et al. 2015 on progression events occurring after data were collected for that paper, which is inconsistent with this reverse causal hypothesis. It is also possible these progression events are not truly independent of pre-2013 approvals, because they may represent approval for an indication similar to the original indication. However, the positive effect of OMIM genetic evidence on 2013-18 progression remains significant when targets with pre-2013 approvals for similar indications are excluded (S11 and S12 Tables). Another possibility is that the success of OMIM is due to treatments such as protein replacement therapies for monogenic diseases, which may have higher success rates as a whole [25]. However, we still find a large positive effect of OMIM genetic evidence when we exclude hereditary diseases and MeSH terms mapped to OMIM phenotypes from the analysis (S21 Table, S27 Fig). We conclude the predictive effect of OMIM genetic evidence is not a statistical artifact, and is more likely to reflect the value of well-defined disease biology to drug development.

Due to the MeSH ontology structure, current methods require manual similarity assignments to recognize relationships between most quantitative traits and diseases. The high sensitivity of key results to MeSH similarity motivates treating similarity as a continuous variable and suggests improvements to its quantification. While expert curation can be advantageous in identifying closely related traits, it also leaves more room for human input to bias the analysis outcome. To assess this we removed automatically assigned similarities. Positive associations between GWAS genetic evidence and approval remain, though in some cases are greatly reduced in magnitude (S19 and S26 Figs) (OMIM is minimally impacted as it contains few quantitative traits). We expect improved methods automatically identifying similar phenotypes to drug indications will expand our ability to use genomics data in predictive models.

Our results highlight the importance of similarity between associated trait and drug indication in determining which gene target-indication pairs are likely to lead to approved drugs. Our finding that genetic associations for highly dissimilar traits reduce the probability of approval is new and could be of significance once the reason is better understood. A possible explanation is an increased incidence of side effects due to involvement in unrelated disease mechanisms. It suggests that when target disease links are known, genetic data can improve the drug development process through improved indication selection.

Our analysis of the last five years of drug development data validates the results of Nelson et al. and indicates that the positive association between genetic evidence and drug success is not just a historical phenomenon. Using logistic regression to control for target and indication level properties, and quantifying genetic evidence on a continuous scale, we also demonstrated that associations to disparate phenotypes is a negative predictor of approval. With these algorithmic developments, we have built a Shiny [26] app that others can use to evaluate target-indication pairs of interest. As mechanistic understanding of genetic associations increases, our data suggests the reliability of genetic predictions of drug targets will continue to improve. In closing, public and private investments into genomics for the purpose of improving the fraction of successful drug targets appears to be well warranted.

## Materials and methods

### Data sources

#### Pipeline data

Data on drug gene targets, indications, latest development phase, and approvals by country were collected from the Informa Pharmaprojects database (accessed January 25, 2018) [14]. For each drug, Pharmaprojects provides country-level, indication-level, and global development status. The latter is the latest development status across indications for any country. A drug was considered US/EU approved for an indication if it was approved in the US or EU and approved for that indication (so if a drug is US/EU approved for one but not all of its approved indications, we will incorrectly assign some approvals). We infer this was also the approach of Nelson et al., as they mention no source other than Pharmaprojects for drug approval data and Pharmaprojects does not provide drug-indication-country level approval data.

To calculate phase-specific progression probabilities by genetic evidence, we must assign a latest historical development phase to Pharmaprojects drug-indication pairs that are not in active development using other database fields. Country status gives the latest phase for single-indication and preclinical drugs. Other drug-indication phases are determined through assessing the presence or absence of key events and clinical details matching the trial phase and the disease name. Clinical details were only used when other sources were unavailable because this field may contain information about planned or anticipated trials. Details are provided in S2 Text.

Pharmaprojects gene targets were mapped from Entrez to ensembl IDs. Drugs with non-human and xMHC targets were excluded (following the original analysis) as were a small number of drugs with non protein coding targets.

#### Genetic data

Genetic association data was obtained from the GWAS Catalog [10] downloaded 2018-11-18. OMIM data was downloaded from [15] on 2018-11-18. GWAS Catalog associations with reported *p*-value greater than 10^−8^ were excluded, following the original analysis, as were OMIM provisional associations, drug response associations, and somatic variant associations.

OMIM reports gene-trait links, but the GWAS Catalog reports SNP-trait links which must be converted to gene-trait links via SNP-gene links. Although methods for creating SNP-gene links have since advanced [20], we closely follow the approach of [3] with updated data sources to reduce our degrees of freedom for overfitting to new data and to make our new estimates of the effect of genetic evidence comparable to the original estimates. Our gene-trait mapping procedure attempts to replicate that used by Nelson et al. with updated data sources. An LD expansion of GWAS Catalog reported variants was performed using an LD threshold of 0.5 in the 1000 Genomes Phase 3 EUR super population [27]. A distance-based gene-trait association was established when an LD SNP was within 5000 b.p. of the gene in hg38 as annotated by SNPEff [21]. An eQTL-based gene-trait link was established when an LD SNP was reported associated with a gene with nominal *p*-value less than 10^−6^ in any GTEx tissue [13]. Using a cutoff of 10^−12^ makes little difference to results (S20 Table). A DHS-based gene-trait link was established when an LD SNP was located in a DNAse I hypersensitivity site correlated with gene expression with one-sided permutation *p*-value 1.000 (from 1000 replicates) [28]. All linked genes were mapped to Ensembl IDs, and links to genes not annotated as protein coding by Ensembl were removed from the dataset. Additional details are available in S3 Text.

### Genetic evidence

#### Trait-indication similarities

Pharmaprojects indications and GWAS Catalog and OMIM traits were mapped to MeSH headings to link traits and indications by a common vocabulary. We mapped as many terms as possible automatically by string matching to MeSH terms and their synonyms, and the remainder were manually assigned to the most specific MeSH heading encompassing the term. The MeSH vocabulary consists of MeSH headings, which are organized in a hierarchy, and supplementary concepts, which are not. We did not map to MeSH supplementary concepts as the lack of structure means we cannot compute similarities between these concepts and other terms. However, each supplementary concept is assigned one or more mapped headings, and so terms matching a supplementary concept were assigned to the mapped heading. This set of MeSH term mappings was used in the full replication with new genetic data sources.

When testing predictions from the 2013 genetic association data, it was important that MeSH headings mapped to Pharmaprojects indications be consistent with the original analysis by Nelson et al. in order to correctly identify common pairs between datasets for which progression can be tested and to ensure that our New Pipeline test set contained truly novel pairs. Nelson et al. provided mappings for many Pharmaprojects indications in a supplementary dataset. Terms without provided mappings were mapped to maximize the number of Nelson et al. gene target-indication pairs also present in our dataset, subject to the mapping being biologically justifiable. Standardized mapping increased the percent of Nelson et al gene target-indication pairs present in our dataset from 88% (using our independently mapped terms) to 98%.

Resnik [29] and Lin [30] similarities between MeSH headings were computed in R in the ontologySimilarity package [31], standardized to have a maximum value of 1 for each trait, and averaged to compute a similarity between each pair of MeSH headings (S4 Text). Two traits are considered *similar* if the similarity is greater than or equal to a critical value. Our assigned similarities are not identical to those of Nelson et al. because of using different versions of MeSH (2009 versus 2017), but were correlated with those originally reported (*R*^2^ = 0.86, S17 Fig). We determined a critical value of 0.73 in our analysis corresponded to the critical value 0.7 used in the original analysis, and used this to determine similar traits in our replication study. Manually assigned similarities were taken from the supplement of [3]. Manual assignment was performed because the MeSH ontology makes few connections between diseases and closely related quantitative phenotypes, for example osteoporosis and bone density.

#### Defining genetic evidence

We formalize and extend the concept of genetic evidence used by Nelson et al. We first define a similarity function operating on two gene-trait pairs. Define function *S* from ${\left(G\times T\right)}^{2}$ to [0, 1] where G is the space of genes and T is the space of traits.

$$\begin{array}{c} S\left(\left(g_{1},t_{1}\right),\left(g_{2},t_{2}\right)\right)=\{\begin{array}{cc} S_{T}\left(t_{1},t_{2}\right) & g_{1}=g_{2}\\ 0 & \text{otherwise} \end{array} \end{array}$$

where $S_{T}:T\times T\to \left[0,1\right]$ is a trait similarity function (in the Nelson et al analysis and here, computed from Resnik and Lin similarities). Let A be a set of gene-trait pairs with elements in G×T obtained from genetic data sources (for example, when analyzing the effect of OMIM genetic evidence A is the set of gene-trait pairs in OMIM). Genetic evidence according to Nelson et al. 2015 is a function *E*_*D*_ from G×T to {0,1}

$$\begin{array}{c} E_{D}\left(g,t\right)=\{\begin{array}{cc} 1 & \text{max}\{S\left(\left(g,t\right),\left(g_{a},t_{a}\right)\right):\left(g_{a},t_{a}\right)\in A\}\geq 0.7\\ 0 & \text{otherwise} \end{array} \end{array}$$

However, trait similarity is a real number in [0, 1], so we can define another genetic evidence function *E*_*C*_ from G×T to [0, 1]

$$\begin{array}{c} E_{C}\left(g,t\right)=\max\left\{S\left(\left(g,t\right),\left(g_{a},t_{a}\right)\right):\left(g_{a},t_{a}\right)\in A\right\} \end{array}$$

*E*_*D*_(*g*, *t*) = 1 if and only *E*_*C*_(*g*, *t*) ≥ 0.7.

### Statistical analysis

All statistical analyses are performed on pipeline data collapsed to one row per gene target-indication pair, as this is the unit on which genetic evidence is measured. The latest phase of a gene target-indication pair (*g*, *i*) is the most advanced pipeline phase attained by any drug with target *g* for indication *i*. Of several results of [3], we are most interested in the claim that target-indication pairs supported by genetic evidence are more likely to advance than those without. In the first part of the analysis, we quantify this association as a risk ratio, attempting to replicate the original Nelson et al analysis as closely as possible. Second, we introduce a logistic regression model for the relationship between approval and genetic evidence, adjusting for covariates at the target and indication levels.

#### Two-by-two tables

Let **D** be a vector of gene target-indication-phase triplets with elements (*g*_*i*_, *t*_*i*_, *h*_*i*_), *i* = 1, …, *n*. *H*_*i*_ ∈ {0, …, 4} is an ordered categorical variable giving the latest phase each gene target-indication pair has achieved (0 = Preclinical, 1 = Phase I, 2 = Phase II, 3 = Phase III, and 4 = US/EU Approved).

Risk ratios for progressing from Phase *x* to Phase *y*, *x* > *y* were computed as

$$\begin{array}{c} \frac{\text{P(Success}|\text{Genetic}\;\text{Evidence})}{\text{P(Success}|\text{No}\;\text{Genetic}\;\text{Evidence})}=\frac{N_{g,x}/N_{g,y}}{N_{g^{\prime },x}/N_{g^{\prime },y}} \end{array}$$

where $N_{g,x}=\sum_{i=1}^{n}E_{D}\left(g_{i},t_{i}\right)I\left(h_{i}\geq x\right)$ is the number of gene target-indication pairs in Phase *x* or later with genetic evidence and $N_{g^{\prime },x}=\sum_{i=1}^{n}\left(1-E_{D}\left(g_{i},t_{i}\right)\right)I\left(h_{i}\geq x\right)$ is the number of gene target-indication pairs in Phase *x* or later without genetic evidence. We required at least 5 reported genetic associations for similar traits. Phase progression probability calculations usually exclude in progress development [18] but here we include them for consistency with Nelson et al. Confidence intervals were computed using the riskratio.boot function in the epitools R package [32]. We ensured consistency of this approach with that of Nelson et al. by verifying our code could reproduce their results from supplemental materials (S1 Text). Drugs approved only outside the US and EU and drugs with unknown latest phase were excluded from this analysis.

#### Bayesian logistic regression

Let *i* index gene target-indication pairs (*g*_*i*_, *t*_*i*_), *i* = 1, …, *N*. Let *y*_*i*_ ∈ {0, 1} be 1 if pair *i* is found in at least one US/EU approved drug and 0 otherwise. Let *X* be an *N* × *d* design matrix where *d* is the number of non-genetic predictors with *i*^*th*^ row $x_{i}^{\prime }$.

$$\begin{array}{c} y_{i}\overset{\text{indep}}{∼}\text{Bernoulli}\left({\text{logit}}^{-1}\left(\alpha +\eta_{i}+x_{i}^{\prime }\beta \right)\right)\;i=1,\ldots ,N \end{array}$$

where

$$\eta_{i}=\{\begin{array}{ll} \sum_{j=0}^{p}\gamma_{j}E_{C}{\left(g_{i},t_{i}\right)}^{j} & \text{there}\;\text{exists}\;\left(g,t\right)\in A\;\text{such}\;\text{that}\;g=g_{i}\\ 0 & \text{otherwise} \end{array}$$

Our choice of *p* = 2 is supported by WAIC [33] [34]. Predictors in **X** were top-level MeSH category, target class, estimated time the target has been under development, and RVIS score [17]. Details are provided in S5 Text. Priors were

$$\begin{array}{c} \alpha ∼N(\mu_{a},\sigma_{a}^{2}); \end{array}$$

$$\begin{array}{c} \beta_{j}\overset{\text{iid}}{∼}N\left(0,\sigma_{b}^{2}\right)\;j=1,\ldots ,d \end{array}$$

$$\begin{array}{c} \gamma_{j}\overset{\text{iid}}{∼}N\left(0,\sigma_{g}^{2}\right)\;k=0,1,2 \end{array}$$

All models were fit in Stan [35] using four chains with default initialization and run settings.

Prior parameters *μ*_*a*_ = -2.2, *σ*_*a*_ = 0.75 was chosen to reflect prior knowledge that approximately 10% of Phase I compounds become approved [18] and prior standard deviations *σ*_*b*_ = 2, and *σ*_*g*_ = 2 were chosen prior belief that observed effect sizes should be moderate. Note *α*, for which we have chosen a nonzero mean prior, controls the baseline approval probability, not the effect of genetic evidence. Continuous covariates in **X** were standardized to have mean 0 and standard deviation 1 as was *E*_*C*_.

In this analysis we depart from the original Nelson et al. approach and exclude all drugs assigned an active development phase by Pharmaprojects, as it is unknown whether these development programs will ultimately lead to approval. This decision is consistent with other work estimating clinical success probabilities [18] [24]. We include unapproved drugs with unknown latest historical phase. A total of 20292 gene target-indication pairs were associated with at least one US/EU approved or inactive drug and included in the analysis.

### Code availability

Code and data tables required to reproduce the main text figures are provided on Github (https://github.com/AbbVie-ComputationalGenomics/genetic-evidence-approval). The git repository also contains instructions for running a Shiny app displaying model predictions.

## Supporting information

S1 Text Reanalysis of Nelson et al. supplementary datasets. (PDF)

S2 Text Updating pipeline data: Supplementary methods and results. (PDF)

S3 Text Updated genetic dataset: Supplementary methods and results. (PDF)

S4 Text Trait-indication similarity: Supplementary methods and results. (PDF)

S5 Text Modeling drug success probability: Supplementary methods and results. (PDF)

S1 Fig Enrichment of approved drug targets among genes with human genetic evidence recreated from Nelson et al. 2015 supplementary tables. Replication of Figure 2N from Nelson et al. supplementary datasets. Figure shows the enrichment of approved drug targets among genes with human genetic associations.
(PDF)

S2 Fig Enrichment of approved drug targets among genes with human genetic evidence recreated from Nelson et al. 2015 supplementary tables. Replication of Figure 3N from Nelson et al. supplementary datasets. Figure shows the proportion of gene target-indication pairs with genetic associations for similar traits by pipeline phase and association source.
(PDF)

S3 Fig Sensitivity of the effect of genetic evidence to trait similarity. Sensitivity of risk ratios p(approved | genetic support)/p(approved | no genetic support) and 95% confidence limits to choice of MeSH similarity cutoff. Nelson et al. value 0.7 shown in red. Results are computed from Nelson et al supplementary datasets.
(PDF)

S4 Fig Sensitivity of the effect of genetic evidence to minimum number of genetic associations. Sensitivity of risk ratios p(approved | genetic support)/p(approved | no genetic support) and 95% confidence limits to choice of minimum number of associations parameter. Nelson et al. value of 5 shown in red. Results are computed from Nelson et al supplementary datasets.
(PDF)

S5 Fig Concordance between known Pharmaprojects statuses and assigned phases. Assigned latest phase compared to Pharmaprojects status (unknown Pharmaprojects status categories such as No Development Reported and Suspended are combined) at the global and indication level.
(PDF)

S6 Fig Latest change date to Pharmaprojects entry by development status. Latest change date for Pharmaprojects drugs by development status. In panel Pharmaprojects Global Status, statuses come from the Pharmaprojects global status field. In panel Global Latest Phase, statuses are the latest global development phase assigned in this document.
(PDF)

S7 Fig First event date in Pharmaprojects by development status. Earliest date in the Pharmaprojects event history by status. In panel Pharmaprojects Global Status, statuses come from the Pharmaprojects global status field. In panel Global Latest Phase, statuses are the latest global development phase assigned in this document. Note 6% of compounds do not have any entries in their event history and are omitted.
(PDF)

S8 Fig Enrichment of approved drug targets among genes with human genetic evidence using updated pipeline data and genetic associations from Nelson et al. 2015. Replication of Figure 2N from Nelson et al. supplementary genetic association dataset and updated pipeline data. Figure shows the enrichment of approved drug targets among genes with human genetic associations.
(PDF)

S9 Fig Percent of targets with human genetic evidence by pipeline phase using updated pipeline data and genetic associations from Nelson et al. 2015. Replication of Figure 3N from Nelson et al. supplementary genetic association dataset and updated pipeline data. Figure shows the proportion of gene target-indication pairs with genetic associations for similar traits by pipeline phase and association source.
(PDF)

S10 Fig Concordance between dates added to GWAS Catalog and appearance in Nelson et al. 2015. Date SNP associations appearing in the reanalysis were added to the GWAS Catalog by whether or not Nelson et al. reported the association. Line shows the date of the GWASdb version used in Nelson et al. 2013.
(PDF)

S11 Fig Enrichment of approved drug targets among genes with human genetic evidence using updated pipeline and genetic association data. Replication of Figure 2N from updated GWAS Catalog genetic association dataset and updated pipeline data. Figure shows the enrichment of approved drug targets among genes with human genetic associations.
(PDF)

S12 Fig Percent of targets with human genetic evidence by pipeline phase using updated pipeline and genetic association data. Replication of Figure 3Nb from updated GWAS Catalog genetic association dataset and updated pipeline data. Figure shows the proportion of gene target-indication pairs with genetic associations for similar traits by pipeline phase and association source.
(PDF)

S13 Fig Decline in effect size of newly reported GWAS associations through time. Median odds ratio for new reported case-control SNP-trait associations through time. A new reported SNP-trait association is one appearing in the GWAS Catalog for the first time, in contrast to a replicate of a previous association.
(PDF)

S14 Fig Agreement between estimated effect size in earliest GWAS studies and later replications. Relationship between effect size in the first study and effect size in the study with the largest sample size for GWAS Catalog case-control studies for associations that have been replicated.
(PDF)

S15 Fig Decline in effect size of newly reported GWAS associations through time, using later estimates from larger studies. Median odds ratio for new reported case-control SNP-trait associations through time. A new reported SNP-trait association is one appearing in the GWAS Catalog for the first time, in contrast to a replicate of a previous association. Only studies with a later replicate are considered and the effect size reported is from the largest replicate. Only years with at least 20 replicated studies are shown.
(PDF)

S16 Fig Computing information content from MeSH. A portion of the MeSH vocabulary used to illustrate semantic similarity. Information contents from the number of descendants (computed from the entire ontology, not just the portion shown here) are given in parentheses.
(PDF)

S17 Fig Relationship between semantic similarities computed in this study and computed by Nelson et al. 2015. Nelson et al. semantic similarity versus semantic similarity (average of Lin and Resnik similarities) computed in this analysis. Black points show a random sample of 50,000 trait pairs for which both similarities were available. Blue line shows smoothed relationship estimated using all possible similarity pairs. Dashed red lines show old and new cutoff values.
(PDF)

S18 Fig Main text figure 1b, using similarity cutoff 0.7 instead of 0.73. Estimated effect of genetic evidence on pipeline progression. Main text Figure 1b computed with similarity cutoff 0.7. This cutoff was originally used in Table 1N, but our main text figure uses similarity cutoff 0.73 because of systematic differences in computed similarities.
(PDF)

S19 Fig Effect of manually assigned similarities on estimated effect of genetic evidence. Risk ratio of Phase I to approval progression for gene target-indication pairs with and without genetic evidence for different values of the MeSH similarity cutoff split by whether MeSH similarity is automatically assigned, manually assigned, or using both automatic and manually assigned similarities (default). Full Data and New Genetic are as described in main text. 2013: computed from supplementary tables.
(PDF)

S20 Fig Target approval probability and GWAS associations by indication. Proportion of gene target-indication pairs approved against proportion of gene target-indication pairs with a GWAS Catalog associated target by indication class. Larger text designates indication classes with more gene target-indication pairs. Only classes with 50 or more pairs are shown.
(PDF)

S21 Fig Target approval probability and GWAS associations by target class. Proportion of gene target-indication pairs approved against proportion of gene target-indication pairs with a GWAS Catalog associated target by target class (GO terms). Larger text designates target classes with more gene target-indication pairs. Only classes with 50 or more pairs are shown.
(PDF)

S22 Fig Target approval by RVIS score. Proportion of approved gene target-indication pairs binned by target RVIS score percentile.
(PDF)

S23 Fig Target approval by development time. Proportion of approved gene target-indication pairs binned by date first drug with target added to Pharmaprojects.
(PDF)

S24 Fig Coefficient estimates. Coefficient estimates for the effect of genetically associated trait similarity on gene target-indication pair approval using different predictor subsets. See Methods for details of coefficient definitions. Note coefficients apply to centered and scaled trait-indication similarity so that the intercept for GWAS genetic evidence is the effect of genetic evidence at the mean value of GWAS trait similarity. GO = Gene Ontology terms, RVIS = RVIS score, Time = Time since target entered development.
(PDF)

S25 Fig Effect of including predictors on genetic evidence effect estimates from logistic regression. Effect of including predictors on the estimated relationship between indication-trait similarity and approval. Estimated odds ratio of gene target-indication pair attaining approval, as a function of similarity between drug indication and the most similar trait associated with the target. Posterior median and pointwise 95% credible interval from Bayesian logistic regression.
(PDF)

S26 Fig Effect of manually assigned similarities on genetic evidence effect estimates from logistic regression. Effect of excluding manually assigned trait similarities on estimated relationship between GWAS genetic support and approval. Estimated odds ratio of gene target-indication pair attaining approval, as a function of similarity between drug indication and the most similar trait associated with the target. The two colors correspond to estimates when using and when excluding manually assigned similarities. Posterior median and pointwise 95% credible interval from Bayesian logistic regression.
(PDF)

S27 Fig Effect of excluding congenital disease indications on genetic evidence effect estimates from logistic regression. Estimated effect of OMIM genetic evidence on target-indication pair approval, excluding congenital diseases and indications with mapped MeSH term also mapped to an OMIM indication. Posterior median and 95% credible intervals based on 8907 target-indication pairs, compared to results from the full data with 20292 target-indication pairs.
(PDF)

S1 Table Schematic of two-by-two table used in odds ratio calculation. *n*_*assoc*_ is the number of protein coding genes with genetic associations, *n*_*approved*_ is the number of protein coding genes linked, *n*_*aa*_ is computed as the number of protein coding genes that are both the targets of approved drugs and have reported trait associations.
(PDF)

S2 Table Association between genetic evidence and clinical progression recreated from Nelson et al. 2015 supplementary tables. Replication of Table 1N (association between genetic evidence and historical progression) from Nelson et al. supplementary datasets. Risk ratio p(approved | genetic support)/p(approved | no genetic support) and bootstrap 95% confidence intervals.
(PDF)

S3 Table Coverage of Pharmaprojects development status information. Proportion of drug-indication pairs (Indication) or drugs (Global) having development status information available from each source. Event = Pharmaprojects event history, Info = Pharmaprojects clinical information fields, Country = Pharmaprojects country status, Global = Indication status inferred from global status.
(PDF)

S4 Table Concordance of Pharmaprojects development status inferred from different fields. Agreement between Pharmaprojects status (Type = Global or Indication) and latest phase using each evidence source when both are assigned a known development status. Columns less, greater, and equal are the proportion of times in which the source implicates a latest pipeline phase less advanced than, more advanced than, or equal to that reported by Pharmaprojects. Arranged in order of decreasing agreement.
(PDF)

S5 Table Association between genetic evidence and clinical progression using updated pipeline data and genetic associations from Nelson et al. 2015. Replication of Table 1N (association between genetic evidence and historical progression) from Nelson et al. supplementary genetic association dataset and updated pipeline data. Risk ratio p(approved | genetic support)/p(approved | no genetic support) and bootstrap 95% confidence intervals.
(PDF)

S6 Table Progression 2013-2018 by phase. Risk ratio of pipeline progression from 2013 to 2018 by presence or absence of supporting genetic evidence and 2013 phase. Risk ratio and 95% confidence intervals. Last column gives the total number of gene target-indication pairs labeled with that phase in 2013 and the total number of gene target-indication pairs that progressed in development.
(PDF)

S7 Table Approval or progression 2013-2018. Risk ratio of pipeline progression from 2013 to 2018 by presence or absence of supporting genetic evidence. Risk ratio and 95% confidence intervals. Last column gives the total number of 2013 gene target-indication pairs included in the analysis and the total number of drugs that progressed in development. This table shows the risk ratio of progression to a higher phase or to approval from any starting phase.
(PDF)

S8 Table Association between genetic evidence and clinical progression in New Pipeline test set. Replication of Table 1N (association between genetic evidence and historical progression) from Nelson et al. 2015 supplementary genetic association dataset and updated pipeline data, using only gene target-indication pairs not used in the original analysis either due to not being in the table of gene target-indication pairs or having an inactive development status. Risk ratio p(approved | genetic support)/p(approved | no genetic support) and bootstrap 95% confidence intervals.
(PDF)

S9 Table Association between genetic evidence and clinical progression using only new target-indication pairs. Replication of Table 1N (association between genetic evidence and historical progression) from Nelson et al. 2015 supplementary genetic association dataset and updated pipeline data, using only gene target-indication pairs not used in the original analysis due to not being in the table of gene target-indication pairs. Risk ratio p(approved | genetic support)/p(approved | no genetic support) and bootstrap 95% confidence intervals.
(PDF)

S10 Table Association between genetic evidence and clinical progression using only 2013 inactive target-indication pairs. Replication of Table 1N (association between genetic evidence and historical progression) from Nelson et al. 2015 supplementary genetic association dataset and updated pipeline data, using only gene target-indication pairs not used in the original analysis due to being assigned an inactive status. Risk ratio p(approved | genetic support)/p(approved | no genetic support) and bootstrap 95% confidence intervals.
(PDF)

S11 Table Progression 2013-2018 excluding pairs with similar 2013 approved mechanism. Risk ratio of progression in clinical development from 2013 to 2018 by presence or absence of supporting genetic evidence. Calculations are performed on the subset of target-indication pairs without similar 2013 approved target-indication pairs, using similarity cutoff 0.73. Risk ratio and 95% confidence intervals.
(PDF)

S12 Table Progression 2013-2018 excluding pairs without previous approvals for the target. Risk ratio of progression in clinical development from 2013 to 2018 by presence or absence of supporting genetic evidence. Calculations are performed on the subset of target-indication pairs with no approved 2013 drugs for that target. Risk ratio and 95% confidence intervals.
(PDF)

S13 Table Approval by OMIM genetic evidence using MeSH supplementary concepts. Replication of Table 1N (association between genetic evidence and historical progression) from Nelson et al. supplementary genetic association dataset and updated pipeline data with all MeSH terms assigned valid headings (All OMIM). Column New OMIM shows results using only OMIM entries originally mapped to supplementary concepts (and therefore not used in Table 1N). Risk ratio p(approved | genetic support)/p(approved | no genetic support) and bootstrap 95% confidence intervals.
(PDF)

S14 Table GWAS annotation data sources. Sources used to obtain and annotate GWAS variants. Filter gives the *p*-value or other cutoff used to filter results.
(PDF)

S15 Table Overlap between Nelson et al. 2015 genetic associations and current analysis. Comparison of counts of distinct genes, traits (MeSH), and SNPs reported by Nelson et al. and those from the current analysis. Overlap is the number of items in common.
(PDF)

S16 Table Overlap between Nelson et al. 2015 genetic associations and current analysis conditional on common SNP association. Comparison of counts of distinct genes, traits (MeSH), and SNPs reported by Nelson et al. and those from the current analysis, restricted to SNP associations expected to appear in both datasets (pre-May 21, 2013 GWAS Catalog associations). Overlap is the number of items in common.
(PDF)

S17 Table Overlap between Nelson et al. 2015 genetic associations and current analysis by eQTL status. Percent of LD SNP-Gene associations reported by Nelson et al. also found in the reanalysis by whether the association was an eQTL. Conditional on LD SNP presence in both analyses.
(PDF)

S18 Table Overlap between Nelson et al. 2015 genetic associations and current analysis by evidence source. Percent of LD SNP-Gene associations found in reanalysis also reported by Nelson et al. subdivided by what evidence source(s) were used to link the LD SNP and gene. Conditional on LD SNP presence in both analyses.
(PDF)

S19 Table Association between genetic evidence and clinical progression using updated pipeline and genetic association data. Replication of Table 1N (association between genetic evidence and historical progression) from updated GWAS Catalog and OMIM genetic association dataset and updated pipeline data (Full Data). Risk ratio p(approved | genetic support)/p(approved | no genetic support) and bootstrap 95% confidence intervals.
(PDF)

S20 Table Sensitivity of association between genetic evidence and clinical progression to eQTL *p*-value cutoff. Replication of Nelson et al. Table 1 from updated GWAS Catalog and OMIM genetic association dataset and updated pipeline data, eQTL *p*-value cutoff 10^−12^ (versus 10^−6^, used in main analysis).
(PDF)

S21 Table Association between OMIM genetic evidence and pipeline progression by indication class. Effect of OMIM genetic evidence on historical pipeline progression. Risk ratio p(approved | genetic support)/p(approved | no genetic support) and bootstrap 95% confidence intervals. Pharmaprojects drugs are subdivided by indication MeSH heading: Congenital indications are those indexed under Congenital, Hereditary, and Neonatal Diseases and Abnormalities and OMIM indications are those MeSH headings that are also mapped MeSH headings to an OMIM phenotype.
(PDF)

S22 Table Association between GWAS genetic evidence and pipeline progression by case-control odds ratio. Estimated effect of GWAS genetic evidence from case-control studies on historical pipeline progression by effect size (odds ratio). Risk ratio p(approved | genetic support)/p(approved | no genetic support) and bootstrap 95% confidence intervals.
(PDF)

S23 Table Computing semantic similarity from MeSH. Similarity between different pairs of traits computed using Resnik similarities, Lin similarities, normalized Resnik similarities, and average of normalized Resnik and Lin similarities using number of descendants to compute information content.
(PDF)
