# Supplementary material for: Are drug targets with genetic support twice as likely to be approved? Revised estimates of the impact of genetic support for drug mechanisms on the probability of drug approval
Source: PLoS Genet. 2019 Dec 12;15(12):e1008489. doi: 10.1371/journal.pgen.1008489 (PMC6907751; doi:10.1371/journal.pgen.1008489)
Supplement: S1 Text — (PDF) [file pgen.1008489.s001.pdf]

S1 Reanalysis of Nelson et al. Supplementary Datasets

S1.1 Replication of main text figures

Figure 1N In the supplementary and main results of this paper, we recreate figures from the original Nelson et al. publication with updated data sources. Before doing this, we determined if we could replicate the figures from supplementary data from the original paper (supplementary datasets 2, 3, and 4 of [2]). Figures and tables from the original publication will be referred to with the suffix N, i.e. Figure 1N. Figure 1N [2] gives the total number of MeSH, genes, and gene-MeSH pairs in the Pharmaprojects database and the GWASdb separated by source. We exactly reproduce Figure 1N from supplementary tables if sources "dbGaP", "GWAS:A", "GWAS:B", "GWASCentral", "JohnsonOdonnell" and "Omim" are considered part of the GWASdb and source "OMIM" is the only source of OMIM associations. Source "Omim" appears to have been derived from the OMIM database, although it features SNP-trait links and is largely non-overlapping with reported "OMIM" associations. We elected to exclude this data source from both the GWASdb and OMIM datasets as we wished to have a clear separation between Mendelian genetic evidence and genetic evidence from GWAS.

Figure 2N Figure 2N, replicated in S1 Fig, shows enrichment of approved targets among genes with known human genetic associations. Odds ratios are computed from the 2×2 table as shown in S1 Table. The upper panel shows the odds ratio computed with respect to a population of 22,012 protein-coding genes, and the lower panel shows the same calculation with respect to the population of druggable genes, which we obtained from the drug-gene interaction database [1, 4]. RVIS scores for each gene were downloaded from the supplemental material of [3].

Figure 3N and Table 1N Figure 3N, replicated in S2 Fig, shows the proportion of gene target-indication pairs with genetic evidence by phase and by indication. Table 1N, replicated in S2 Table, shows the risk ratio of pipeline progression for drugs with human genetic evidence. In creating these figures, Nelson et al. only included indications with at least 5 genetic associations for similar traits. The set of such indications can be computed in two ways from supplementary materials. The first approach is to compute it from supplementary data (supplementary datasets 2-4). The second approach is to refer to supplementary table 5, which gives the number of similar genetically associated trait MeSH headings for each of 704 of 705 drug indication MeSH headings (Sjogren's syndrome is missing). These two approaches yield the same number of associations per MeSH term if we define the number of genetic associations to be the number of unique Link-MSH-snp_id triplets (for OMIM, snp_id, the reported SNP, is always empty so this reduces to the number of unique Link-MSH pairs). For GWAS associations, link is a PubMed id, and for OMIM associations, link is an OMIM id. We can reproduce the results of Figure 3bN and Table 1N to within reported precision using the list of traits with at least 5 genetic associations from Supplementary Table 5 (the same as the list of traits we obtained from supplementary datasets, excluding Sjogren's syndrome, which is absent from this table) and considering source "Omim" part of GWASdb.

S1.2 Sensitivity analysis

Many analysis decisions were made in the original publication that could affect conclusions, including the scope of genes and indications analyzed, the method of linking GWAS variants to genes, and the criteria for whether a gene target has genetic evidence for an indication. We can assess sensitivity to two key decisions using only data reported in the Nelson et al. supplementary materials.

The MeSH similarity parameter is used to dichotomize trait-indication pairs as similar or not similar. A compelling result from the original publication is that gene target-indication pairs are more likely to progress to the next stage when there is support for association of the target with a similar trait. This pattern is sensitive to the MeSH similarity cutoff, especially using GWASdb (S3 Fig). The chosen cutoff 0.7 appears optimal, with more confidence limits excluding zero than at nearby values of 0.5 and 0.9. The tradeoff is expected, as lower cutoff value would be expected to include both more relevant hits and more irrelevant hits. A high proportion of irrelevant hits should lower the estimated effect size, while low numbers of total hits from a high cutoff value will lead to wide confidence intervals. It is encouraging that decreasing the value to presumably include irrelevant hits removes the pattern more than increasing the value. The fact that the pattern of increasing enrichment at higher development phases for OMIM genes, but not GWAS genes, persists for unrelated traits may reflect the fact the OMIM genes are more highly enriched among approved drug targets regardless of indication (S1 Fig).

Another parameter chosen in this study was the number of genetic associations required for an indication to be included in the analysis. The original study used a value of 5. The number 5 was chosen in response to the tradeoff between the number of drug indications and the percent of indications with genetic support (Supplementary Figure

7 from the original paper). We considered cutoff values between zero (no filtering on number of genetic associations) and 50 (such that only the top 19% of indications were included).

The estimated effect of genetic support on phase-specific progression probabilities is relatively insensitive to this value, though the selected value is near an optimum for the GWAS genetic evidence progression risk ratio (S4 Fig). We still see a pattern of increasing enrichment of genetically supported targets in more advanced pipeline phases.

References

[1] Malachi Griffith et al. "DGIdb: mining the druggable genome". In: *Nature Methods* 10.12 (2013), pp. 1209–1210.

[2] Matthew R Nelson et al. "The support of human genetic evidence for approved drug indications". In: *Nature Genetics* 47.8 (2015), p. 856.

[3] Slavé Petrovski et al. "Genic intolerance to functional variation and the interpretation of personal genomes". In: *PLoS genetics* 9.8 (2013), e1003709.

[4] Alex H Wagner et al. "DGIdb 2.0: mining clinically relevant drug–gene interactions". In: *Nucleic Acids Research* 44.D1 (2015), pp. D1036–D1044.
