# Supplementary material for: Are drug targets with genetic support twice as likely to be approved? Revised estimates of the impact of genetic support for drug mechanisms on the probability of drug approval
Source: PLoS Genet. 2019 Dec 12;15(12):e1008489. doi: 10.1371/journal.pgen.1008489 (PMC6907751; doi:10.1371/journal.pgen.1008489)
Supplement: S2 Text — (PDF) [file pgen.1008489.s002.pdf]

S2 Updating Pipeline Data: Supplementary Methods And Results

S2.1 Updating Pipeline Data with New Pharmaprojects Data Freeze

Informa Pharmaprojects [1] data were obtained from an XML file of the full database from Jan 25, 2018. Following Nelson et al., we excluded drugs with nonhuman or xMHC gene targets. We considered xMHC to include HIST1H2AA and KIFC1 and all genes between them (Chromosome 6 25.7 Mb-33.4 Mb). We also excluded drugs with non protein-coding gene targets. All Entrez ids were mapped to ensembl ids and indications to MeSH headings. Unmapped indications and gene targets were excluded. The drug table was collapsed to one row per gene target-MeSH pair. The latest phase of gene target-MeSH pair (g, m) is defined to be the latest phase of any indication mapping to MeSH heading m over all drugs with target g. (g, m) is US-EU approved if there exists a drug d satisfying d is US/EU approved, d is approved for an indication mapping to MeSH heading m, and d has target g.

Processing Pharmaprojects data as outlined above does not deviate greatly from the approach used by Nelson et al. However, we made one major methodological change in how latest phase is assigned to Pharmaprojects drug-indication pairs. 66% of drug-indication pairs with human gene targets in the Pharmaprojects database are assigned an inactive status such as No Development Reported, but determining the effect of genetic evidence on clinical development progression requires the latest development phase attained by each gene target-indication pair. For this reason, inactive gene target-indication pairs were excluded from key analyses in [2]. We were concerned that excluding these drugs would bias the analysis as active drug-indication pairs have been under development for shorter time periods, on average, and therefore may not have had sufficient time to become approved. We assigned a latest historical development phase to these pairs using other fields of the Pharmaprojects database whenever possible. Our assignment procedure is detailed in the next section.

S2.1.1 Pharmaprojects latest phase assignment: Methods

We attempt to assign each drug and each drug-indication pair a latest historical phase that is one of “Preclinical”, “Phase I Clinical Trial”, “Phase II Clinical Trial”, “Phase III Clinical Trial”, “Pre-registration” or “Approved” (in order of earliest to latest development phase). “Approved” includes drugs with status “Launched”, “Registered”, or “Withdrawn” in Pharmaprojects. The latest historical development phase is the most advance phase of development a drug-indication pair has reached in any point of its history. Drugs or drug-indication pairs with status “Discontinued”, “No Development Reported”, “Not Applicable”, “Suspended”, NA, or “-” are considered to have *unknown latest phase*, and we would like to assign them a known one. We do this using other fields in the Pharmaprojects database. To distinguish between our inferred latest phase and raw Pharmaprojects data fields, we will refer to the former as the *latest phase*, and the latter as a status (disease status, global status, or country status, depending on which field is used). We will also distinguish the *global latest phase* (or status), the latest phase of a drug for any indication, and the latest phase at the indication level. Because all the main analysis of the paper are performed using indication-level phases, a phase is measured at the indication level unless otherwise specified.

Data from the event history, country status, clinical information, global status, and disease status for each drug was used in determining latest phase. The general strategy will be to find a latest phase using several different sources of information, then assign the most advanced phase over all the sources.

Assigning Phase From Event History

Many Pharmaprojects entries have an event history including changes in global and disease status. These fields have a standardized set of event types, and fairly consistent formatting of event details. Some events can be assigned a phase. Any event with event type “First Launches” or “Additional Launches” is assigned phase launched, events of type “Registration Submissions” are assigned the “Pre-registration” phase, events of type “First Registrations” or “Additional Registrations” are assigned the “Registration” phase, and event type “Withdrawn Products” is assigned the withdrawn phase. Trial phases in event histories were standardized to eliminate letters (e.g Phase Ia to Phase I) and to change combined phase trials to the later of the two phases (e.g. Phase II/III to Phase III). Clinical trial phases were found in event details using string pattern matching. Global highest phase for the event history source was found by taking the latest phase of any event for that drug. Latest indication-level phase from event history was taken to be the latest phase for any event involving that drug in which the disease name also appeared in the event description.

Assigning Phase From Country Status

Nearly all drugs have a known development status in some country (S3 Table). Unlike global status and disease status, country status almost never reverts to an inactive status such as "No Development Reported", "Discontinued" or "Suspended" (inferred from the rarity or absence of these terms). Latest global phase was determined from the country status field as the top phase for a drug over any country. Unfortunately, country status cannot be used to determine indication-level phases as it does not contain any indication information.

Assigning Phase From Clinical Trial Information

Pharmaprojects contains text descriptions of Preclinical, Phase I, Phase II, and Phase III trials in drug entries. These entries are unstructured and are (usually) empty when no trials of that phase have been performed. Therefore the presence of text in these fields is closely related to whether trials have been performed for that phase, though these fields may include trials that were planned but never completed. To reduce the possibility of error, we only used these fields to assign a phase when the text contained the name of the phase and did not contain the words "planned" or "expected." The latest phase with respect to the trial descriptions was the top trial phase with description field satisfying the required conditions. To assign preclinical phase, we only required this field have more than 5 characters (to eliminate a small number of nonsense entries). Sometimes these fields contain one or more Pharmaprojects indication terms formatted to match Pharmaprojects drug indications. The latest indication phase for a drug was assigned to be the latest stage field containing that indication term, subject to the above mentioned restrictions for quality control.

Determining Global Latest Phase From Other Fields

We have computed the latest phase of Pharmaprojects drugs using several fields. We can use this to create the global latest phase field, giving the latest known historical pipeline phase. Data from the country status and event fields are only rarely discordant with known global status (in the sense that they infrequently imply a latest phase different from the Pharmaprojects global status when the latter is a clinical phase, S4 Table). Sometimes, discordance reflects status reversions in which development ceases and then restarts at lower phase, and the event history may actually more reliably give the most advanced development phase attained by the drug. Data from the clinical trial phase description fields tend to be most discordant. Given these different degrees of error, the global latest phase for a drug was determined as follows

1. Latest phase is taken to be the most advanced development phase using the Pharmaprojects global status, country status and event status. 98.9% of drugs had a latest global phase assigned in this manner.

2. Those global latest phases that are still unknown are determined from the population of the clinical trial text fields.

3. Remaining unassigned global latest phases are retained at their original value. In all, 99.4% of drugs could be assigned a latest phase.

Using Global Latest Phase to Determine Indication Latest Phase

We used the global latest phase of a drug to assign a indication latest phase with some simple assumptions.

1. When the global latest phase is Preclinical, assume all indications are in the Preclinical phase (Pharmaprojects does not have a category lower than Preclinical).

2. When there is only one indication, the latest phase for the indication is the same as the global latest phase (assumes no indications have been omitted).

With these definitions, we assigned global latest phase whenever possible, creating a more complete dataset for evaluation (S5 Fig).

S2.1.2 Pharmaprojects Latest Phase Assignment: Results

Assigned Status By Date

99.4% of drugs and 84.6% of drug-indication pairs can be assigned a latest phase. This reduces the dramatic differences in last modified date (S6 Fig) and in the date of the first recorded drug event (S7 Fig) between drugs in clinical trials and approved drugs as compared to the approach of excluding these results used in [2]. Although reduced, there are still systematic differences in dates between drugs with unknown latest phase and other drugs.

Availability and Quality of Data by Source

When disease or global status is a known phase, it usually matches the latest phase assigned by our procedure (S5 Fig). Drug-indication pairs with inactive disease statuses (with unknown pipeline phase) tend to have lower latest phase than drugs in the dataset as a whole, with preclinical pairs more common and approved drug-indication pairs rare.

Pharmaprojects fields differ in the proportion of drugs or drug-indication pairs for which they are informative. Country status is the most readily available, while global status (for single indication and preclinical compounds) is the most informative source of data on indication-level phase (S3 Table). Both of these sources of information are highly internally consistent with Pharmaprojects global and disease status when both are a known trial phase (S4 Table). The event history is also usually internally consistent with Pharmaprojects assigned status, and commonly implies a global, but rarely an indication-level phase. Due to the sparsity of indication status information available in the event history, it is not surprising that where this information differs from the Pharmaproject disease status, it tends to be a lower status. Assigning status from the clinical trial information field shows the least agreement with global status, and there is a strong tendency for assignments using this method to have a lower trial phase, perhaps due to the filtering procedure inappropriately excluding records or due to missing information. Assignments made solely on the basis of parsing this field will be biased towards being lower than the latest development status (S4 Table).

S2.2 Supplementary Results Using Updated Pipeline Data

S2.2.1 Analysis of All Updated Pipeline Data

S8 Fig, S9 Fig and S5 Table show replication of main results with updated pipeline data.

S2.2.2 Analysis of 2013-2018 Progressions (Pipeline Progression)

S6 Table and S7 Table show the association between genetic evidence labels constructed in 2013 and pipeline progression 2013 - 2018 for target-indication pairs with active clinical trials in 2013.

S2.2.3 Analysis of Previously Unused Gene Target-Indication Pairs (New Pipeline)

S8 Table shows the association between 2013 genetic evidence labels and pipeline progression in the New Genetic test set. S9 Table and S10 Table show the same results for two different subsets of the data: those target-indication pairs absent from the Nelson et al. dataset and those target-indication pairs present in the dataset with an inactive phase.

S2.2.4 Removing similar mechanisms to 2013 Approved Drugs

Target-indication pairs supported by genetic evidence were more likely to progress from 2013-2018, and the effect was statistically significant for OMIM drugs. Nelson et al. could not have been aware of progressions that occured after the creation of their dataset, so we reason these results should be minimally affected by any overfitting to the original dataset. However, progressions from 2013-2018 may not actually be independent of pre-2013 approvals, because approved drugs may be repurposed for other indications. Target-indication pairs with similar but not identical indications might be expected to have both positively correlated approvals and positively correlated genetic evidence.

We address this concern by labelling each target-indication pair with its similarity to US/EU approved target-indication pairs in the original Nelson et al. dataset downloaded in 2013. Specifically, let $\mathcal{U}$ be the set of US/EU approved target-indication pairs in the original dataset. Define $S_A((t,g)) = \max_{p \in \mathcal{U}} S((t,g),p)$ where S is the target-indication similarity function defined in the main text methods. We will say (t,g) has a similar 2013 approved pair if $S_A(t,g) \geq 0.73$ (our recalibrated version of the Nelson et al. cutoff of 0.7). We will eliminate all such pairs from the progression dataset and recompute the effect of genetic evidence on phase progression.

As predicted, we find that 2013-2018 progression is positively associated with S_A, the trait similarity of the most similar 2013 approved target-indication pair sharing the target (not shown).

However, estimated effects of genetic evidence that were previously positive remain positive, and the positive effect of OMIM genetic evidence on Phase II to Phase III transitions increases and remains significant when target-indication pairs with similar 2013 approved target-indication pairs are removed from the dataset (S11 Table). In fact, these patterns are largely maintained even when excluding all targets with an approved indication in 2013 (S12 Table).

S2.2.5 Additional Replication Set: OMIM supplementary concepts

Our reanalysis of OMIM allows for one additional replication set. Mendelian disorders often appear in the MeSH vocabulary as supplementary concepts. 79% of OMIM traits, but no Pharmaprojects indications, were mapped to MeSH supplementary concepts. Supplementary concepts were assigned zero similarity to all drug indications because they are not part of the hierarchical structure of the MeSH vocabulary used to compute similarities (see S4 Text), and therefore did not constitute supporting genetic evidence for any drug mechanism. OMIM disorders mapping to MeSH supplementary concepts can be assigned to mapped headings supplied by MeSH. When there were multiple mapped headings, disease and psychiatric terms were preferred, and terms with higher information content were also preferred (measured by fewer descendants in the MeSH hierarchy, see S4 Text).

Estimated risk ratios of historical progression are in S13 Table. The positive effect of OMIM genetic evidence remains when mapped headings are used to identify indications similar to OMIM traits mapped to MeSH supplementary concepts.

References

[1] *Informa's Pharmaprojects.* `https://pharmaintelligence.informa.com/products-and-services/data-and-analysis/pharmaprojects`. Accessed: 2018-01-25.

[2] Matthew R Nelson et al. "The support of human genetic evidence for approved drug indications". In: *Nature Genetics* 47.8 (2015), p. 856.
