# Supplementary material for: Are drug targets with genetic support twice as likely to be approved? Revised estimates of the impact of genetic support for drug mechanisms on the probability of drug approval
Source: PLoS Genet. 2019 Dec 12;15(12):e1008489. doi: 10.1371/journal.pgen.1008489 (PMC6907751; doi:10.1371/journal.pgen.1008489)
Supplement: S3 Text — (PDF) [file pgen.1008489.s003.pdf]

S3 Updated Genetic Dataset: Supplementary Methods and Results

S3.1 Updating GWAS Dataset with GWAS Catalog and GTEx

S3.1.1 Methods

Nelson et al. Gene Table Creation and Sources Nelson et al. obtained SNP-trait associations primarily from GWASdb [7]. The list of SNPs was expanded to any SNP with $r^2 \geq 0.5$ with the reported SNP. These SNPs will be referred to as LD SNPs. LD SNPs were mapped to genes on the basis of three criteria. LD SNPs within 5 kb upstream or downstream of the gene were mapped to that gene. LD SNPs with eQTLs for the gene were mapped to that gene. Finally, LD SNPs with a DNase Hypersensitive Site (DHS) correlated with the transcription start site of the gene were mapped to that gene [10].

Recreating Gene Table with Updated Sources

Obtaining Datasets S14 Table gives URLs and download dates for all major data sources and filtering criteria used if applicable. Cutoff values were chosen to match those in Nelson et al. when available. Genetic association data was taken from the GWAS Catalog [8]. The LD SNP expansion was conducted in Plink [13] using the 1000 Genomes Project [3] Phase 3 in the EUR population. eQTL associations were GTEx significant eQTL in any tissue ($p < 10^{-6}$). DHS-gene correlations were taken from the Regulatory Element Database [14], which reported DHS activity correlations with gene expression and one-sided p-values. The RegulomeDB [1] was downloaded Oct 23, 2017. SnpEff [2] was used find genes within 5 kb of each LD SNP in hg38 and annotate potentially deleterious variant LD SNPs, including missense variants.

Linking Datasets GWAS Catalog, 1000 Genomes, and the RegulomeDB supplied SNP rs ids. GTEx variant rs ids were obtained from GTEx (GTEx_Analysis_2016-01-15_v7_WholeGenomeSeq_635Ind_PASS_AB02_GQ20_HETX_MISS15_PLINKQC.lookup_table.txt, downloaded from `https://gtexportal.org/home/datasets`). SnpEff required a vcf file as input. A vcf file for all human short variations from dbSNP build 150 was obtained from `ftp://ftp.ncbi.nih.gov/snp/organisms/human_9606/VCF/00-All.vcf.gz` and filtered to include only SNPs of interest using `vcftools` [5]. The filtered file was input to SnpEff. Gene symbols from SnpEff were mapped to ensembl ids.

Scoring Genes The above annotation procedure created datasets with pairs of LD SNPs and genes linked by eQTL, distance, or DHS correlation evidence. Additionally, each LD SNP can be annotated with its RegulomeDB score. This LD SNP annotation information was joined with a data frame of all significant SNP-trait pairs from the GWAS Catalog and a data frame linking LD SNPs and original GWAS SNPs. Gene scores (needed only for replication of Figure 2N [12], not used in main text analysis) were computed as described in [12]. When multiple LD SNPs for a GWAS hit implicated the same gene, only the highest scoring hit was retained. Finally, the dataset was filtered to retain only those genes annotated with protein coding ensembl biotype.

Mapping EFO Terms

GWAS Catalog traits are given both as a text description (disease trait) and as mapped EFO terms [9]. Linking with other sources requires a common ontology, in this case the NLM MeSH vocabulary. Using a combination of manual and automated procedures, we mapped each GWAS Catalog EFO term to MeSH. Many GWAS Catalog EFO terms describe measurements. Where possible, we mapped terms to a MeSH term describing a measurement. When not possible, we mapped to the entity being measured. For example, "bone density" maps to Bone Density but "eosiniphil count" maps to Eosiniphils.

An additional complication is that many reported genetic associations are mapped to multiple traits in EFO. We automatically assigned entries with multiple EFO terms using these rules where possible, and manually mapped additional terms.

1. Entries with a single distinct mapped MeSH heading are mapped to that heading.

2. If there are two mapped MeSH headings and one is ancestor of the other, map to the descendant.

3. Exclude EFO terms containing “blood metabolite measurement”, “clinical laboratory measurement”, “cerebrospinal fluid biomarker measurement”, “Alzheimer’s disease biomarker measurement”, “cardiovascular measurement”, “liver enzyme measurement” and assign remaining mapped MeSH term (usually a more specific measurement or measured entity).

4. Exclude EFO terms containing “onset”, “survival” and assign remaining mapped MeSH term (usually a disease)

5. If the disease trait is x in y (e.g. Body mass index in physically active individuals) or x adjusted for y (e.g. Visceral adipose tissue adjusted for BMI), map to x.

6. If only one mapped MeSH heading is a disease, map to the disease.

7. Exclude entries with disease traits containing “combine”, “interaction”, “ and ”, “pleiotropy”, “multi-trait” or “ or ” unless otherwise mapped.

8. Exclude entries with mapped EFO term “response to” (these are treatment response association studies).

17% of reported associations were excluded due to these rules or to lacking a satisfactory MeSH heading.

S3.1.2 Results

S15 Table presents data on Nelson et al. counts of distinct genes, trait MeSH headings, and SNPs compared to our results. The low overlap is striking. There are many sources of variation in the procedure.

1. Differences in the set of trait-SNP links.

2. Differences in LD expansion.

3. Differences in assigning LD SNPs to genes.

4. Differences in mapping traits to MeSH terms.

Trait SNP links As Nelson et al’s table was constructed using the May 21, 2013 version of the GWASdb (including GWAS Catalog associations), we might expect their reported GWAS hits to be a subset of ours. Discrepancies between the two data sources are reduced but still substantial when comparing 2017 GWAS Catalog associations reported before May 21, 2013 and Nelson et al. associations with source “GWAS:A” (S10 Fig, S16 Table). Though some of this lack of overlap might be explained by differences in which SNPs could be mapped to genes or which studies were included, only 63% could be found in the current verison of the GWAS Catalog. 7% of studies account for these missing SNPs and tend to have larger total reported associations.

LD expansion Both LD expansions were conducted from EUR 1000 genomes [3], but Nelson et al. used pilot phase genomes and we used Phase 3. For associated SNPs common to both analyses, 1.3% of Nelson et al. LD SNPs were not recovered in the reanalysis. Nelson et al. do not provide an exhaustive enumeration of LD SNP-SNP links so the reverse calculation is not possible.

LD SNP to Genes Among LD SNPs in common between our dataset and the Nelson et al. dataset, 81% of LD SNP-Gene links appearing in the Nelson et al dataset also appear in our dataset. Previously reported associations with eQTL evidence are less likely to be replicated in the current analysis (S17 Table). Current associations based on eQTL evidence alone are also very unlikely to appear in the previous analysis (S18 Table). Many SNPs with GTEx eQTL are not recognized by `eqtl.uchicago.edu`. Additionally, this is an older database and eQTLs may have been measured in fewer tissues and in studies with smaller sample sizes.

Disease to MeSH Discrepancies Nelson et al. provide mapping of many GWAS traits to MeSH terms in a supplementary table. From this table, around half of GWAS Catalog entries were mapped to a different disease term. It appears [12] mapped the `DISEASE.TRAIT` field rather than the `MAPPED_TRAIT` field as we did, and that they had a preference for mapping to disease terms even when the reported trait was not a disease (e.g. pulmonary function measurement mapped to lung diseases).

S3.2 Updating OMIM Dataset

Inclusion Criteria All phenotype associations with ensembl genes were obtained from the file `genemap2.txt` obtained from OMIM [11] (6275 total associations). Associations labelled as provisional were excluded (leaving 5800 associations). Drug response phenotypes and somatic mutations were also excluded (leaving 5522). Excluding drug response phenotypes is consistent with our GWAS protocol. Excluding somatic mutations was done to ensure tumor somatic mutations were not included, as whether cancer somatic variants predict successful drug targets is outside the scope of this analysis. Somatic mutations were recognized by a phenotype or mode of inheritance containing the word "somatic."

Mapping to MeSH OMIM phenotypes were mapped to MeSH using a hybrid automatic and manual assignment procedure. Automatic maps were generated by string matches to MeSH headings and supplementary concepts and by the Oxo tool [6] and reviewed for precision and accuracy. 92% of terms were automatically mapped and 8% of these were rejected after review. The remainder were manually mapped.

S3.3 Supplementary Results Using Updated Pipeline and Genetic Data

S11 Fig and S12 Fig and S19 Table recreate Nelson et al. 2015 figures using updated genetic association and Pharmaprojects datasets. Enrichment of GWAS Catalog associated gene targets among approved drugs remains, but effect sizes are somewhat lower than originally reported (S11 Fig). The pattern of increasing proportion of drug targets genetically associated with a phenotype similar to their indication through successive phases of clinical development is weaker for GWAS Catalog associations, though approved gene target-indication pairs still are the most likely to have genetic support for their targets (S12 Fig). S19 Table replicates Table 1N (discussed in main text).

eQTL p-value cutoff The eQTL p-value raw cutoff was chosen somewhat arbitrarily as 10^{-6} in any GTEx tissue [4]. Given the number of comparisons made, this cutoff is not very stringent and could possibly weaken the estimated effect of GWAS genetic evidence by adding noise to the list of gene-trait links. S20 Table estimates of the effect of GWAS genetic evidence in the Full Data set using only eQTL with p-value less than 10^{-12}. Using this more stringent cutoff reduces the number of gene-trait links from 28397 to 24702.

OMIM genetic evidence by indication There is a concern that success of OMIM at predicting successful drugs may be due to protein replacement therapies for Mendelian disease. If OMIM predictive performance is entirely due to this effect, its broader applicability is limited. We can look at how OMIM predicts success of indications that are not OMIM diseases. If it can successfully do this, we argue OMIM has broader applicability than simply indicating which gene to "add back" to reverse a Mendelian condition caused by deficiency. There are two major ways to recognize Mendelian disease indication MeSH terms. One is to use MeSH terms indexed under Congenital, Hereditary, and Neonatal Diseases and Abnormalities, though a few Mendelian diseases (e.g. von Hippel-Lindau Disease) do not appear under this heading. Another is to use MeSH terms mapped to OMIM traits. We note the latter approach should be conservative because OMIM traits that are Mendelian forms of a complex disease are mapped to the complex disease when no suitable MeSH term is available. S21 Table shows that although effect sizes are larger for OMIM and congenital diseases, OMIM genetic evidence remains associated with target-indication pair approval even when these two categories are removed from the dataset.

S3.4 GWAS Catalog Effect Size

A possible explanation for the lower effect of later GWAS evidence is a decline in GWAS Catalog effect size through time. To assess the plausibility of this hypothesis, we address the following questions:

1. Have reported GWAS Catalog effect sizes declined through time?
2. Does this reflect a genuine trend of lower effect variants being discovered later, or could it be due to systematically overestimated effect sizes in small studies conditioned on statistical significance?
3. Is there evidence this matters to drug success?

S3.4.1 Methods

Obtaining Effect Sizes GWAS Catalog reports a column `OR or BETA`. While odds ratios are a comparable scale across studies, Beta may be in different units. Therefore, we selected only rows likely corresponding to case-control studies with reported odds ratios. Such studies were recognized by

1. Containing the strings “case” and “control” in the `INITIAL SAMPLE SIZE` field.
2. All mapped EFO terms are diseases (EFO:0000408), phenotypic abnormalities (HP:0000118), symptoms (EFO:0003765), or behaviors (GO:0007610).
3. `OR or BETA` ≥ 1 (GWAS Catalog states that all reported odds ratios are greater than 1)
4. `95% CI (TEXT)` does not contain “unit”, “increase”, or “decrease”

The last two criteria were seldom violated when the first two were met, leading us to believe that the first two steps were largely effective at separating case control analyses with reported odds ratios. Note, however, that there could remain a few studies in which the reported effect size is not an odds ratio.

Multiple reported associations Some GWAS Catalog SNP-trait associations have been replicated. We can use to this assess how effect size changes with replication. From the GWAS Catalog, we obtain the earliest reported date for each SNP-trait association, its sample size and the estimated odds ration, as well as the largest study, its sample size, and the estimated odds ratio. Machine readable GWAS sample sizes were obtained from the GWAS Catalog ancestry file downloaded on June 20, 2019. A small number of GWAS Catalog entries from the download used in the rest of this paper were unavailable, and their sample sizes were manually computed from the `INITIAL SAMPLE SIZE` field. These samples sizes were used to identify the largest study reporting each association and its corresponding estimate.

S3.4.2 Results

S13 Fig shows a decline in the median odds ratio of newly reported GWAS case-control associations through time. On average first studies over report odds ratios compared to the largest sample size study (S14 Fig). The trend in declining odds ratios through time is less apparent when considering only studies with a replicate and using estimates from the largest replication, but may still be present (S15 Fig) When restricting GWAS Catalog associations to those that can be labelled as case control studies, separated by odds ratio greater than or less than 1.2, we do not detect an effect on drug approval in either the low or the high odds ratio condition (S22 Table).

References

[1] Alan P Boyle et al. “Annotation of functional variation in personal genomes using RegulomeDB”. In: *Genome Research* 22.9 (2012), pp. 1790–1797.

[2] P. Cingolani et al. “A program for annotating and predicting the effects of single nucleotide polymorphisms, SnpEff: SNPs in the genome of Drosophila melanogaster strain w1118; iso-2; iso-3”. In: *Fly* 6.2 (2012), pp. 80–92.

[3] 1000 Genomes Project Consortium et al. “A global reference for human genetic variation”. In: *Nature* 526.7571 (2015), pp. 68–74.

[4] GTEx Consortium et al. “The Genotype-Tissue Expression (GTEx) pilot analysis: Multitissue gene regulation in humans”. In: *Science* 348.6235 (2015), pp. 648–660.

[5] Petr Danecek et al. “The variant call format and VCFtools”. In: *Bioinformatics* 27.15 (2011), pp. 2156–2158.

[6] EBISPOT. *Ontology Xref Service*. `https://www.ebi.ac.uk/spot/oxo/index`. Accessed: 2018-06-08.

[7] Mulin Jun Li et al. “GWASdb: a database for human genetic variants identified by genome-wide association studies”. In: *Nucleic Acids Research* 40.D1 (2011), pp. D1047–D1054.

[8] Jacqueline MacArthur et al. “The new NHGRI-EBI Catalog of published genome-wide association studies (GWAS Catalog)”. In: *Nucleic Acids Research* 45.D1 (2017), pp. D896–D901.

[9] James Malone et al. “Modeling sample variables with an Experimental Factor Ontology”. In: *Bioinformatics* 26.8 (2010), pp. 1112–1118.

[10] Matthew T Maurano et al. “Systematic localization of common disease-associated variation in regulatory DNA”. In: *Science* 337.6099 (2012), pp. 1190–1195.

[11] Johns Hopkins University (Baltimore, MD) McKusick-Nathans Institute of Genetic Medicine. *Online Mendelian Inheritance in Man, OMIM®*. `https://omim.org/`. Accessed: 2018-06-06.

[12] Matthew R Nelson et al. “The support of human genetic evidence for approved drug indications”. In: *Nature Genetics* 47.8 (2015), p. 856.

[13] Shaun Purcell et al. “PLINK: a tool set for whole-genome association and population-based linkage analyses”. In: *The American Journal of Human Genetics* 81.3 (2007), pp. 559–575.

[14] Nathan C Sheffield et al. “Patterns of regulatory activity across diverse human cell types predict tissue identity, transcription factor binding, and long-range interactions”. In: *Genome Research* 23.5 (2013), pp. 777–788.
