# Supplementary material for: Are drug targets with genetic support twice as likely to be approved? Revised estimates of the impact of genetic support for drug mechanisms on the probability of drug approval
Source: PLoS Genet. 2019 Dec 12;15(12):e1008489. doi: 10.1371/journal.pgen.1008489 (PMC6907751; doi:10.1371/journal.pgen.1008489)
Supplement: S4 Text — (PDF) [file pgen.1008489.s004.pdf]

S4 Trait-Indication Similarity: Supplementary Methods And Results

S4.1 Methods

S4.1.1 Information Content

[3] provide an overview of semantic similarity measures in biomedical ontologies. Resnik and Lin similarities are both information content (IC) based similarity measures. IC can be computed externally from term frequencies in a corpus or through the structure of the ontology itself [5]. We computed information content using the `descendants_IC` function from the `R` package `ontologySimilarity` [1]. Let c be a term. `descendants_IC` computes information content as

$$IC(c) = -\log\left(\frac{a(c)}{N}\right)$$

Where N is the total number of terms in the ontology and $a(c)$ is the number of terms for which c is an ancestor (including itself). We chose this approach over corpus-based similarity available from `UMLS::Similarity` used by Nelson et al because the latter is not able to compute information content for terms that do not appear in 2009 MeSH, and we mapped to 2017 MeSH. Additionally, we found that corpus-based MeSH heading similarities computing using information content from the most current version of PubMed deviated more from Nelson et al. original values than did our values computed using descendant IC for similarities near the 0.7 cutoff used in the original analysis (not shown).

S4.1.2 Similarity

Resnik similarity between terms c_1 and c_2 is computed as

$$\text{sim}_{\text{res}}(c_1, c_2) = IC(MICA(c_1, c_2)) \tag{1}$$

where $MICA(c_1, c_2)$ is the common ancestor of c_1 and c_2 with the highest information content.

Lin similarity is computed as

$$\text{sim}_{\text{lin}}(c_1, c_2) = \frac{2 \times IC(MICA(c_1, c_2))}{IC(c_1) + IC(c_2)} \tag{2}$$

Nelson et al. quantified semantic similarity as the average of Resnik [4] and Lin [2] similarities each rescaled to between zero and one. Lin similarity is between zero and one, and does not need to be rescaled. We computed rescaled Resnik similarity using the following formula.

$$\text{sim}_{\text{res,norm}}(c_1, c_2) = \begin{cases} \frac{IC(MICA(c_1,c_2))}{\max_{c \in \mathcal{C}} IC(c)} & c_1 \neq c_2 \\ 1 & c_1 = c_2 \end{cases} \tag{3}$$

S16 Fig shows a portion of the MeSH vocabulary for heart diseases, with parent term Cardiovascular Diseases.

S23 Table shows similarities computed between trait pairs in S16 Fig. For example, we compute similarities between Cardiomyopathies and Arrhythmias, Cardiac as follows.

$$\begin{aligned} \text{sim}_{\text{res,norm}}(\text{Cardiomyopathies, Arrhythmias, Cardiac}) &= \frac{IC(\text{Heart Diseases})}{\max_{c \in \mathcal{C}} IC(c)} \\ &= \frac{5.07}{10.26} \\ &= 0.49 \end{aligned}$$

$$\begin{aligned} \text{sim}_{\text{lin}}(\text{Cardiomyopathies, Arrhythmias, Cardiac}) &= \frac{2 \times IC(\text{Heart Diseases})}{IC(\text{Cardiomyopathies}) + IC(\text{Arrhythmias, Cardiac})} \\ &= \frac{2 \times 5.07}{7.42 + 6.57} \\ &= 0.71 \end{aligned}$$

S4.2 Comparing Nelson et al MeSH similarities with this study

S17 Fig shows the correlation between average similarity as reported by Nelson et al. and our calculated values. Note that a similarity cutoff of 0.7 in the Nelson et al. analysis (red vertical line) is equivalent to a somewhat higher cutoff in our analysis. Using linear regression, we estimate the similarity cutoff of 0.7 used in the original analysis is equivalent to a similarity cutoff of 0.73 in our work, motivating our choice of cutoff. Clinical progression probability estimates using the previous cutoff value of 0.7 (S18 Fig) are similar to those reported in the main text.

S4.3 Effect of manually assigned similarity on approval

The MeSH vocabulary rarely provides links between diseases and related quantitative traits. To be able to use quantitative trait association studies as supporting genetic evidence, Nelson et al. manually assigned similarities to 320 trait-indication pairs. To assess the effect of manually assigned similarities on estimates of the effect of genetic evidence, we used supplementary tables to recreate the MeSH similarity matrix without manually assigned similarities, and recomputed risk ratios for the effect of GWAS genetic evidence on gene target-indication pair progression from Phase I to approval. We also did the same for our updated datasets. Finally, we looked at the effect of using only manually assigned similarities (setting all other similarities equal to zero) on our estimates. For consistency, we used the same set of well-studied MeSH indications in all estimates (rather than recomputing them for each similarity matrix). OMIM associations were not supported by genetic evidence based on manually assigned similarities, so there is no effect on estimates of OMIM genetic evidence, and this is not shown.

Results are shown in S19 Fig. Risk ratios of progression from Phase I to approval for gene target-indication pairs supported by manually assigned traits are very high at all similarity cutoff values (chosen as 0.5, 0.75, and 0.9 as these were the three manually assigned values). These appear to be driving most of the observed significant, positive effect of GWAS genetic evidence, though we still see some non-significant, positive odds ratios at higher similarity cutoff values. One hypothesis for this effect is that manually assigned similarities occur for a non-random set of indications, and these indications are more likely to be successful. However, we do not find differences in approval rate for these indications. It is also possible that automatically assigned MeSH similarities are more reliable indications of a genuine biological link between traits than traits with comparable automatically assigned similarities because of the expert knowledge going into them or that quantitative trait associations are more informative about disease mechanisms. However, the predictive power of manually assigned similarities does not replicate well using new GWAS Catalog associations to predict success (analysis New Genetic in S19 Fig), suggesting use of more objective methods is advisable.

References

[1] Daniel Greene, Sylvia Richardson, and Ernest Turro. "ontologyX: a suite of R packages for working with ontological data". In: *Bioinformatics* 33.7 (2017), pp. 1104–1106.

[2] Dekang Lin et al. "An information-theoretic definition of similarity." In: *ICML*. Vol. 98. 1998. Citeseer. 1998, pp. 296–304.

[3] Catia Pesquita et al. "Semantic similarity in biomedical ontologies". In: *PLoS computational biology* 5.7 (2009), e1000443.

[4] Philip Resnik et al. "Semantic similarity in a taxonomy: An information-based measure and its application to problems of ambiguity in natural language". In: *J. Artif. Intell. Res.(JAIR)* 11 (1999), pp. 95–130.

[5] Nuno Seco, Tony Veale, and Jer Hayes. "An intrinsic information content metric for semantic similarity in WordNet". In: *ECAI*. Vol. 16. 2004, p. 1089.
