# Supplementary material for: Are drug targets with genetic support twice as likely to be approved? Revised estimates of the impact of genetic support for drug mechanisms on the probability of drug approval
Source: PLoS Genet. 2019 Dec 12;15(12):e1008489. doi: 10.1371/journal.pgen.1008489 (PMC6907751; doi:10.1371/journal.pgen.1008489)
Supplement: S5 Text — (PDF) [file pgen.1008489.s005.pdf]

# S5 Modeling Drug Success Probability: Supplementary Methods And Results

In preliminary modeling work, we found GWAS genetic evidence *decreased* approval probability when the associated trait was dissimilar to the drug indication. In contrast, gene target-indication pairs with OMIM genetic evidence were significantly more likely to have an approved drug even if the reported trait was dissimilar to the drug indication. We suspected differences between targets linked to GWAS Catalog traits, targets linked to OMIM traits, and targets found in neither source could explain this. In this section, we fit logistic regression models to gene target-indication pair-level approval data with predictors whether or not a target is a GWAS or an OMIM gene, maximum similarity of a trait associated with the target to the indication, and target and indication-level properties (see Methods).

## S5.1 Predictors

**Highest level MeSH** All Pharmaprojects indications are associated with one or more top level terms in the MeSH disease or psychiatry and psychology hierarchy (for example, Neoplasms and Immune System Diseases). Previous work has shown differences in approval probability for drugs by indication category, for example lower approval probabilities for oncology indications [3]. S20 Fig shows approval probability against GWAS target probability for different top-level MeSH terms. Neoplasms, the most common top-level MeSH in the dataset, is associated with both lower approval and higher probability of a GWAS associated target. We use presence or absence of each top level MeSH term annotation to predict gene target-indication pair success, so gene target-indication pairs may have more than one top-level MeSH term.

**GO Annotation** Previous work has shown differences in success probability among target classes (e.g. kinases and GPCR) [6]. Pharmaprojects provides functional annotations which seemed to be predictive of success, but were not available for all targets. We translated these annotations to Gene Ontology (GO) [1][2] terms when possible, found all descendants of these terms using the `goatools` Python module [4], and then annotated each gene target with a term if it was annotated with that term or a descendant term. GO term annotations were obtained using biomaRt [7]. S21 Fig shows variation in approval and GWAS links by target class.

**Variance Tolerance** Residual Variation Intolerance scores measure tolerance of functional variation [5]. Nelson et al. showed genes with lower values of this score are more likely to be the targets of new drugs, but that accounting for this has little effect on the estimated effect of genetic evidence. We obtained RVIS scores from the supplementary tables of [5], and confirm a weak negative association of RVIS percentile with approval (S22 Fig).

**Time of First Attempt of Target** One hypothesis for lower success of GWAS gene targets is that GWAS is inspiring the choice of new targets that are either less druggable or have not had enough time to produce an approved drug. For most gene targets in the Pharmaprojects database, we can compute the earliest date at which a drug with that target was added to the database (an estimate of the length of time the target has been under development) and use this to predict whether that target occurs in least one successful drug. S23 Fig shows the relationship between the date the target was added and approval. Targets that have been under development for longer are more likely to have an approved drug. The complete lack of approvals for targets added since 2013 likely reflects the timescale of clinical development.

## S5.2 Supplementary Results

S24 Fig shows coefficient estimates (intercept, linear, and quadratic effects) for genetic evidence using different predictor subsets. The changes in coefficient estimates from including different covariates are meaningful, but the coefficient estimates are not comparable across GWAS and OMIM as trait similarity has been centered and scaled separately for each data source. S25 Fig shows the effect of including gene and indication-level predictors on the estimated relationship between indication-trait similarity and approval, which allows meaningful comparison between GWAS and OMIM. Without accounting for covariates, genes connected to a GWAS trait are systematically less likely to be the target of an approved drug relative to genes reported in OMIM when the trait and drug indication are highly dissimilar. Adding target and gene level properties to the model reduces this difference. In particular, genes reported in OMIM are more likely to be the targets of older drugs, which are more likely to be approved. Including target class annotations in the model has the largest effect on GWAS coefficient estimates. Adding all target and indication level predictors improves the WAIC model selection criterion over models without such predictors, or with only a single target and indication level predictor.

In the previous section, we showed that manually assigned similarities are highly predictive of success for 2013 data, but this does not extend to our independent replication sets. We repeat our statistical analysis without manually assigned similarities to address our concerns about their predictive validity. We find a small reduction in estimated approval odds ratios for gene target-indication pairs with genetic evidence, but the relationship is not qualitatively different (S26 Fig). We also repeat the analysis of whether or not OMIM genetic evidence is positively associated with approval for indications that do not appear in OMIM and are not congenital diseases (S27 Fig), finding little difference from excluding these indications.

# References


[1] Michael Ashburner et al. "Gene Ontology: tool for the unification of biology". In: *Nature Genetics* 25.1 (2000), p. 25.

[2] Gene Ontology Consortium. "Expansion of the Gene Ontology knowledgebase and resources". In: *Nucleic Acids Research* 45.D1 (2016), pp. D331–D338.

[3] Michael Hay et al. "Clinical development success rates for investigational drugs". In: *Nature Biotechnology* 32.1 (2014), pp. 40–51.

[4] DV Klopfenstein et al. "GOATOOLS: A Python library for Gene Ontology analyses". In: *Scientific reports* 8.1 (2018), p. 10872.

[5] Slavé Petrovski et al. "Genic intolerance to functional variation and the interpretation of personal genomes". In: *PLoS genetics* 9.8 (2013), e1003709.

[6] Hsin-Pei Shih, Xiaodan Zhang, and Alex M Aronov. "Drug discovery effectiveness from the standpoint of therapeutic mechanisms and indications". In: *Nature Reviews Drug Discovery* 17.1 (2018), p. 19.

[7] Damian Smedley et al. "The BioMart community portal: an innovative alternative to large, centralized data repositories". In: *Nucleic Acids Research* 43.W1 (2015), W589–W598.
