# Supplementary figures and images for: Are drug targets with genetic support twice as likely to be approved? Revised estimates of the impact of genetic support for drug mechanisms on the probability of drug approval
Source: PLoS Genet. 2019 Dec 12;15(12):e1008489. doi: 10.1371/journal.pgen.1008489 (PMC6907751; doi:10.1371/journal.pgen.1008489)

## All Genes

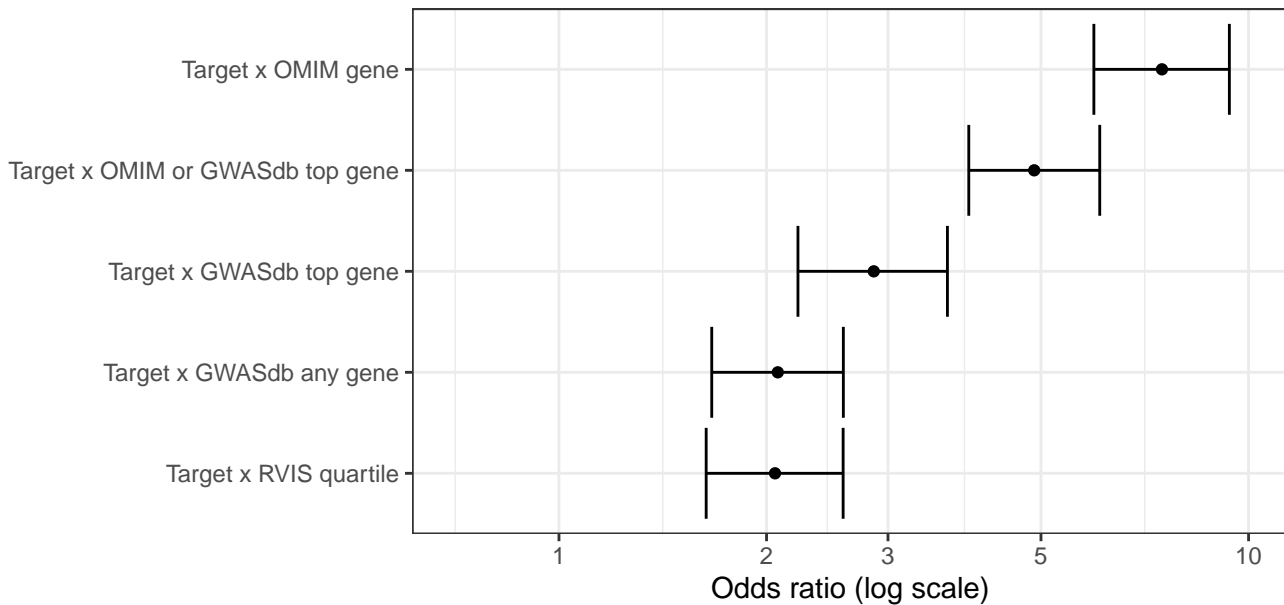

## Druggable

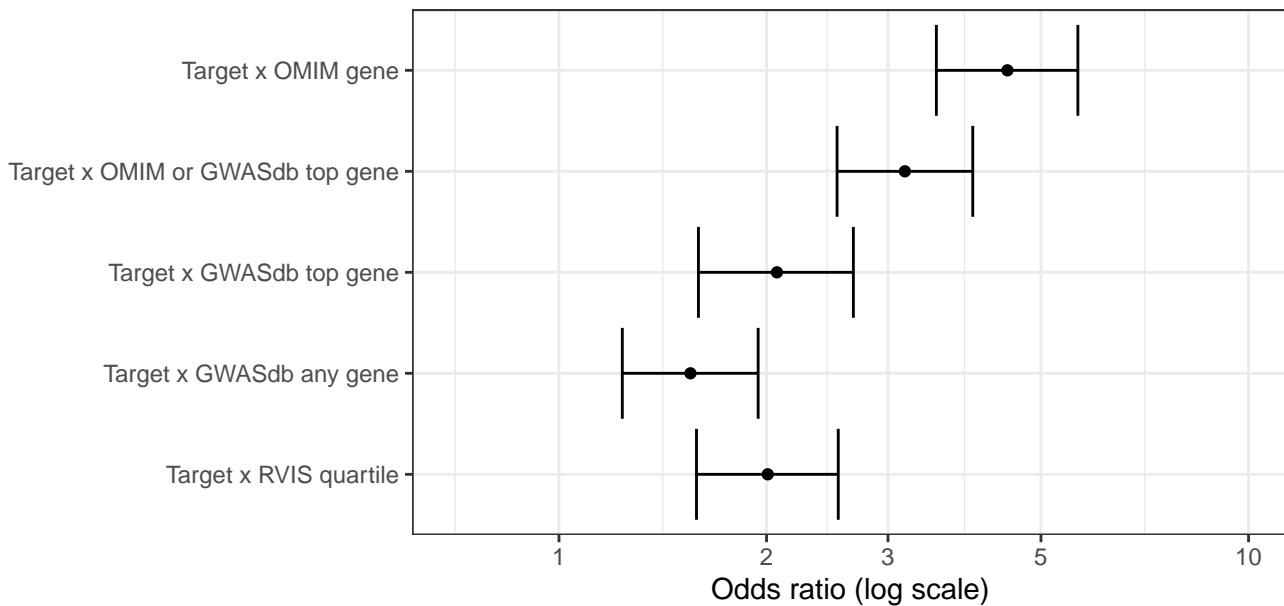

Supplement: S1 Fig — Replication of Figure 2N from Nelson et al. supplementary datasets. Figure shows the enrichment of approved drug targets among genes with human genetic associations. (PDF) [file pgen.1008489.s006.pdf]

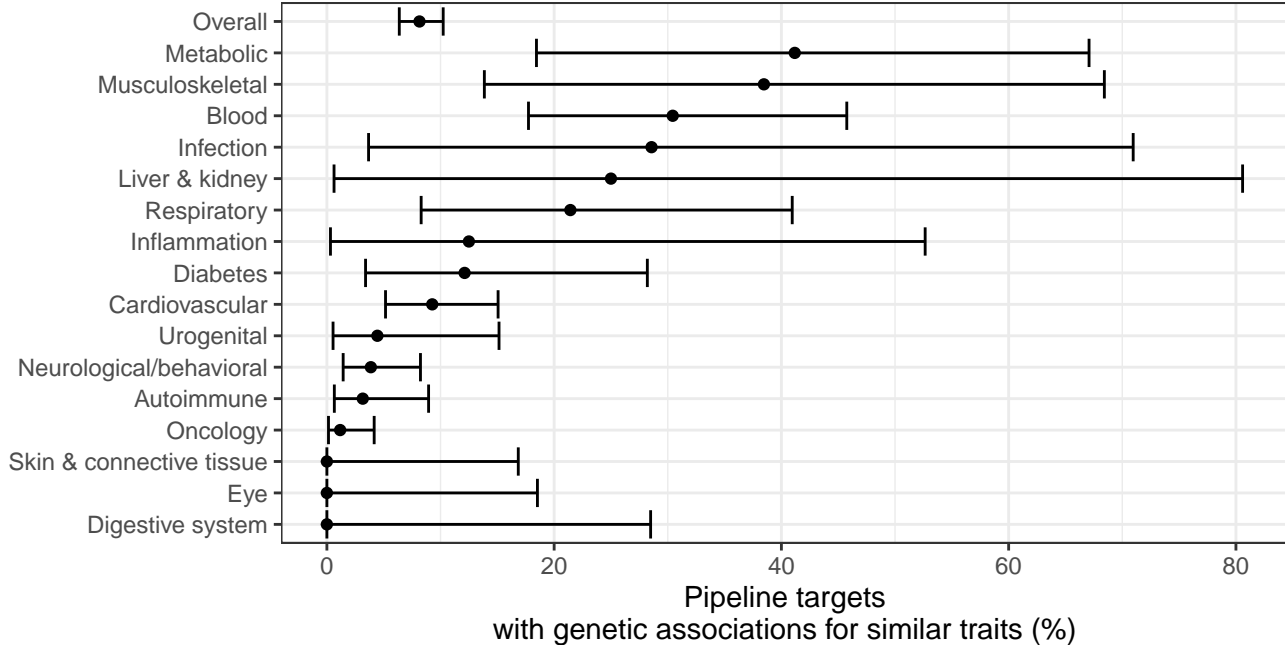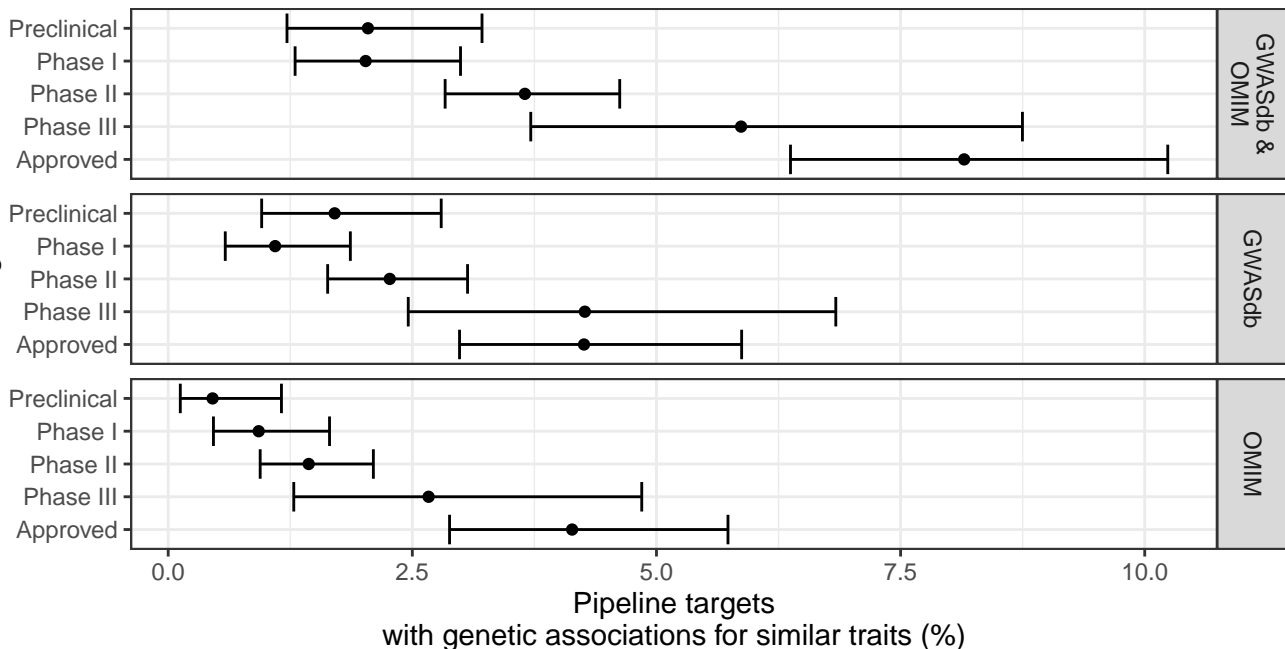

Supplement: S2 Fig — Replication of Figure 3N from Nelson et al. supplementary datasets. Figure shows the proportion of gene target-indication pairs with genetic associations for similar traits by pipeline phase and association source. (PDF) [file pgen.1008489.s007.pdf]

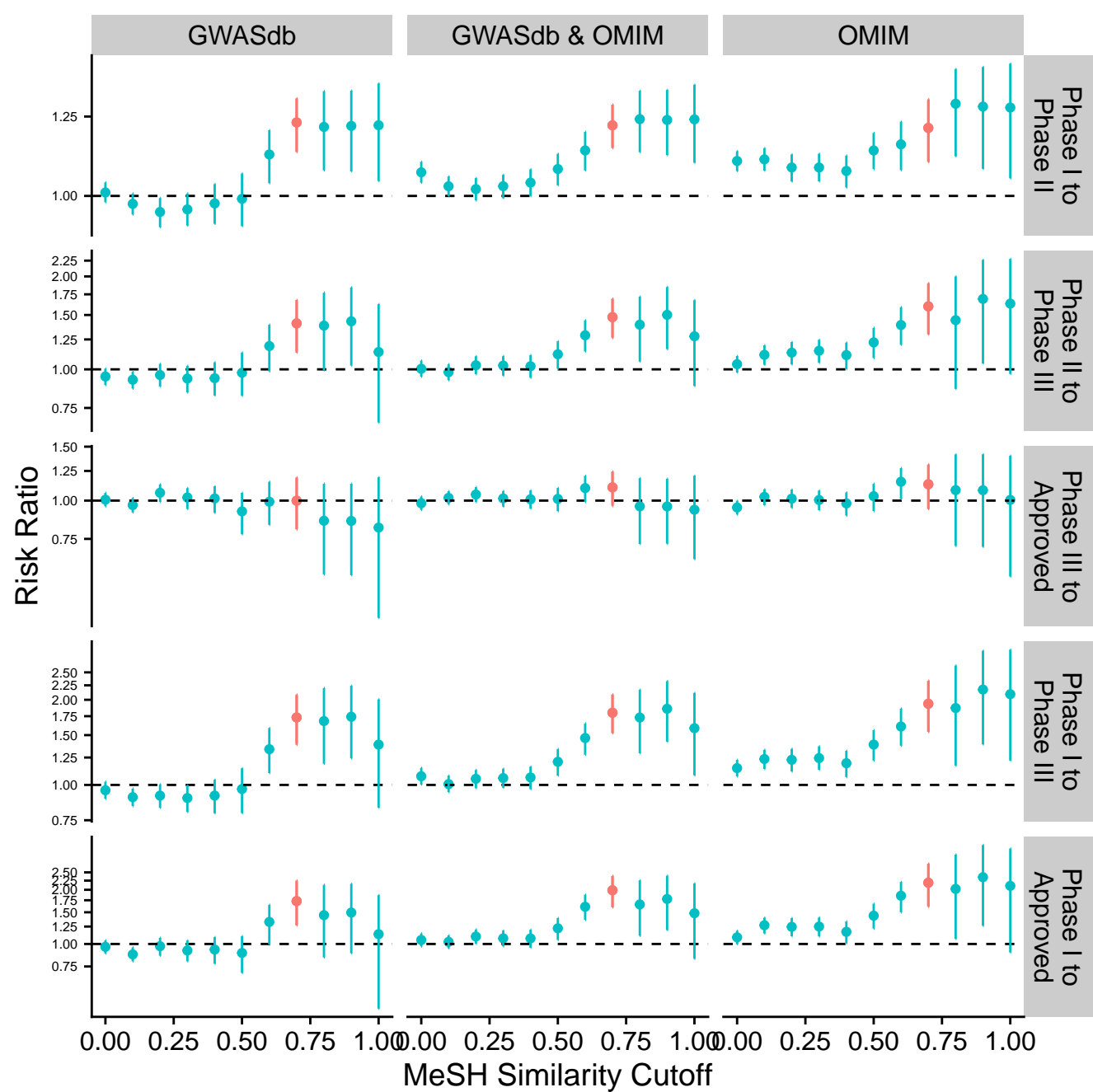

Supplement: S3 Fig — Sensitivity of risk ratios p(approved | genetic support)/p(approved | no genetic support) and 95% confidence limits to choice of MeSH similarity cutoff. Nelson et al. value 0.7 shown in red. Results are computed from Nelson et al supplementary datasets. (PDF) [file pgen.1008489.s008.pdf]

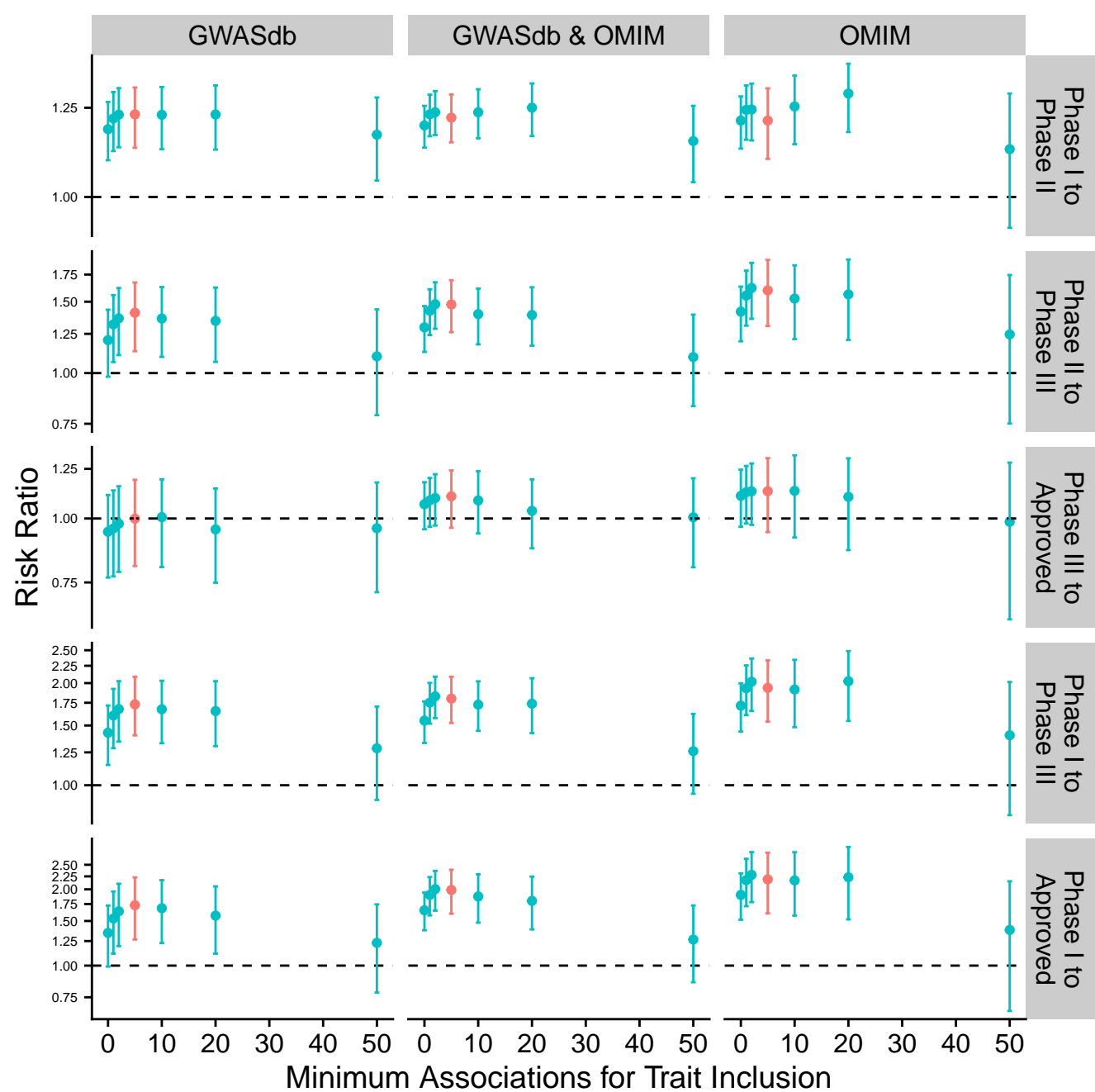

Supplement: S4 Fig — Sensitivity of risk ratios p(approved | genetic support)/p(approved | no genetic support) and 95% confidence limits to choice of minimum number of associations parameter. Nelson et al. value of 5 shown in red. Results are computed from Nelson et al supplementary datasets. (PDF) [file pgen.1008489.s009.pdf]

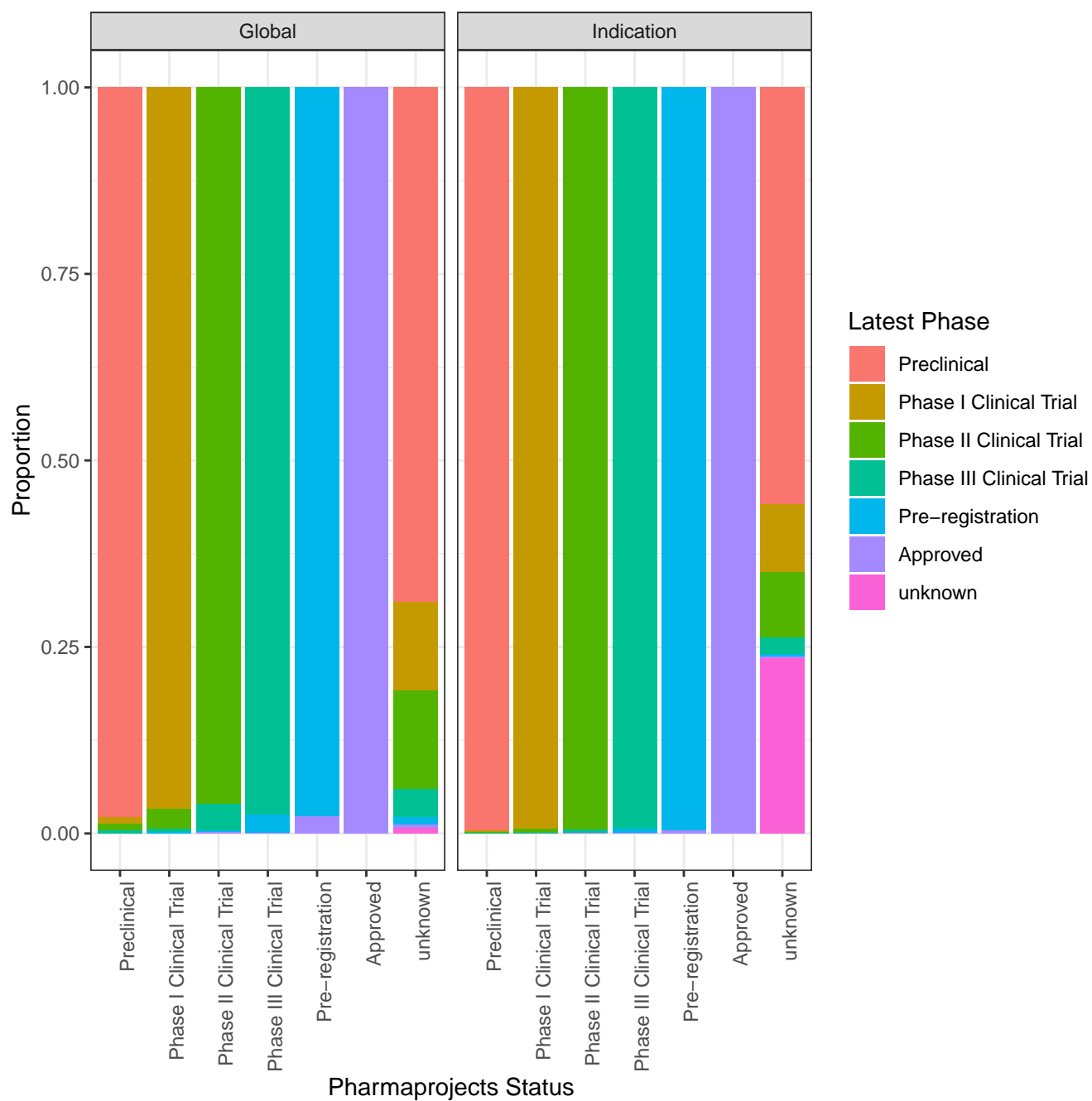

Supplement: S5 Fig — Assigned latest phase compared to Pharmaprojects status (unknown Pharmaprojects status categories such as No Development Reported and Suspended are combined) at the global and indication level. (PDF) [file pgen.1008489.s010.pdf]

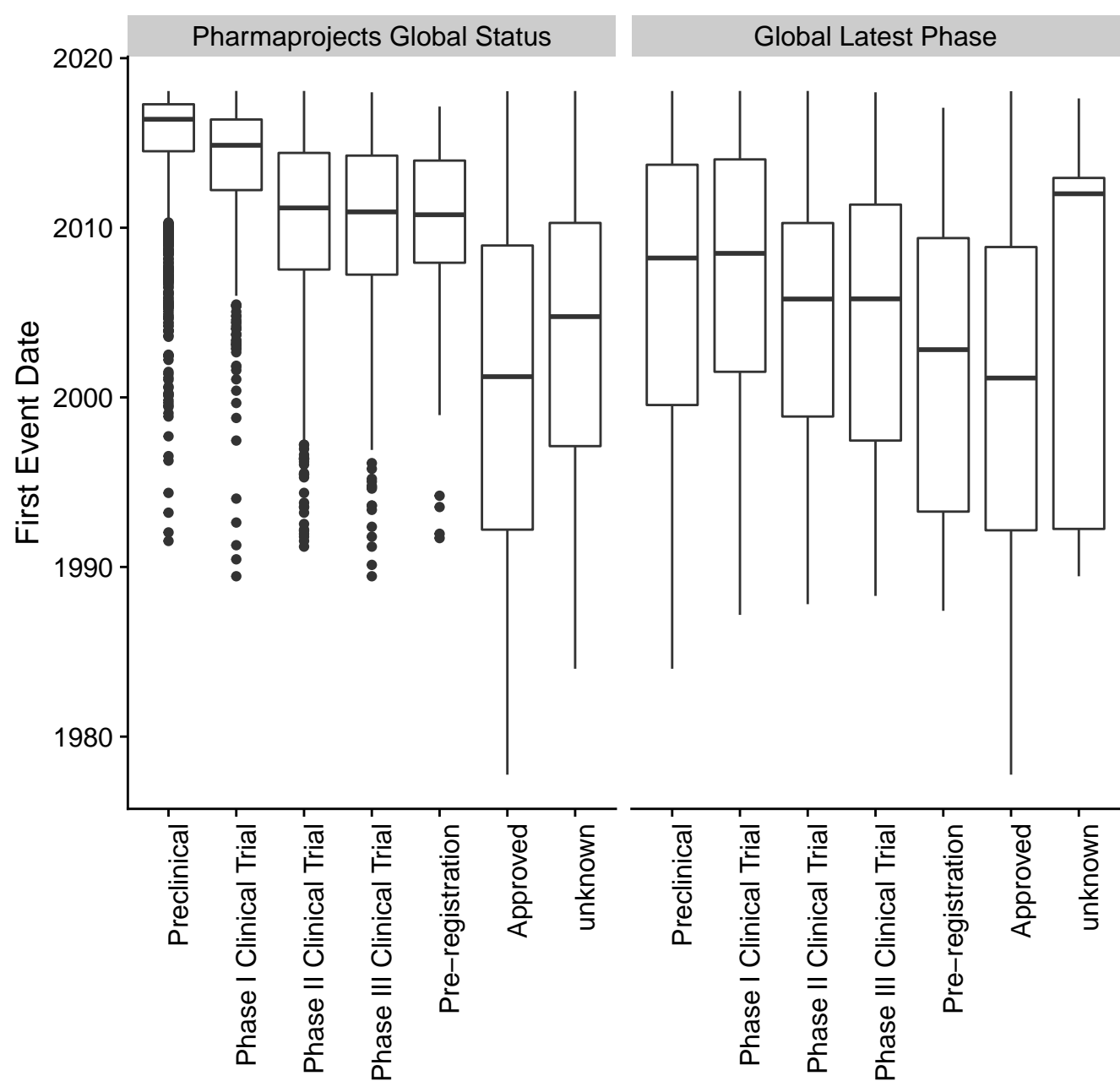

Supplement: S7 Fig — Earliest date in the Pharmaprojects event history by status. In panel Pharmaprojects Global Status, statuses come from the Pharmaprojects global status field. In panel Global Latest Phase, statuses are the latest global development phase assigned in this document. Note 6% of compounds do not have any entries in their event history and are omitted. (PDF) [file pgen.1008489.s012.pdf]

## All Genes

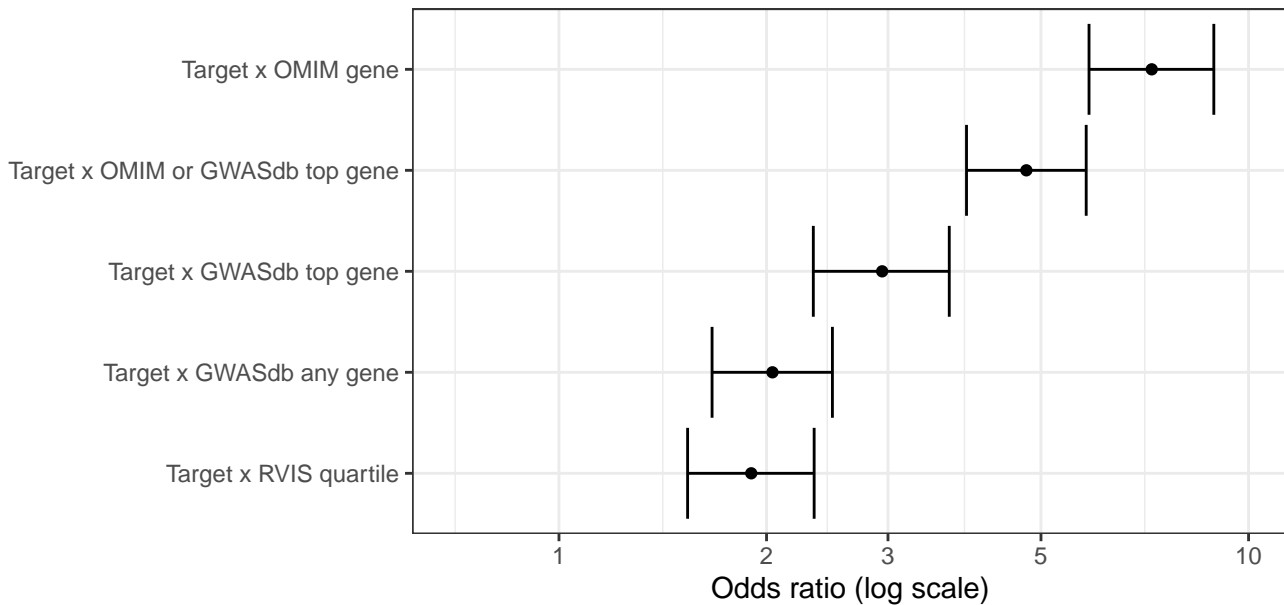

## Druggable

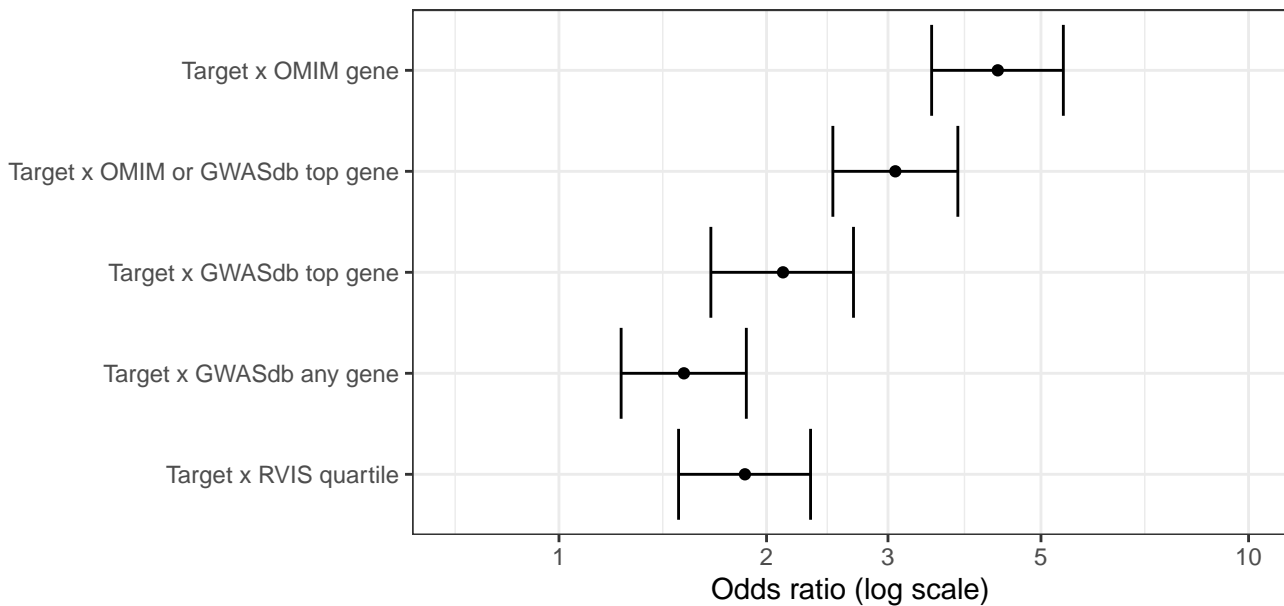

Supplement: S8 Fig — Replication of Figure 2N from Nelson et al. supplementary genetic association dataset and updated pipeline data. Figure shows the enrichment of approved drug targets among genes with human genetic associations. (PDF) [file pgen.1008489.s013.pdf]

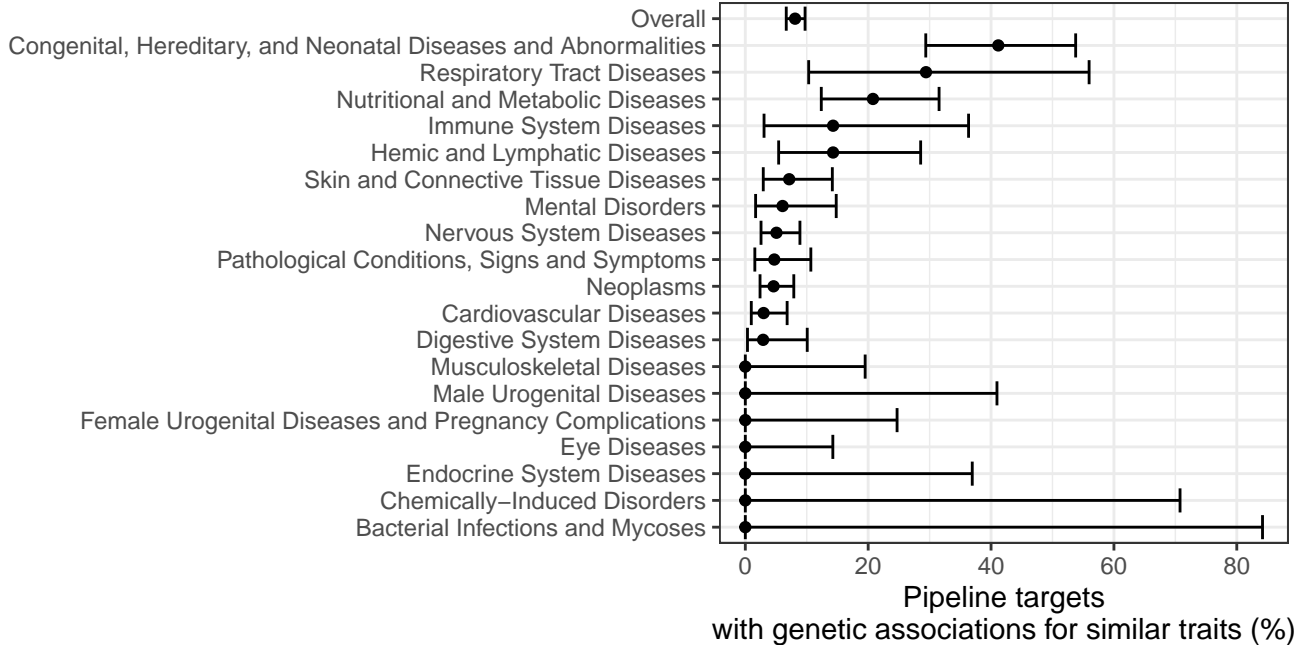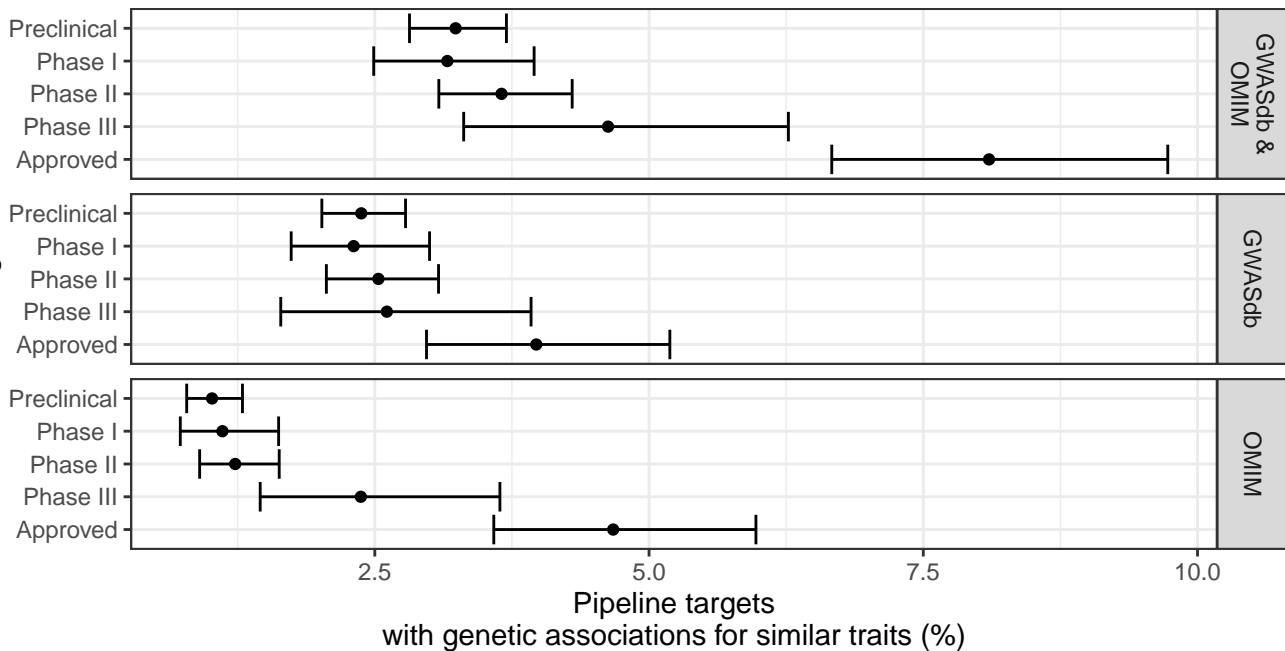

Supplement: S9 Fig — Replication of Figure 3N from Nelson et al. supplementary genetic association dataset and updated pipeline data. Figure shows the proportion of gene target-indication pairs with genetic associations for similar traits by pipeline phase and association source. (PDF) [file pgen.1008489.s014.pdf]

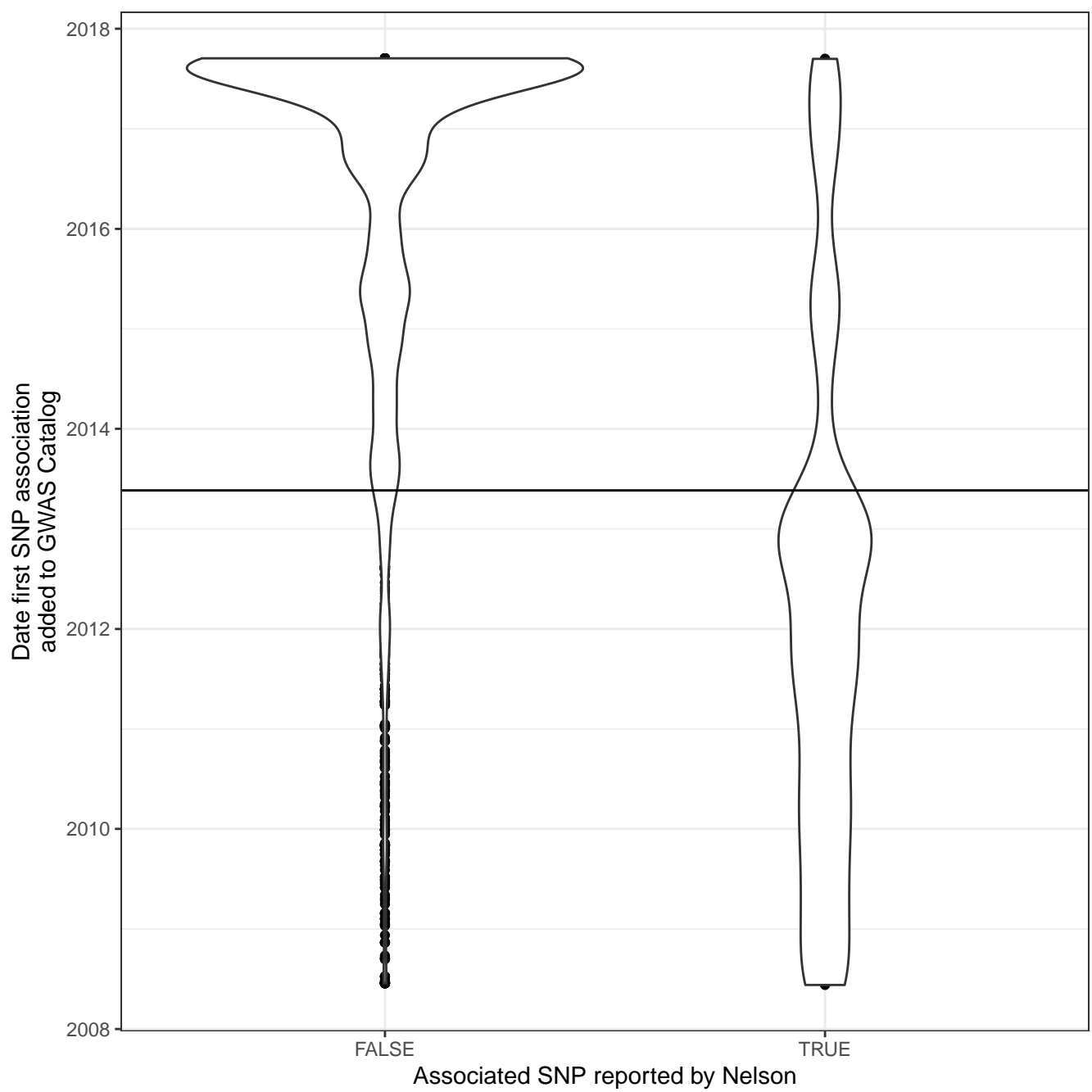

Supplement: S10 Fig — Date SNP associations appearing in the reanalysis were added to the GWAS Catalog by whether or not Nelson et al. reported the association. Line shows the date of the GWASdb version used in Nelson et al. 2013. (PDF) [file pgen.1008489.s015.pdf]

## All Genes

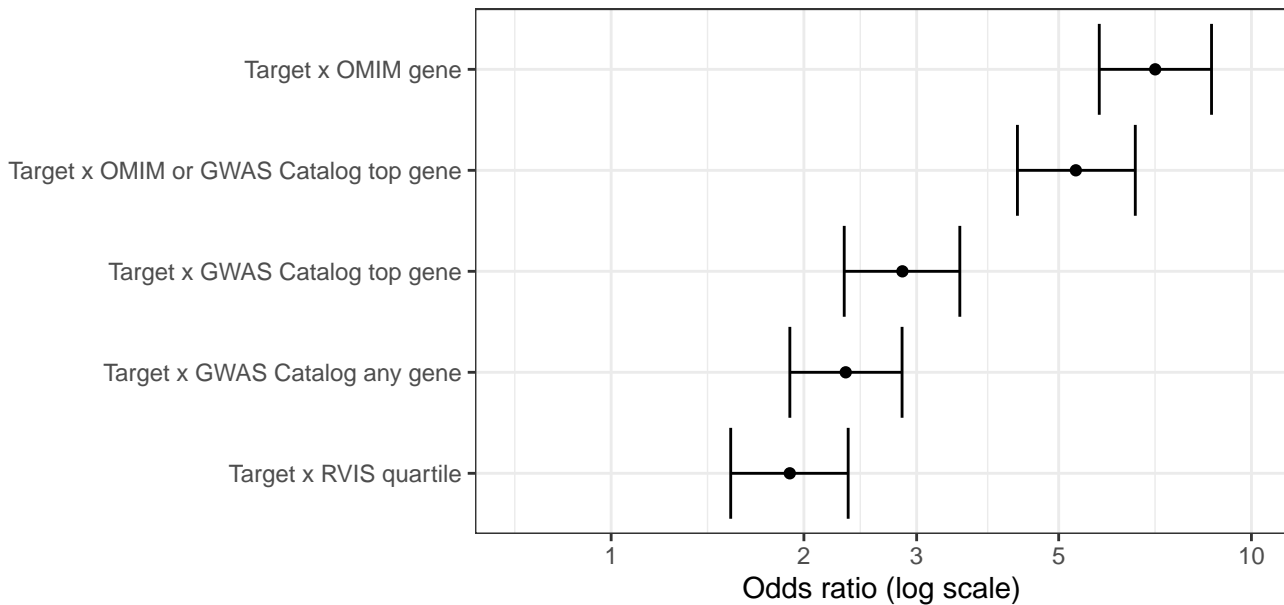

## Druggable

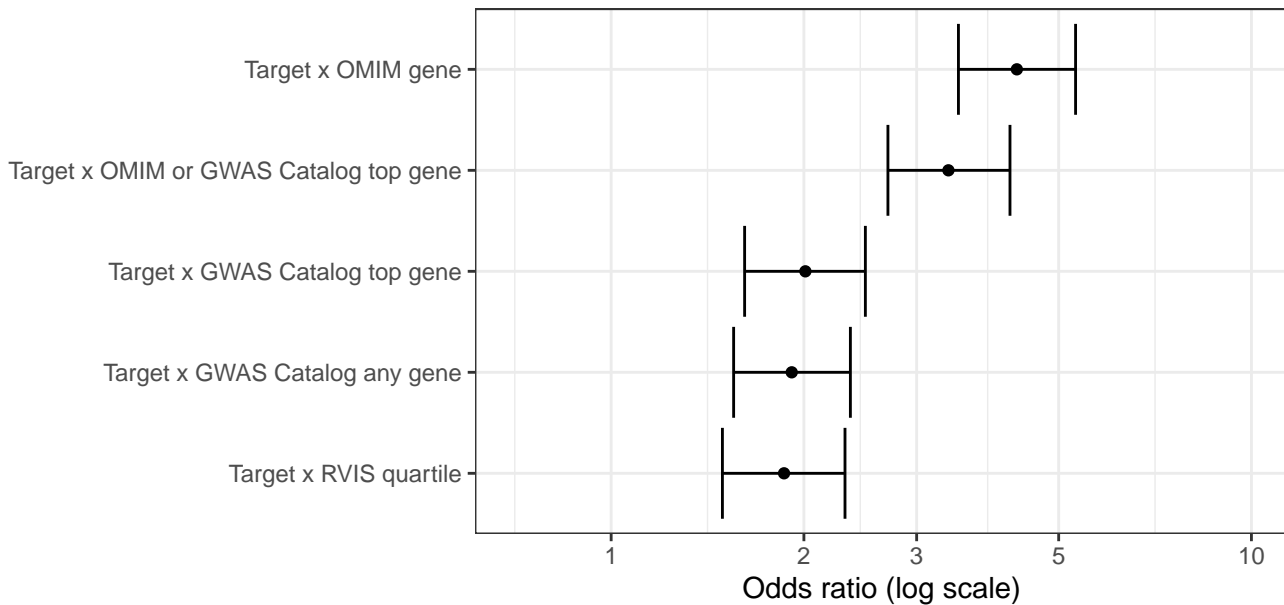

Supplement: S11 Fig — Replication of Figure 2N from updated GWAS Catalog genetic association dataset and updated pipeline data. Figure shows the enrichment of approved drug targets among genes with human genetic associations. (PDF) [file pgen.1008489.s016.pdf]

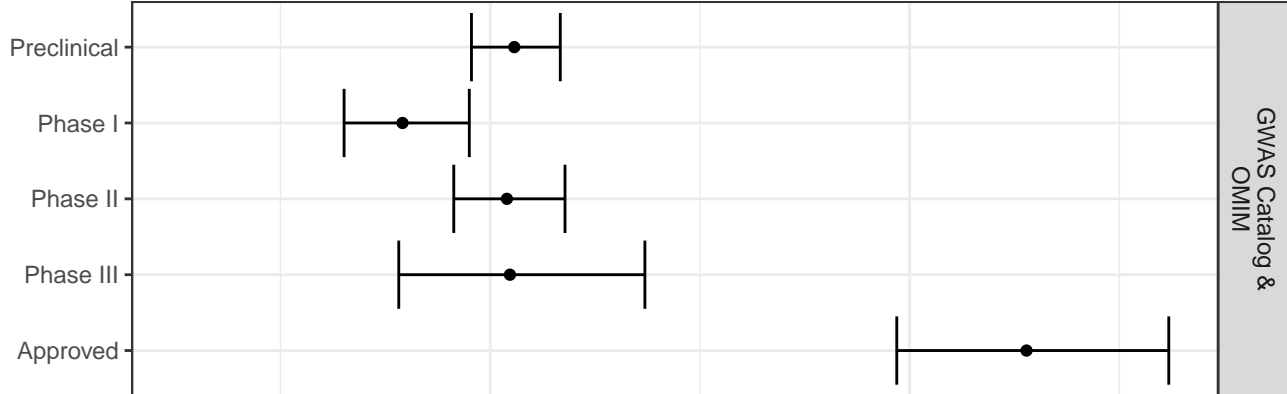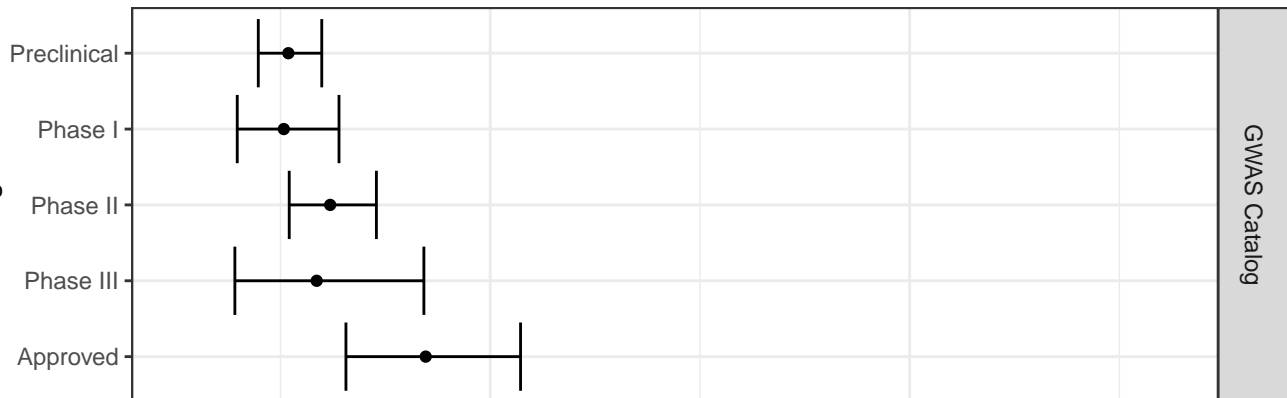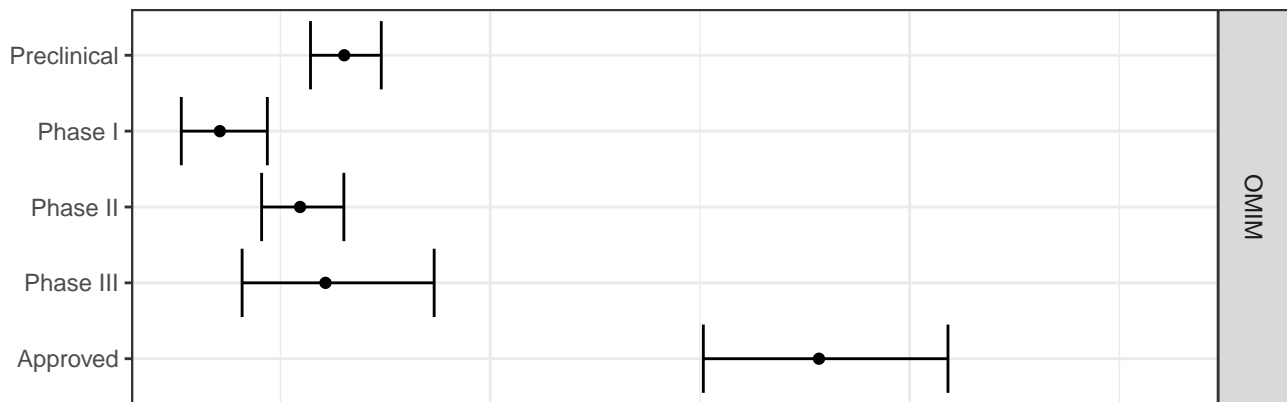

Pipeline targets  
with genetic associations for similar traits (%)

Supplement: S12 Fig — Replication of Figure 3Nb from updated GWAS Catalog genetic association dataset and updated pipeline data. Figure shows the proportion of gene target-indication pairs with genetic associations for similar traits by pipeline phase and association source. (PDF) [file pgen.1008489.s017.pdf]

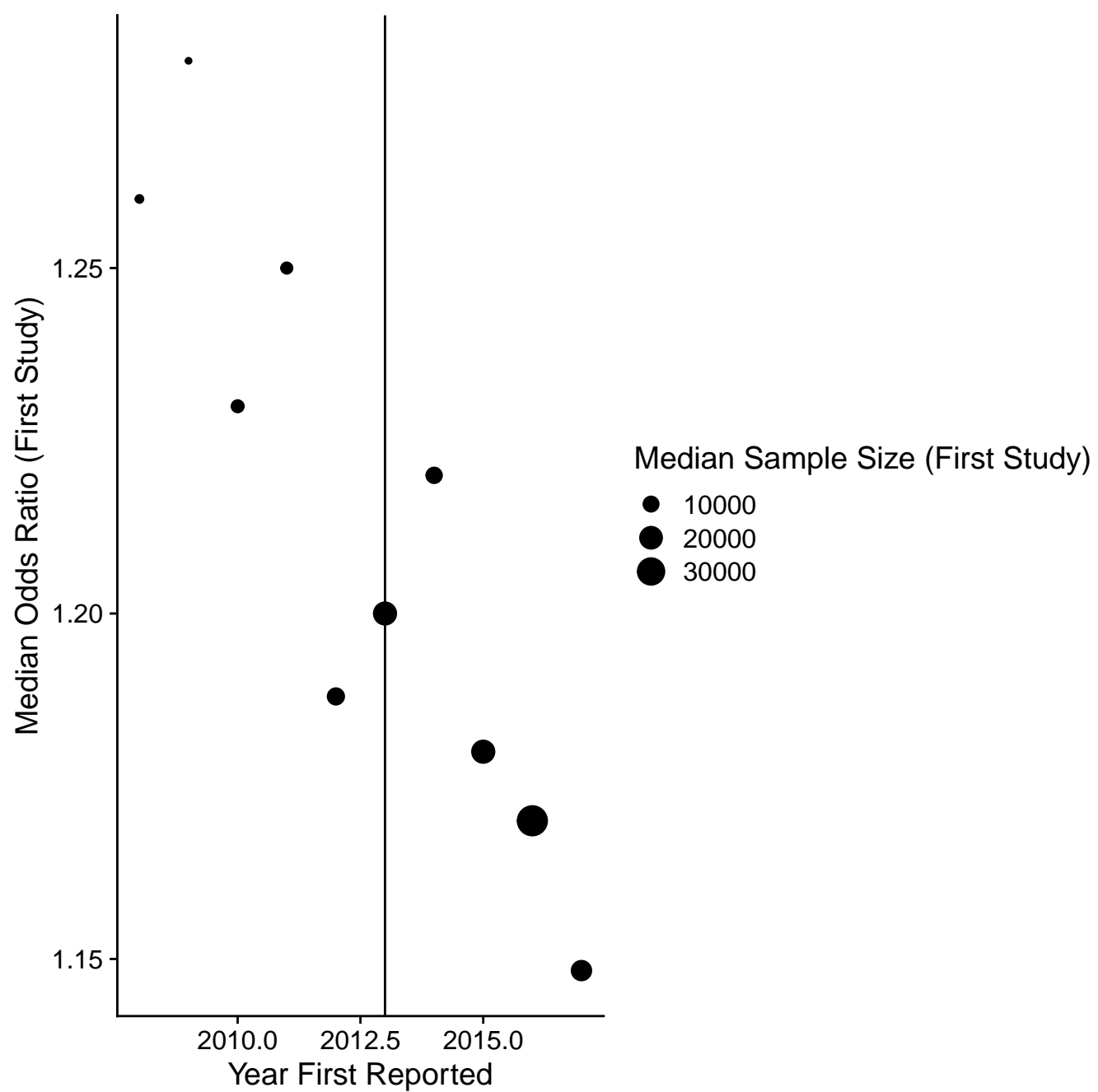

Supplement: S13 Fig — Median odds ratio for new reported case-control SNP-trait associations through time. A new reported SNP-trait association is one appearing in the GWAS Catalog for the first time, in contrast to a replicate of a previous association. (PDF) [file pgen.1008489.s018.pdf]

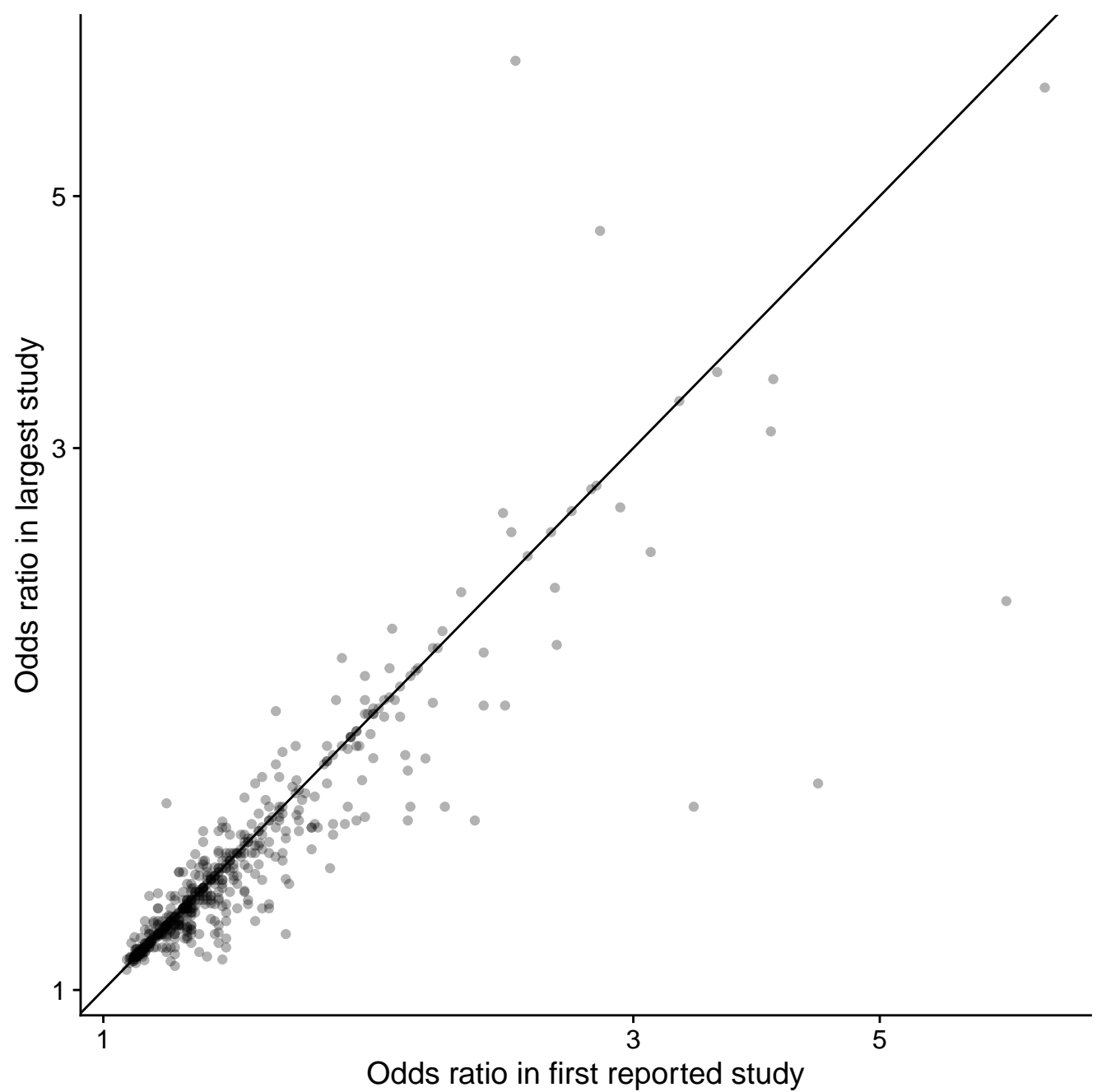

Supplement: S14 Fig — Relationship between effect size in the first study and effect size in the study with the largest sample size for GWAS Catalog case-control studies for associations that have been replicated. (PDF) [file pgen.1008489.s019.pdf]

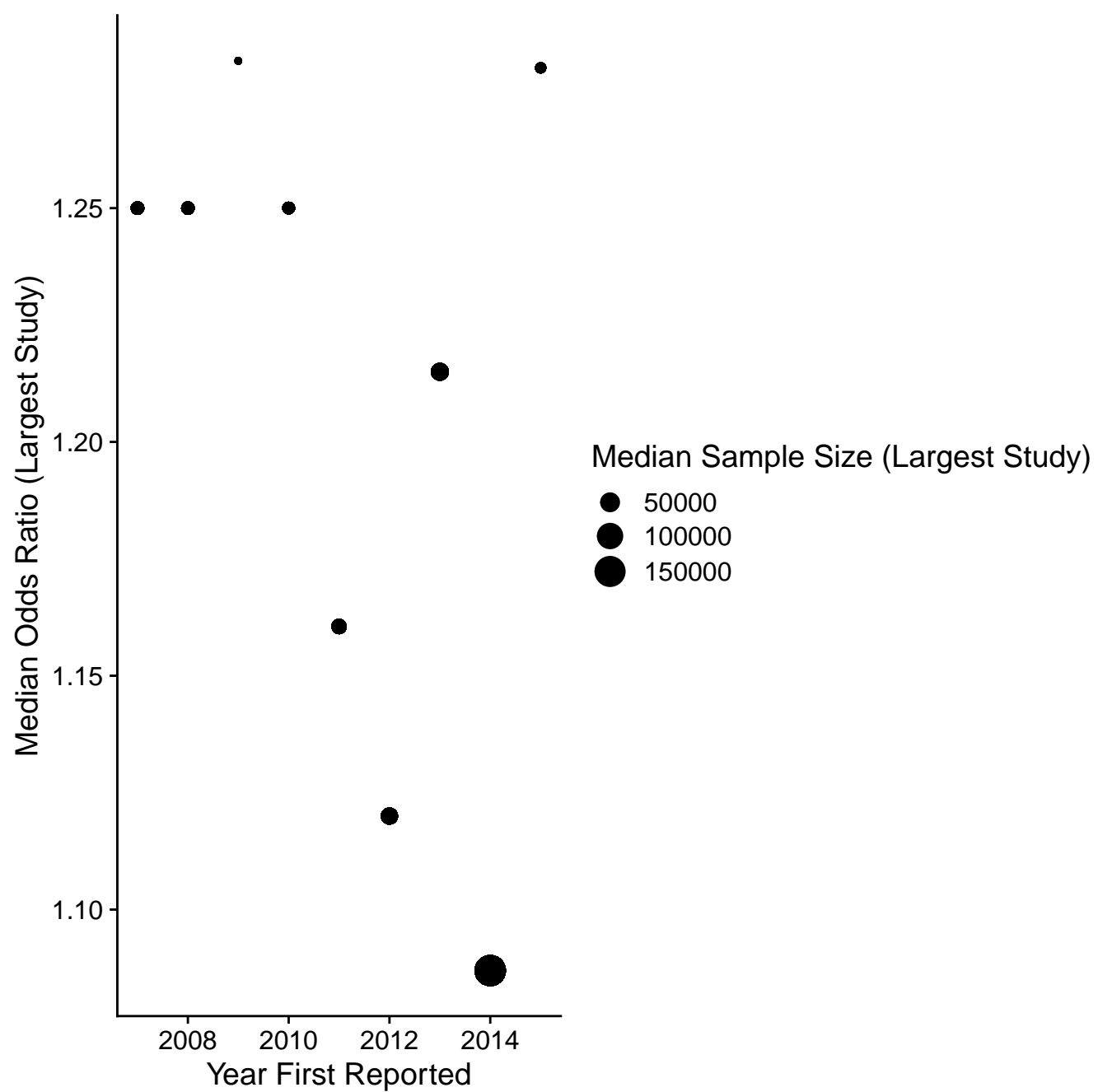

Supplement: S15 Fig — Median odds ratio for new reported case-control SNP-trait associations through time. A new reported SNP-trait association is one appearing in the GWAS Catalog for the first time, in contrast to a replicate of a previous association. Only studies with a later replicate are considered and the effect size reported is from the largest replicate. Only years with at least 20 replicated studies are shown. (PDF) [file pgen.1008489.s020.pdf]

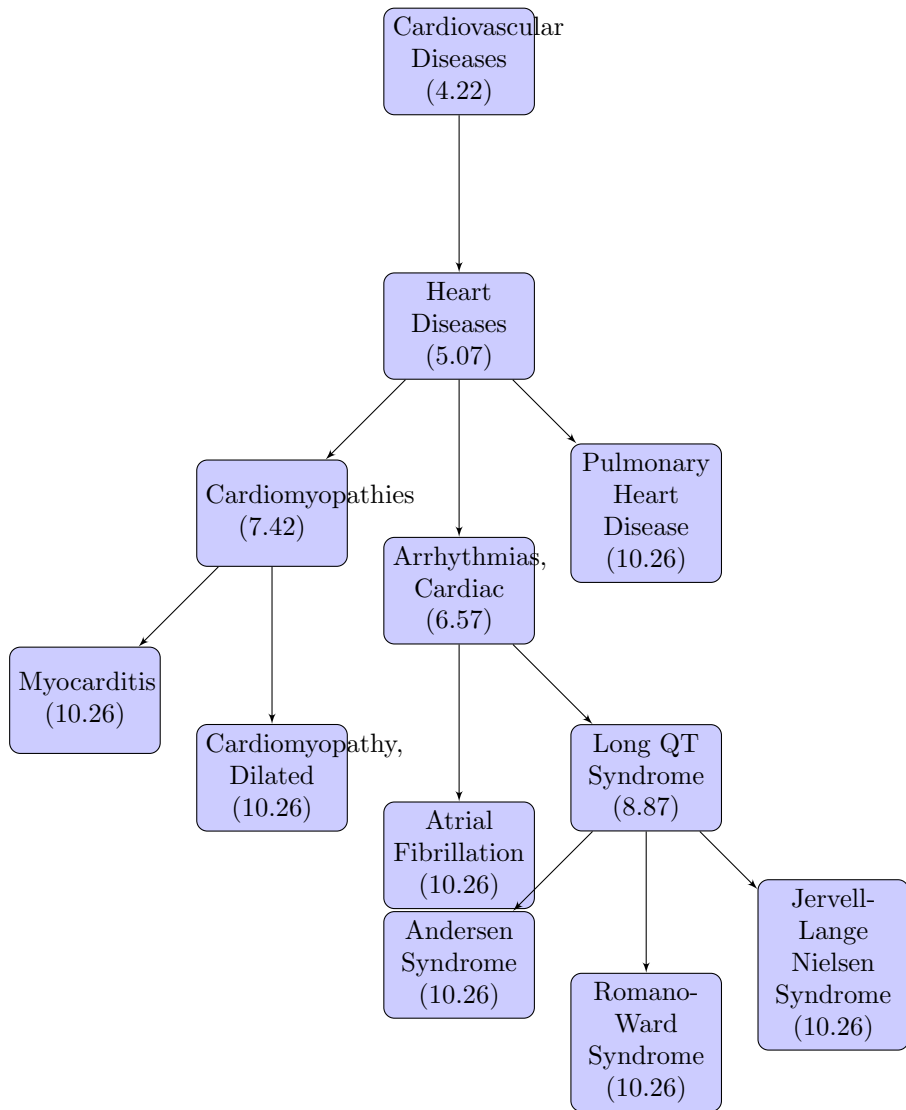

Supplement: S16 Fig — A portion of the MeSH vocabulary used to illustrate semantic similarity. Information contents from the number of descendants (computed from the entire ontology, not just the portion shown here) are given in parentheses. (PDF) [file pgen.1008489.s021.pdf]

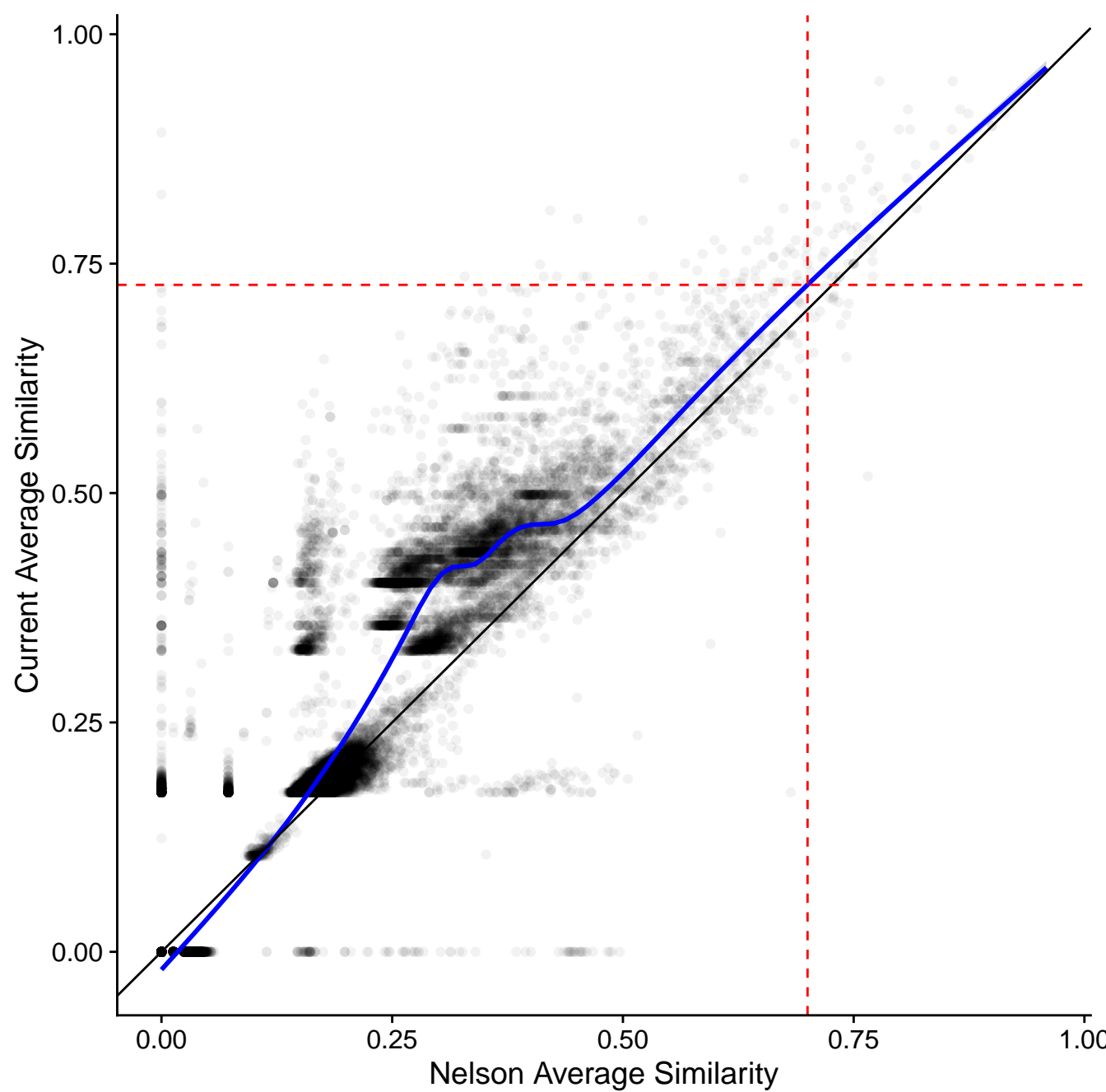

Supplement: S17 Fig — Nelson et al. semantic similarity versus semantic similarity (average of Lin and Resnik similarities) computed in this analysis. Black points show a random sample of 50,000 trait pairs for which both similarities were available. Blue line shows smoothed relationship estimated using all possible similarity pairs. Dashed red lines show old and new cutoff values. (PDF) [file pgen.1008489.s022.pdf]

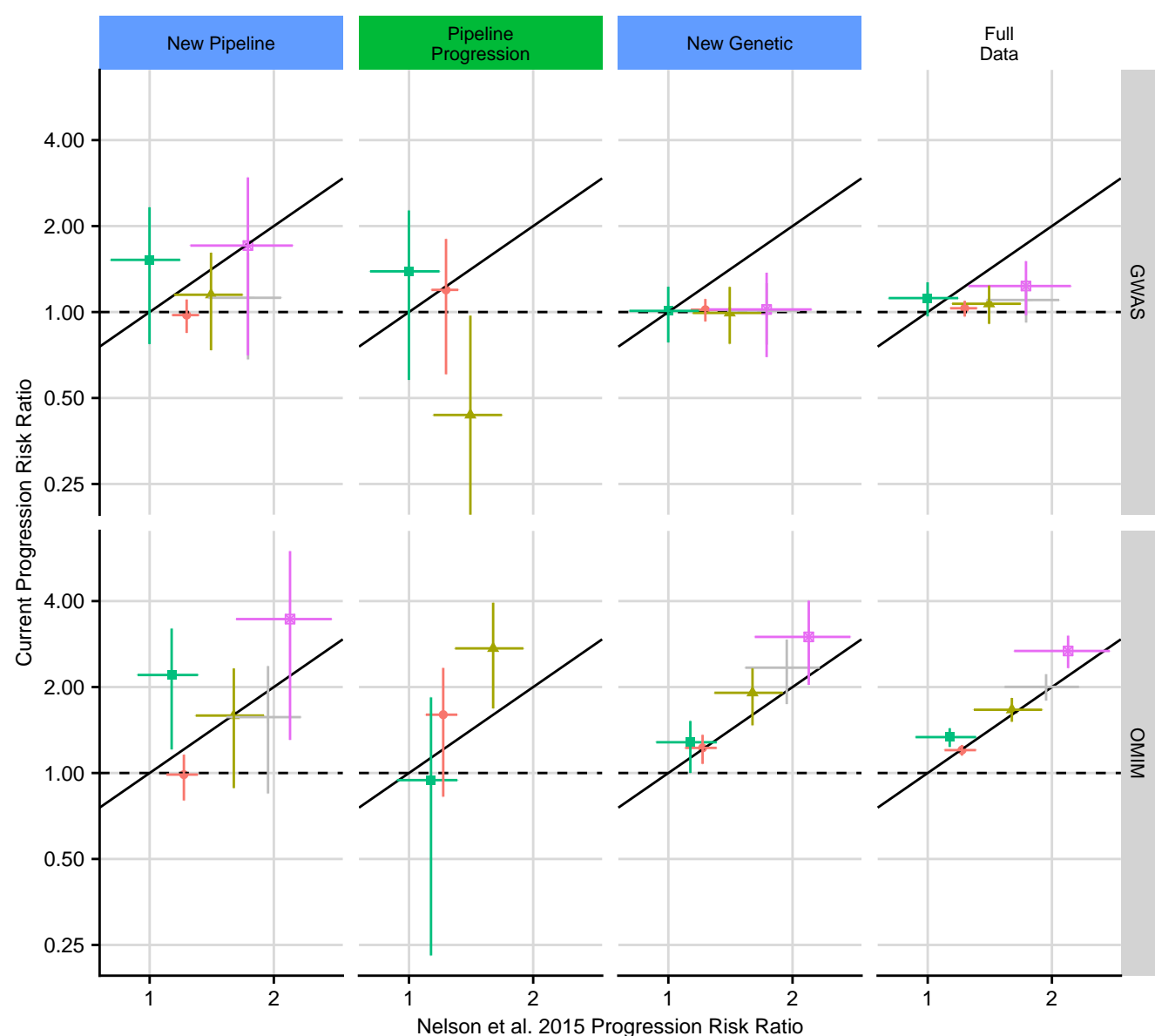

Supplement: S18 Fig — Estimated effect of genetic evidence on pipeline progression. Main text Figure 1b computed with similarity cutoff 0.7. This cutoff was originally used in Table 1N, but our main text figure uses similarity cutoff 0.73 because of systematic differences in computed similarities. (PDF) [file pgen.1008489.s023.pdf]

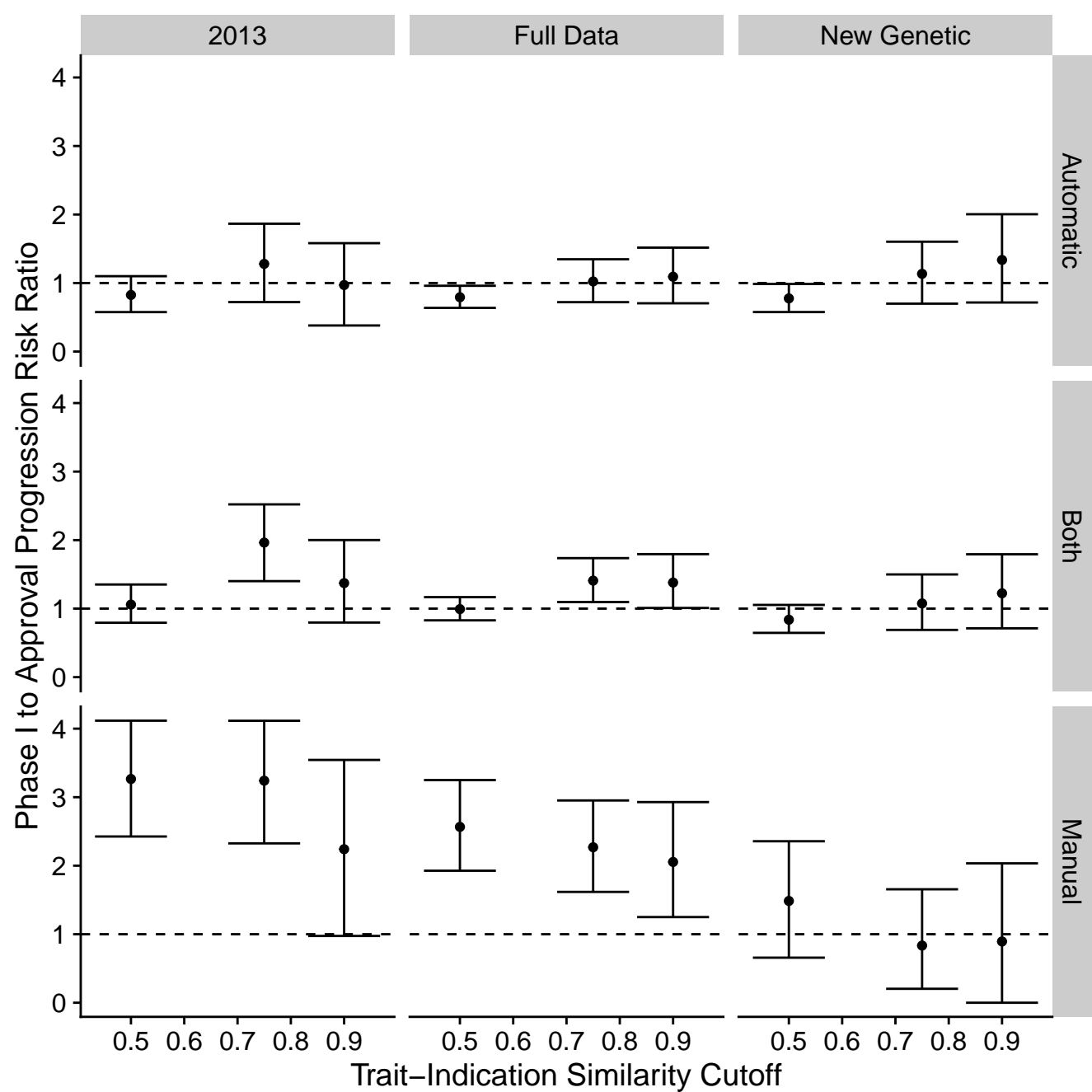

Supplement: S19 Fig — Risk ratio of Phase I to approval progression for gene target-indication pairs with and without genetic evidence for different values of the MeSH similarity cutoff split by whether MeSH similarity is automatically assigned, manually assigned, or using both automatic and manually assigned similarities (default). Full Data and New Genetic are as described in main text. 2013: computed from supplementary tables. (PDF) [file pgen.1008489.s024.pdf]

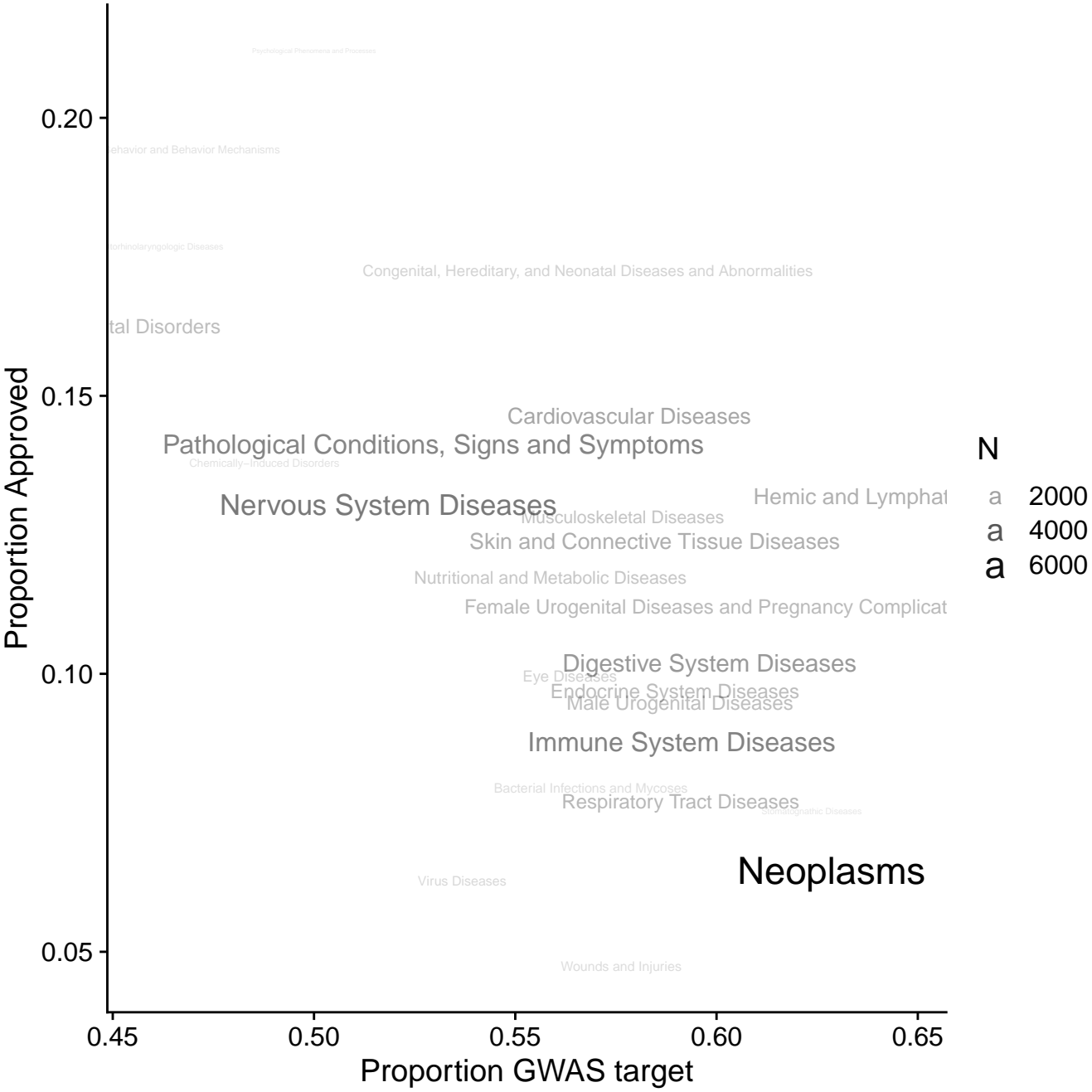

Supplement: S20 Fig — Proportion of gene target-indication pairs approved against proportion of gene target-indication pairs with a GWAS Catalog associated target by indication class. Larger text designates indication classes with more gene target-indication pairs. Only classes with 50 or more pairs are shown. (PDF) [file pgen.1008489.s025.pdf]

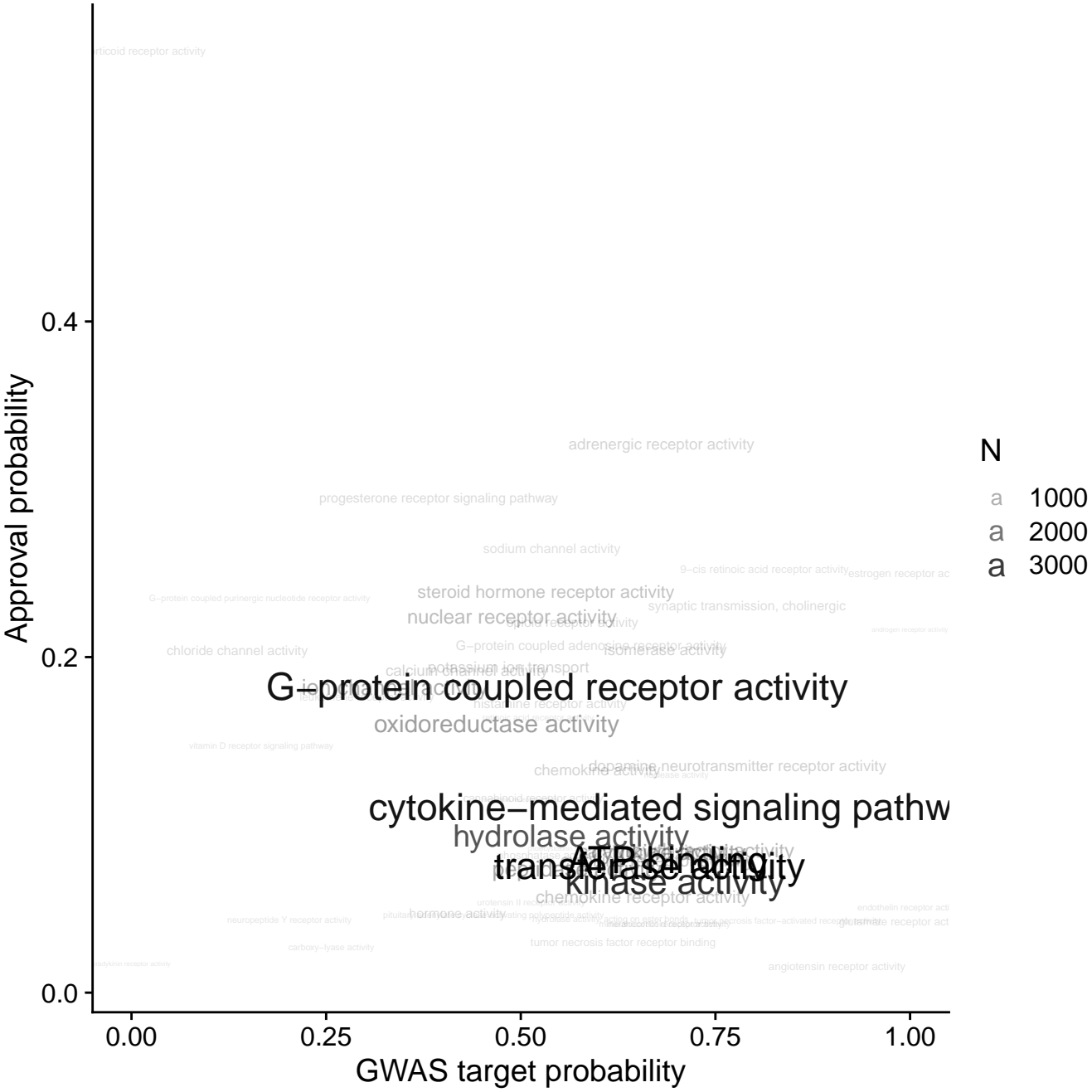

Supplement: S21 Fig — Proportion of gene target-indication pairs approved against proportion of gene target-indication pairs with a GWAS Catalog associated target by target class (GO terms). Larger text designates target classes with more gene target-indication pairs. Only classes with 50 or more pairs are shown. (PDF) [file pgen.1008489.s026.pdf]

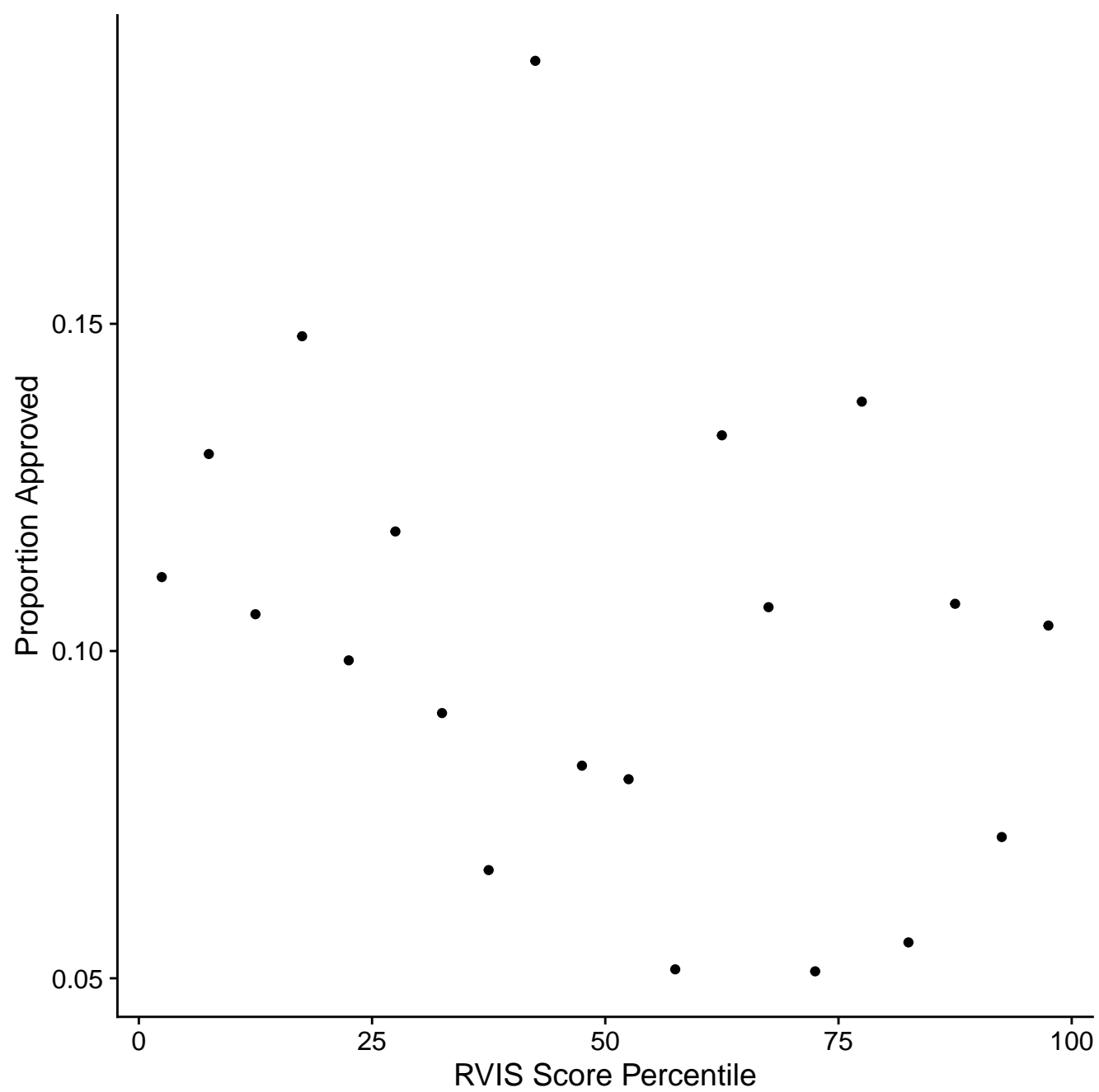

Supplement: S22 Fig — Proportion of approved gene target-indication pairs binned by target RVIS score percentile. (PDF) [file pgen.1008489.s027.pdf]

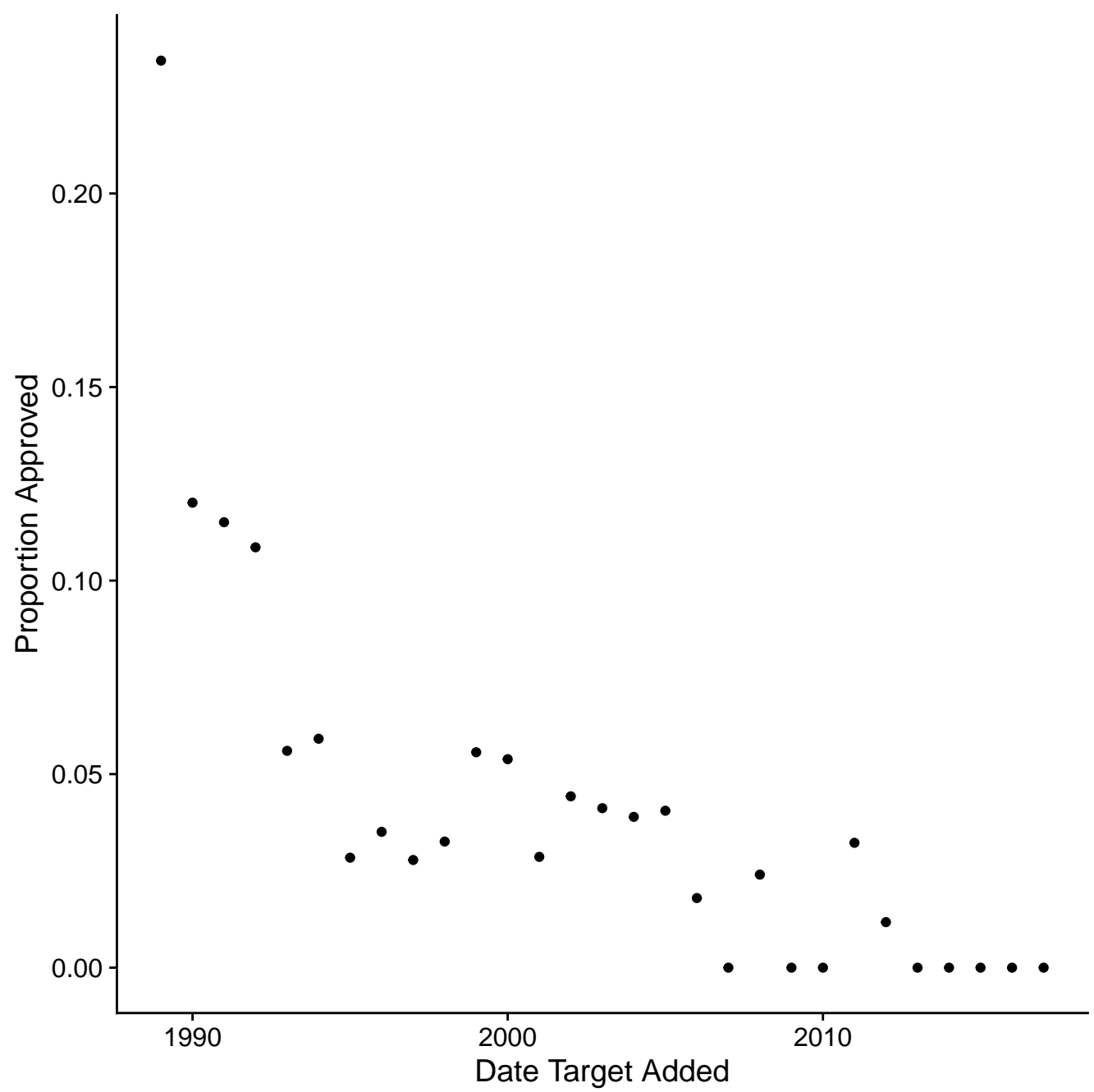

Supplement: S23 Fig — Proportion of approved gene target-indication pairs binned by date first drug with target added to Pharmaprojects. (PDF) [file pgen.1008489.s028.pdf]

Coefficient Posterior Mean

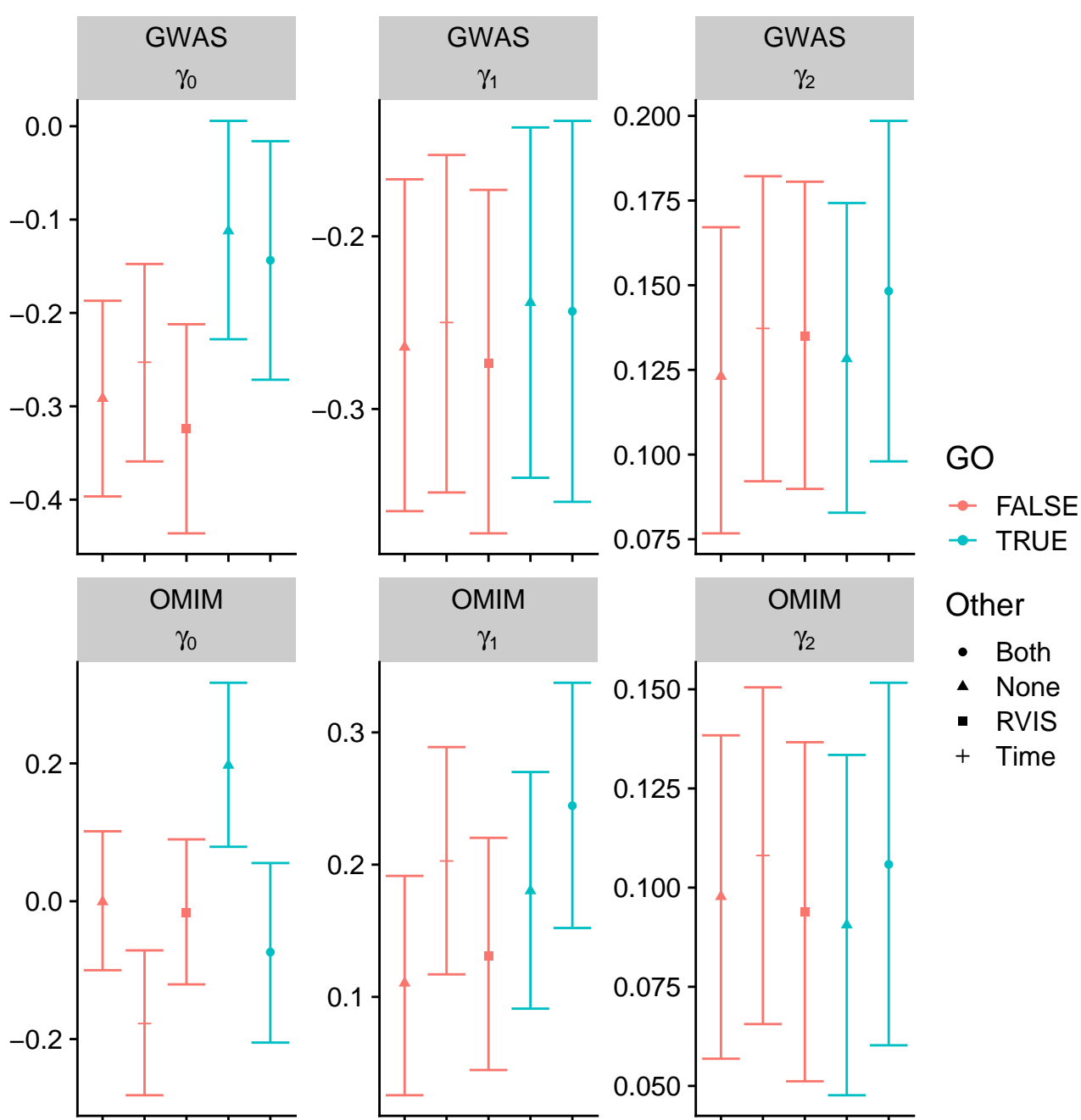

Supplement: S24 Fig — Coefficient estimates for the effect of genetically associated trait similarity on gene target-indication pair approval using different predictor subsets. See Methods for details of coefficient definitions. Note coefficients apply to centered and scaled trait-indication similarity so that the intercept for GWAS genetic evidence is the effect of genetic evidence at the mean value of GWAS trait similarity. GO = Gene Ontology terms, RVIS = RVIS score, Time = Time since target entered development. (PDF) [file pgen.1008489.s029.pdf]

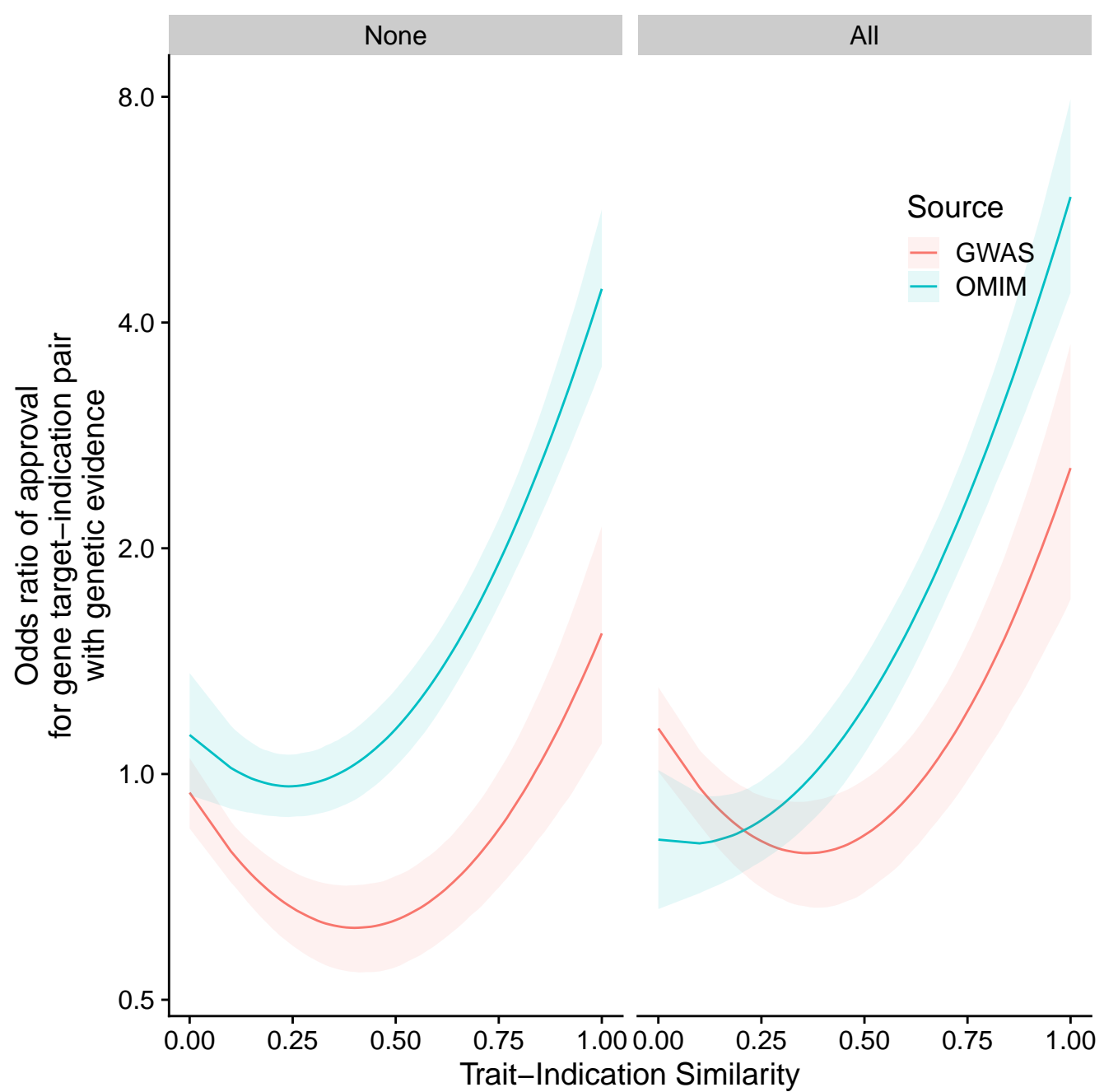

Supplement: S25 Fig — Effect of including predictors on the estimated relationship between indication-trait similarity and approval. Estimated odds ratio of gene target-indication pair attaining approval, as a function of similarity between drug indication and the most similar trait associated with the target. Posterior median and pointwise 95% credible interval from Bayesian logistic regression. (PDF) [file pgen.1008489.s030.pdf]

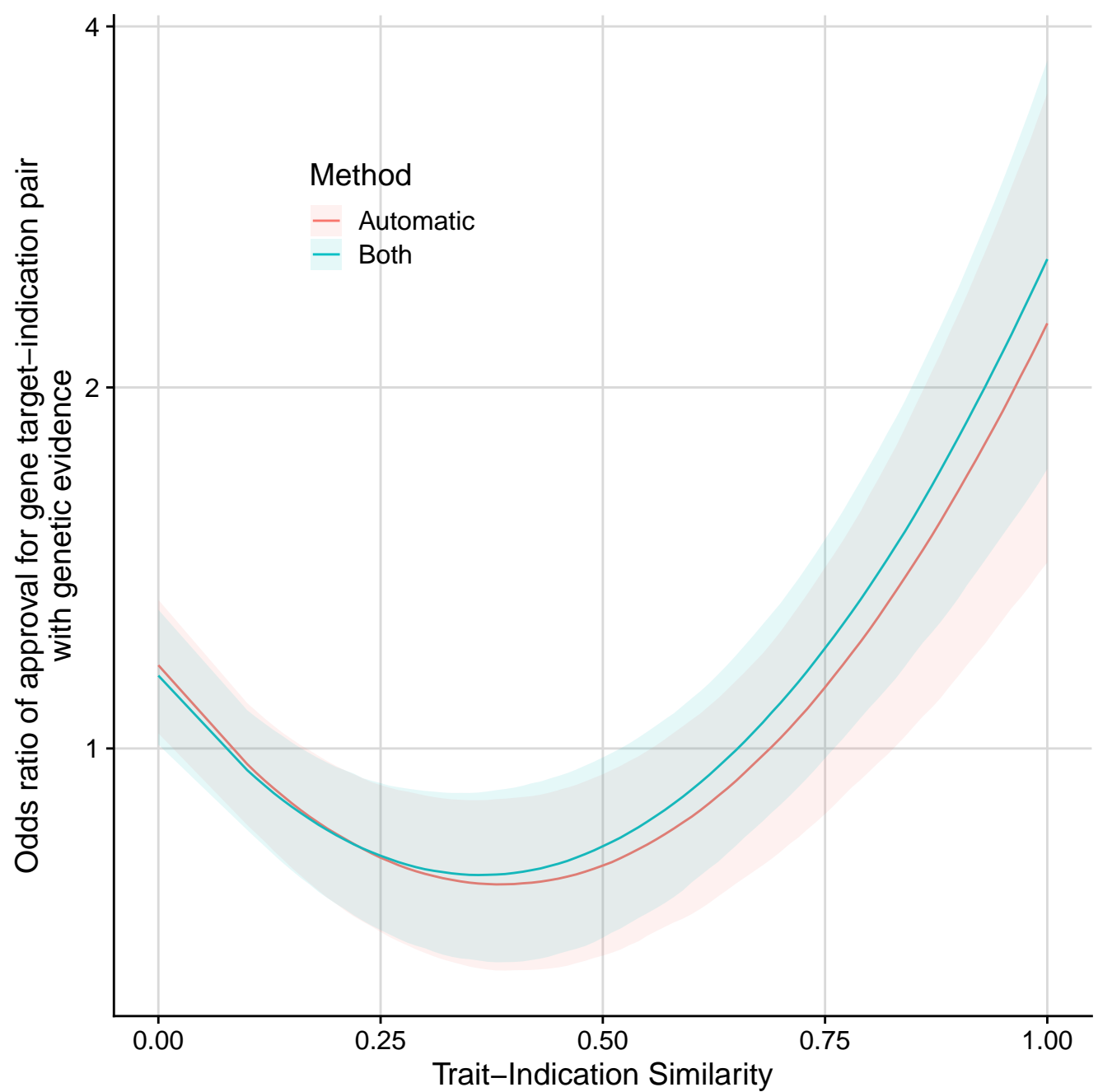

Supplement: S26 Fig — Effect of excluding manually assigned trait similarities on estimated relationship between GWAS genetic support and approval. Estimated odds ratio of gene target-indication pair attaining approval, as a function of similarity between drug indication and the most similar trait associated with the target. The two colors correspond to estimates when using and when excluding manually assigned similarities. Posterior median and pointwise 95% credible interval from Bayesian logistic regression. (PDF) [file pgen.1008489.s031.pdf]
