# Supplementary material for: Are drug targets with genetic support twice as likely to be approved? Revised estimates of the impact of genetic support for drug mechanisms on the probability of drug approval
Source: PLoS Genet. 2019 Dec 12;15(12):e1008489. doi: 10.1371/journal.pgen.1008489 (PMC6907751; doi:10.1371/journal.pgen.1008489)
Supplement: S6 Fig — Latest change date for Pharmaprojects drugs by development status. In panel Pharmaprojects Global Status, statuses come from the Pharmaprojects global status field. In panel Global Latest Phase, statuses are the latest global development phase assigned in this document. (PDF) [file pgen.1008489.s011.pdf]

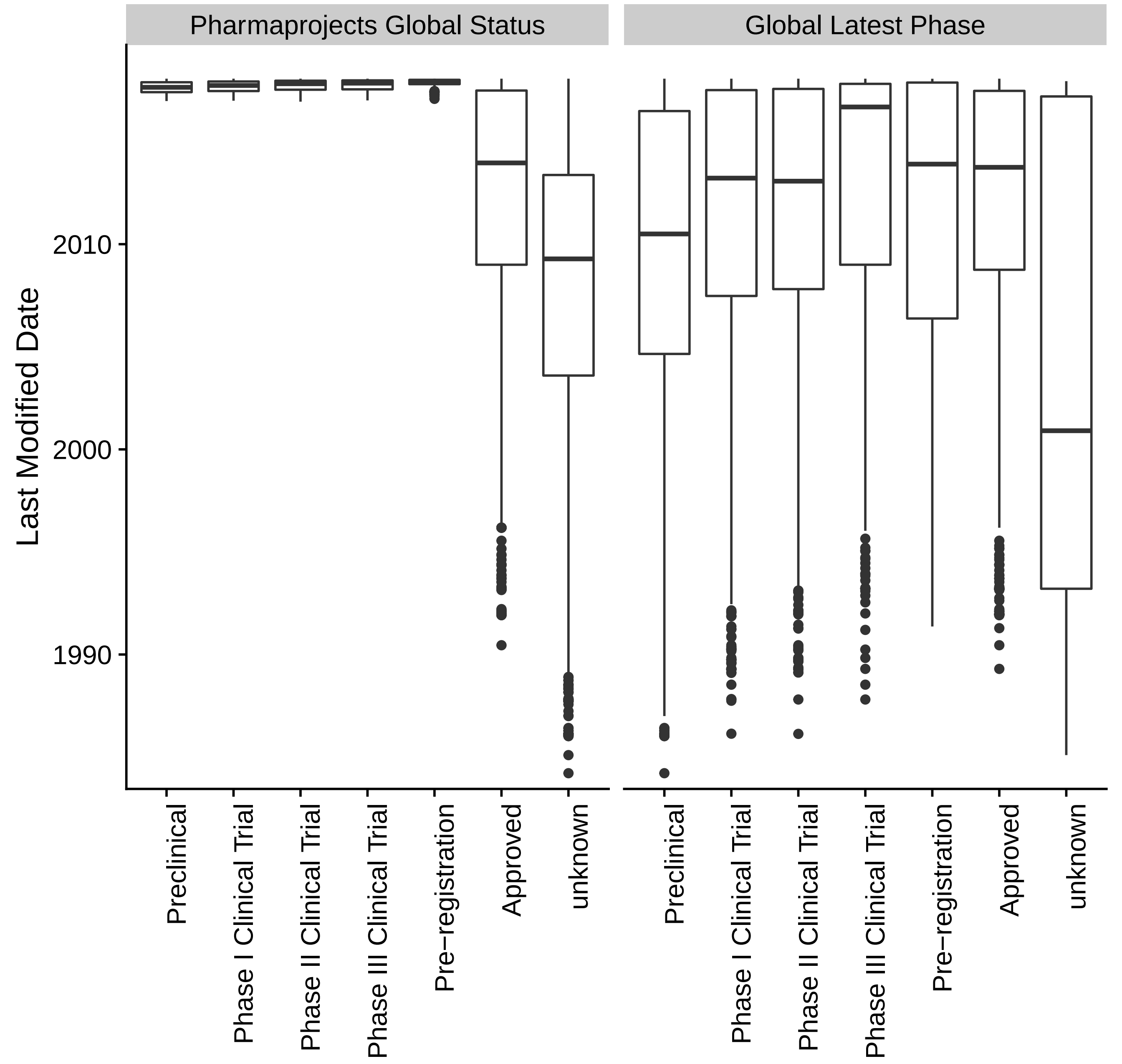

Pharmaprojects Global Status
Global Latest Phase
Last Modified Date
2010
2000
1990
Preclinical
Phase I Clinical Trial
Phase II Clinical Trial
Phase III Clinical Trial
Pre−registration
Approved
unknown
