# Supplementary material for: Are drug targets with genetic support twice as likely to be approved? Revised estimates of the impact of genetic support for drug mechanisms on the probability of drug approval
Source: PLoS Genet. 2019 Dec 12;15(12):e1008489. doi: 10.1371/journal.pgen.1008489 (PMC6907751; doi:10.1371/journal.pgen.1008489)
Supplement: S27 Fig — Estimated effect of OMIM genetic evidence on target-indication pair approval, excluding congenital diseases and indications with mapped MeSH term also mapped to an OMIM indication. Posterior median and 95% credible intervals based on 8907 target-indication pairs, compared to results from the full data with 20292 target-indication pairs. (PDF) [file pgen.1008489.s032.pdf]

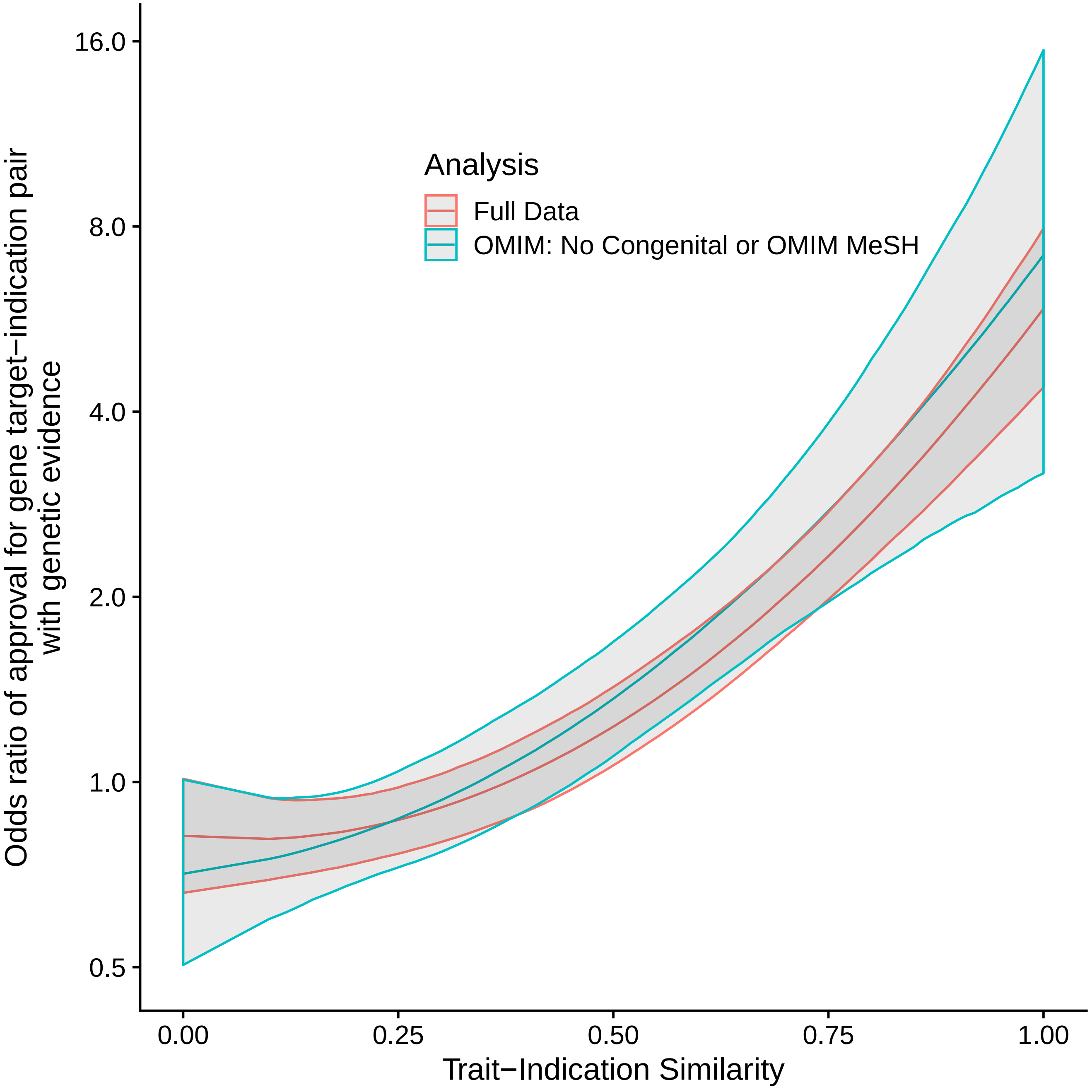

Analysis
Full Data
OMIM: No Congenital or OMIM MeSH
Odds ratio of approval for gene target−indication pair
with genetic evidence
16.0
8.0
4.0
2.0
1.0
0.5
0.00
0.25
0.50
0.75
1.00
Trait−Indication Similarity
