# Supplementary material for: Are drug targets with genetic support twice as likely to be approved? Revised estimates of the impact of genetic support for drug mechanisms on the probability of drug approval
Source: PLoS Genet. 2019 Dec 12;15(12):e1008489. doi: 10.1371/journal.pgen.1008489 (PMC6907751; doi:10.1371/journal.pgen.1008489)
Supplement: S1 Table — nassoc is the number of protein coding genes with genetic associations, napproved is the number of protein coding genes linked, naa is computed as the number of protein coding genes that are both the targets of approved drugs and have reported trait associations. (PDF) [file pgen.1008489.s033.pdf]

	No Genetic Association	Genetic Association
Not Approved	$n_{nn} = 22012 - n_{assoc} - n_{approved} + n_{aa}$	$n_{an} = n_{assoc.} - n_{aa}$
Approved	$n_{na} = n_{approved} - n_{aa}$	n_{aa}
