# Supplementary material for: Are drug targets with genetic support twice as likely to be approved? Revised estimates of the impact of genetic support for drug mechanisms on the probability of drug approval
Source: PLoS Genet. 2019 Dec 12;15(12):e1008489. doi: 10.1371/journal.pgen.1008489 (PMC6907751; doi:10.1371/journal.pgen.1008489)
Supplement: S2 Table — Replication of Table 1N (association between genetic evidence and historical progression) from Nelson et al. supplementary datasets. Risk ratio p(approved | genetic support)/p(approved | no genetic support) and bootstrap 95% confidence intervals. (PDF) [file pgen.1008489.s034.pdf]

	GWASdb & OMIM	GWASdb	OMIM
Preclinical to Phase I	1.1 (1.1-1.2)	1.1 (1-1.1)	1.2 (1.1-1.2)
Phase I to Phase II	1.2 (1.1-1.3)	1.2 (1.1-1.3)	1.2 (1.1-1.3)
Phase II to Phase III	1.5 (1.3-1.7)	1.4 (1.1-1.7)	1.6 (1.3-1.9)
Phase III to Approved	1.1 (1-1.2)	1 (0.8-1.2)	1.1 (0.9-1.3)
Phase I to Phase III	1.8 (1.5-2.1)	1.7 (1.4-2.1)	1.9 (1.5-2.3)
Phase I to Approved	2 (1.6-2.4)	1.7 (1.3-2.2)	2.2 (1.6-2.8)
