# Supplementary material for: Are drug targets with genetic support twice as likely to be approved? Revised estimates of the impact of genetic support for drug mechanisms on the probability of drug approval
Source: PLoS Genet. 2019 Dec 12;15(12):e1008489. doi: 10.1371/journal.pgen.1008489 (PMC6907751; doi:10.1371/journal.pgen.1008489)
Supplement: S3 Table — Proportion of drug-indication pairs (Indication) or drugs (Global) having development status information available from each source. Event = Pharmaprojects event history, Info = Pharmaprojects clinical information fields, Country = Pharmaprojects country status, Global = Indication status inferred from global status. (PDF) [file pgen.1008489.s035.pdf]

Source	Indication	Global
Event	0.06	0.58
Global	0.54	
Info	0.17	0.74
Country		0.98
