# Supplementary material for: Are drug targets with genetic support twice as likely to be approved? Revised estimates of the impact of genetic support for drug mechanisms on the probability of drug approval
Source: PLoS Genet. 2019 Dec 12;15(12):e1008489. doi: 10.1371/journal.pgen.1008489 (PMC6907751; doi:10.1371/journal.pgen.1008489)
Supplement: S4 Table — Agreement between Pharmaprojects status (Type = Global or Indication) and latest phase using each evidence source when both are assigned a known development status. Columns less, greater, and equal are the proportion of times in which the source implicates a latest pipeline phase less advanced than, more advanced than, or equal to that reported by Pharmaprojects. Arranged in order of decreasing agreement. (PDF) [file pgen.1008489.s036.pdf]

| Source | Type | Less | Greater | Equal |
|---|---|---|---|---|
| Global | Indication | 0.00 | 0.00 | 1.00 |
| Country | Global | 0.01 | 0.00 | 0.99 |
| Event | Global | 0.01 | 0.02 | 0.97 |
| Event | Indication | 0.08 | 0.02 | 0.91 |
| Info | Global | 0.46 | 0.02 | 0.53 |
| Info | Indication | 0.50 | 0.02 | 0.48 |
