# Supplementary material for: Are drug targets with genetic support twice as likely to be approved? Revised estimates of the impact of genetic support for drug mechanisms on the probability of drug approval
Source: PLoS Genet. 2019 Dec 12;15(12):e1008489. doi: 10.1371/journal.pgen.1008489 (PMC6907751; doi:10.1371/journal.pgen.1008489)
Supplement: S5 Table — Replication of Table 1N (association between genetic evidence and historical progression) from Nelson et al. supplementary genetic association dataset and updated pipeline data. Risk ratio p(approved | genetic support)/p(approved | no genetic support) and bootstrap 95% confidence intervals. (PDF) [file pgen.1008489.s037.pdf]

	GWASdb & OMIM	GWASdb	OMIM
Preclinical to Phase I	1.1 (1.1-1.2)	1.1 (1-1.1)	1.2 (1.1-1.4)
Phase I to Phase II	1.1 (1-1.2)	1.1 (1-1.1)	1.2 (1.1-1.2)
Phase II to Phase III	1.4 (1.3-1.6)	1.2 (1-1.4)	1.8 (1.5-2)
Phase III to Approved	1.2 (1.1-1.3)	1.2 (1-1.3)	1.3 (1.1-1.4)
Phase I to Phase III	1.6 (1.4-1.8)	1.3 (1-1.5)	2.1 (1.8-2.4)
Phase I to Approved	2 (1.6-2.3)	1.5 (1.1-1.9)	2.6 (2.1-3.2)
