# Supplementary material for: Are drug targets with genetic support twice as likely to be approved? Revised estimates of the impact of genetic support for drug mechanisms on the probability of drug approval
Source: PLoS Genet. 2019 Dec 12;15(12):e1008489. doi: 10.1371/journal.pgen.1008489 (PMC6907751; doi:10.1371/journal.pgen.1008489)
Supplement: S6 Table — Risk ratio of pipeline progression from 2013 to 2018 by presence or absence of supporting genetic evidence and 2013 phase. Risk ratio and 95% confidence intervals. Last column gives the total number of gene target-indication pairs labeled with that phase in 2013 and the total number of gene target-indication pairs that progressed in development. (PDF) [file pgen.1008489.s038.pdf]

Event	GWASdb & OMIM	GWASdb	OMIM	N
Preclinical to Phase I	1.7 (0.8-2.6)	1.8 (0.8-2.9)	1.8 (0.5-3.2)	829 (207)
Phase I to Phase II	1.6 (1.1-2.2)	1.5 (0.7-2.2)	1.9 (1.1-2.7)	986 (362)
Phase II to Phase III	1.6 (0.9-2.3)	0.4 (0-1)	2.8 (1.7-4.1)	1532 (250)
Phase III to Approved	1.5 (0.8-2.3)	1.5 (0.6-2.4)	1.2 (0.3-2.3)	341 (125)
