# Supplementary material for: Are drug targets with genetic support twice as likely to be approved? Revised estimates of the impact of genetic support for drug mechanisms on the probability of drug approval
Source: PLoS Genet. 2019 Dec 12;15(12):e1008489. doi: 10.1371/journal.pgen.1008489 (PMC6907751; doi:10.1371/journal.pgen.1008489)
Supplement: S7 Table — Risk ratio of pipeline progression from 2013 to 2018 by presence or absence of supporting genetic evidence. Risk ratio and 95% confidence intervals. Last column gives the total number of 2013 gene target-indication pairs included in the analysis and the total number of drugs that progressed in development. This table shows the risk ratio of progression to a higher phase or to approval from any starting phase. (PDF) [file pgen.1008489.s039.pdf]

| Event | GWASdb & OMIM | GWASdb | OMIM | N |
|---|---|---|---|---|
| Progression | 1.5 (1.1-1.8) | 1.2 (0.8-1.6) | 1.9 (1.4-2.4) | 3842 (961) |
| Approval | 2.5 (1.4-4) | 2.5 (1.1-4.1) | 2.5 (1-4.6) | 3842 (172) |
