# Supplementary material for: Are drug targets with genetic support twice as likely to be approved? Revised estimates of the impact of genetic support for drug mechanisms on the probability of drug approval
Source: PLoS Genet. 2019 Dec 12;15(12):e1008489. doi: 10.1371/journal.pgen.1008489 (PMC6907751; doi:10.1371/journal.pgen.1008489)
Supplement: S8 Table — Replication of Table 1N (association between genetic evidence and historical progression) from Nelson et al. 2015 supplementary genetic association dataset and updated pipeline data, using only gene target-indication pairs not used in the original analysis either due to not being in the table of gene target-indication pairs or having an inactive development status. Risk ratio p(approved | genetic support)/p(approved | no genetic support) and bootstrap 95% confidence intervals. (PDF) [file pgen.1008489.s040.pdf]

	GWASdb & OMIM	GWASdb	OMIM
Preclinical to Phase I	1.1 (1-1.2)	1.1 (1-1.2)	1.1 (0.9-1.3)
Phase I to Phase II	1 (0.9-1.1)	1 (0.9-1.2)	0.9 (0.7-1.1)
Phase II to Phase III	1.3 (0.8-1.7)	1.3 (0.8-1.8)	1.3 (0.6-2.2)
Phase III to Approved	1.9 (1.1-2.7)	1.7 (0.9-2.5)	2.8 (1.5-3.9)
Phase I to Phase III	1.3 (0.8-1.8)	1.3 (0.8-1.9)	1.2 (0.5-2.1)
Phase I to Approved	2.4 (1.2-3.9)	2.2 (0.9-3.8)	3.4 (1.1-6.6)
