# Supplementary material for: Are drug targets with genetic support twice as likely to be approved? Revised estimates of the impact of genetic support for drug mechanisms on the probability of drug approval
Source: PLoS Genet. 2019 Dec 12;15(12):e1008489. doi: 10.1371/journal.pgen.1008489 (PMC6907751; doi:10.1371/journal.pgen.1008489)
Supplement: S9 Table — Replication of Table 1N (association between genetic evidence and historical progression) from Nelson et al. 2015 supplementary genetic association dataset and updated pipeline data, using only gene target-indication pairs not used in the original analysis due to not being in the table of gene target-indication pairs. Risk ratio p(approved | genetic support)/p(approved | no genetic support) and bootstrap 95% confidence intervals. (PDF) [file pgen.1008489.s041.pdf]

	GWASdb & OMIM	GWASdb	OMIM
Preclinical to Phase I	1.2 (1-1.4)	1.3 (1.1-1.5)	0.9 (0.6-1.2)
Phase I to Phase II	1 (0.9-1.2)	1 (0.9-1.2)	1 (0.7-1.4)
Phase II to Phase III	1.6 (1-2.4)	1.4 (0.8-2.3)	3 (1.4-4.7)
Phase III to Approved	1.5 (0.7-2.5)	1.4 (0.5-2.5)	2.3 (1-3.7)
Phase I to Phase III	1.7 (1-2.5)	1.5 (0.7-2.4)	3 (1-5.2)
Phase I to Approved	2.5 (0.9-4.7)	2.1 (0.4-4.3)	7 (1.6-14.3)
