# Supplementary material for: Are drug targets with genetic support twice as likely to be approved? Revised estimates of the impact of genetic support for drug mechanisms on the probability of drug approval
Source: PLoS Genet. 2019 Dec 12;15(12):e1008489. doi: 10.1371/journal.pgen.1008489 (PMC6907751; doi:10.1371/journal.pgen.1008489)
Supplement: S10 Table — Replication of Table 1N (association between genetic evidence and historical progression) from Nelson et al. 2015 supplementary genetic association dataset and updated pipeline data, using only gene target-indication pairs not used in the original analysis due to being assigned an inactive status. Risk ratio p(approved | genetic support)/p(approved | no genetic support) and bootstrap 95% confidence intervals. (PDF) [file pgen.1008489.s042.pdf]

	GWASdb & OMIM	GWASdb	OMIM
Preclinical to Phase I	1.1 (0.9-1.3)	0.9 (0.7-1.1)	1.4 (1.1-1.8)
Phase I to Phase II	1 (0.8-1.2)	1 (0.8-1.2)	0.9 (0.6-1.2)
Phase II to Phase III	0.9 (0.5-1.5)	1.2 (0.5-2)	0.5 (0-1.2)
Phase III to Approved	2.5 (1.1-3.9)	2 (0.5-3.5)	4 (3.3-5)
Phase I to Phase III	0.9 (0.4-1.5)	1.2 (0.5-2)	0.4 (0-1)
Phase I to Approved	2.3 (0.7-4.4)	2.4 (0.5-5.1)	1.6 (0-4.3)
