# Supplementary material for: Are drug targets with genetic support twice as likely to be approved? Revised estimates of the impact of genetic support for drug mechanisms on the probability of drug approval
Source: PLoS Genet. 2019 Dec 12;15(12):e1008489. doi: 10.1371/journal.pgen.1008489 (PMC6907751; doi:10.1371/journal.pgen.1008489)
Supplement: S11 Table — Risk ratio of progression in clinical development from 2013 to 2018 by presence or absence of supporting genetic evidence. Calculations are performed on the subset of target-indication pairs without similar 2013 approved target-indication pairs, using similarity cutoff 0.73. Risk ratio and 95% confidence intervals. (PDF) [file pgen.1008489.s043.pdf]

Event	GWASdb & OMIM	GWASdb	OMIM	N
Preclinical to Phase I	1.9 (1-2.8)	1.8 (0.8-2.9)	2.3 (0.6-3.9)	818 (202)
Phase I to Phase II	1.7 (1.1-2.3)	1.5 (0.7-2.2)	2 (1.1-2.7)	968 (350)
Phase II to Phase III	1.6 (0.8-2.4)	0.4 (0-1)	3.2 (1.8-4.7)	1442 (231)
Phase III to Approved	1.6 (0.9-2.4)	1.5 (0.7-2.5)	1.3 (0.3-2.5)	289 (102)
