# Supplementary material for: Are drug targets with genetic support twice as likely to be approved? Revised estimates of the impact of genetic support for drug mechanisms on the probability of drug approval
Source: PLoS Genet. 2019 Dec 12;15(12):e1008489. doi: 10.1371/journal.pgen.1008489 (PMC6907751; doi:10.1371/journal.pgen.1008489)
Supplement: S12 Table — Risk ratio of progression in clinical development from 2013 to 2018 by presence or absence of supporting genetic evidence. Calculations are performed on the subset of target-indication pairs with no approved 2013 drugs for that target. Risk ratio and 95% confidence intervals. (PDF) [file pgen.1008489.s044.pdf]

Event	GWASdb & OMIM	GWASdb	OMIM	N
Preclinical to Phase I	2.1 (1-3.3)	2 (0.8-3.3)	2.3 (0.7-4.1)	633 (140)
Phase I to Phase II	1.6 (0.9-2.5)	1.7 (0.7-2.7)	1.8 (0.5-2.9)	655 (215)
Phase II to Phase III	2.2 (1-3.6)	0.3 (0-1.1)	5.5 (3.3-7.9)	754 (106)
Phase III to Approved	0.9 (0.3-1.6)	1.1 (0.4-2.1)	0.4 (0-1.2)	100 (45)
