# Supplementary material for: Are drug targets with genetic support twice as likely to be approved? Revised estimates of the impact of genetic support for drug mechanisms on the probability of drug approval
Source: PLoS Genet. 2019 Dec 12;15(12):e1008489. doi: 10.1371/journal.pgen.1008489 (PMC6907751; doi:10.1371/journal.pgen.1008489)
Supplement: S13 Table — Replication of Table 1N (association between genetic evidence and historical progression) from Nelson et al. supplementary genetic association dataset and updated pipeline data with all MeSH terms assigned valid headings (All OMIM). Column New OMIM shows results using only OMIM entries originally mapped to supplementary concepts (and therefore not used in Table 1N). Risk ratio p(approved | genetic support)/p(approved | no genetic support) and bootstrap 95% confidence intervals. (PDF) [file pgen.1008489.s045.pdf]

	All OMIM	New OMIM Only
Preclinical to Phase I	1.1 (1.1-1.2)	1.1 (0.9-1.2)
Phase I to Phase II	1.2 (1.1-1.3)	1.2 (1.1-1.3)
Phase II to Phase III	1.6 (1.4-1.8)	1.4 (1.1-1.8)
Phase III to Approved	1.3 (1.2-1.4)	1.3 (1.1-1.5)
Phase I to Phase III	1.9 (1.7-2.2)	1.7 (1.3-2.2)
Phase I to Approved	2.5 (2.1-2.9)	2.2 (1.6-2.9)
