# Supplementary material for: Are drug targets with genetic support twice as likely to be approved? Revised estimates of the impact of genetic support for drug mechanisms on the probability of drug approval
Source: PLoS Genet. 2019 Dec 12;15(12):e1008489. doi: 10.1371/journal.pgen.1008489 (PMC6907751; doi:10.1371/journal.pgen.1008489)
Supplement: S14 Table — Sources used to obtain and annotate GWAS variants. Filter gives the p-value or other cutoff used to filter results. (PDF) [file pgen.1008489.s046.pdf]

Dataset or Tool	Downloaded	Version	Filter	URL or file path
GWAS Catalog	Sept 25, 2017	v1.0.1	$p < 10^{-8}$	https://www.ebi.ac.uk/gwas/api/search/downloads/alternative
1000 Genomes		Phase 3	$r^2 \geq 0.5$	bochet.gcc.biostat.washington.edu/beagle/1000_Genomes_phase3_v5a
GTEx	Sept 26, 2017	v7	$p < 10^{-6}$	https://www.gtexportal.org/home/datasets, GTEx_Analysis_v7_eQTL.tar.gz
SnpEff			$d \leq 5000$	http://snpeff.sourceforge.net/
Regulatory Elements Database	Oct 4, 2017	v3	$p > 0.999$	http://big.databio.org/RED/allGeneCorrelations100000.p2_v3.txt.gz
RegulomeDB	Oct 23, 2017	v1.1		http://www.regulomedb.org/downloads/RegulomeDB.dbSNP141.txt.gz
