# Supplementary material for: Are drug targets with genetic support twice as likely to be approved? Revised estimates of the impact of genetic support for drug mechanisms on the probability of drug approval
Source: PLoS Genet. 2019 Dec 12;15(12):e1008489. doi: 10.1371/journal.pgen.1008489 (PMC6907751; doi:10.1371/journal.pgen.1008489)
Supplement: S15 Table — Comparison of counts of distinct genes, traits (MeSH), and SNPs reported by Nelson et al. and those from the current analysis. Overlap is the number of items in common. (PDF) [file pgen.1008489.s047.pdf]

Property	Previous	Current	Overlap
Ensembl Genes	6045	8872	4255
MeSH	434	604	232
Associated SNPs	13933	10815	2732
LD SNPs	16270	18204	1562
LD SNP-Gene Links	20313	27208	1304
SNP-Gene Links	53526	48564	6678
MeSH-Gene Links	13702	36044	3535
MeSH-Gene-SNP Links	65923	62043	4325
