# Supplementary material for: Are drug targets with genetic support twice as likely to be approved? Revised estimates of the impact of genetic support for drug mechanisms on the probability of drug approval
Source: PLoS Genet. 2019 Dec 12;15(12):e1008489. doi: 10.1371/journal.pgen.1008489 (PMC6907751; doi:10.1371/journal.pgen.1008489)
Supplement: S16 Table — Comparison of counts of distinct genes, traits (MeSH), and SNPs reported by Nelson et al. and those from the current analysis, restricted to SNP associations expected to appear in both datasets (pre-May 21, 2013 GWAS Catalog associations). Overlap is the number of items in common. (PDF) [file pgen.1008489.s048.pdf]

Property	Previous	Current	Overlap
Ensembl Genes	3424	4020	2338
MeSH	334	345	199
Associated SNPs	3353	2690	1722
LD SNPs	5310	5742	787
LD SNP-Gene Links	6533	8467	674
SNP-Gene Links	10637	11741	4130
MeSH-Gene Links	6574	10316	2476
MeSH-Gene-SNP Links	11769	14139	2983
