# Supplementary material for: Are drug targets with genetic support twice as likely to be approved? Revised estimates of the impact of genetic support for drug mechanisms on the probability of drug approval
Source: PLoS Genet. 2019 Dec 12;15(12):e1008489. doi: 10.1371/journal.pgen.1008489 (PMC6907751; doi:10.1371/journal.pgen.1008489)
Supplement: S17 Table — Percent of LD SNP-Gene associations reported by Nelson et al. also found in the reanalysis by whether the association was an eQTL. Conditional on LD SNP presence in both analyses. (PDF) [file pgen.1008489.s049.pdf]

eQTL	Percent in Current Table
FALSE	85
TRUE	57
