# Supplementary material for: Are drug targets with genetic support twice as likely to be approved? Revised estimates of the impact of genetic support for drug mechanisms on the probability of drug approval
Source: PLoS Genet. 2019 Dec 12;15(12):e1008489. doi: 10.1371/journal.pgen.1008489 (PMC6907751; doi:10.1371/journal.pgen.1008489)
Supplement: S18 Table — Percent of LD SNP-Gene associations found in reanalysis also reported by Nelson et al. subdivided by what evidence source(s) were used to link the LD SNP and gene. Conditional on LD SNP presence in both analyses. (PDF) [file pgen.1008489.s050.pdf]

DHS	eQTL	distance	Percent in MN Table
TRUE	TRUE	TRUE	83
FALSE	TRUE	TRUE	79
TRUE	FALSE	TRUE	69
FALSE	FALSE	TRUE	82
TRUE	TRUE	FALSE	0
FALSE	TRUE	FALSE	5
TRUE	FALSE	FALSE	9
