# Supplementary material for: Are drug targets with genetic support twice as likely to be approved? Revised estimates of the impact of genetic support for drug mechanisms on the probability of drug approval
Source: PLoS Genet. 2019 Dec 12;15(12):e1008489. doi: 10.1371/journal.pgen.1008489 (PMC6907751; doi:10.1371/journal.pgen.1008489)
Supplement: S21 Table — Effect of OMIM genetic evidence on historical pipeline progression. Risk ratio p(approved | genetic support)/p(approved | no genetic support) and bootstrap 95% confidence intervals. Pharmaprojects drugs are subdivided by indication MeSH heading: Congenital indications are those indexed under Congenital, Hereditary, and Neonatal Diseases and Abnormalities and OMIM indications are those MeSH headings that are also mapped MeSH headings to an OMIM phenotype. (PDF) [file pgen.1008489.s053.pdf]

	No OMIM or Congenital Indication	Congenital Indication	OMIM Indication
Preclinical to Phase I	0.9 (0.7-1)	1.1 (0.9-1.3)	1.1 (1-1.2)
Phase I to Phase II	1.1 (1-1.2)	1.1 (1-1.2)	1.2 (1.2-1.3)
Phase II to Phase III	1.6 (1.2-2)	1.6 (1.3-2)	1.7 (1.5-1.9)
Phase III to Approved	1.4 (1.2-1.6)	1.7 (1.4-2.1)	1.4 (1.3-1.5)
Phase I to Phase III	1.8 (1.3-2.3)	1.8 (1.4-2.2)	2.1 (1.8-2.4)
Phase I to Approved	2.4 (1.7-3.2)	3 (2.2-4.1)	3 (2.5-3.4)
