# Supplementary material for: Are drug targets with genetic support twice as likely to be approved? Revised estimates of the impact of genetic support for drug mechanisms on the probability of drug approval
Source: PLoS Genet. 2019 Dec 12;15(12):e1008489. doi: 10.1371/journal.pgen.1008489 (PMC6907751; doi:10.1371/journal.pgen.1008489)
Supplement: S22 Table — Estimated effect of GWAS genetic evidence from case-control studies on historical pipeline progression by effect size (odds ratio). Risk ratio p(approved | genetic support)/p(approved | no genetic support) and bootstrap 95% confidence intervals. (PDF) [file pgen.1008489.s054.pdf]

	Case Control OR ≥ 1.2	Case Control OR < 1.2
Phase I to Phase II	1.1 (0.9-1.2)	1.1 (0.9-1.2)
Phase II to Phase III	0.9 (0.6-1.2)	1.1 (0.8-1.3)
Phase III to Approved	1.1 (0.8-1.4)	1.1 (0.8-1.3)
Phase I to Phase III	0.9 (0.6-1.3)	1.1 (0.8-1.4)
Phase I to Approved	1 (0.6-1.5)	1.2 (0.8-1.7)
