# Supplementary material for: Are drug targets with genetic support twice as likely to be approved? Revised estimates of the impact of genetic support for drug mechanisms on the probability of drug approval
Source: PLoS Genet. 2019 Dec 12;15(12):e1008489. doi: 10.1371/journal.pgen.1008489 (PMC6907751; doi:10.1371/journal.pgen.1008489)
Supplement: S23 Table — Similarity between different pairs of traits computed using Resnik similarities, Lin similarities, normalized Resnik similarities, and average of normalized Resnik and Lin similarities using number of descendants to compute information content. (PDF) [file pgen.1008489.s055.pdf]

Term 1	Term 2	sim_{res}	sim_{lin}	$sim_{res,norm}$	sim_{avg}
Heart Diseases	Heart Diseases	5.07	1	1	1
Andersen Syndrome	Andersen Syndrome	10.26	1	1	1
Cardiomyopathies	Arrhythmias, Cardiac	5.07	0.73	0.49	0.61
Cardiomyopathy, Dilated	Romano-Ward Syndrome	5.07	0.49	0.49	0.49
Andersen Syndrome	Romano-Ward Syndrome	8.87	0.86	0.86	0.86
Arrhythmias, Cardiac	Atrial Fibrillation	6.57	0.78	0.64	0.71
